# Caspase-8 silences cell death-independent constitutive immune activation driven by tonic TNF-α

**DOI:** 10.1038/s44319-026-00813-5

**Published:** 2026-06-08

**Authors:** Germana Lentini, Francesco Coppolino, Agata Famà, Giuseppe Valerio De Gaetano, Federica Grasso, Luigi Fiore, Alessia Berbiglia, Silvia Carnevale, Isabella Venza, Egil Lien, Mario Venza, Sebastien Jaillon, Giuseppe Teti, Concetta Beninati

**Affiliations:** 1 Department of Human Pathology “Gaetano Barresi”, University of Messina, Messina, Italy; 2 Humanitas Clinical and Research Center IRCCS, Rozzano, Milan, Italy; 3 Department of Clinical and Experimental Medicine, University of Messina, Messina, Italy; 4 Division of Infectious Diseases and Immunology, Program in Innate Immunity, Department of Medicine, University of Massachusetts Medical School, Worcester, MA USA; 5 Centre of Molecular Inflammation Research, Department of Cancer Research and Molecular Medicine, Norwegian University of Science and Technology, Trondheim, Norway; 6 Department of Biomedical and Dental Sciences and Morphological and Functional Images, University of Messina, Messina, Italy; 7 Department of Molecular Medicine, Immunology and General Pathology Unit, University of Pavia, Pavia, Italy; 8 Scylla Biotech Srl, Messina, Italy

**Keywords:** Autophagy & Cell Death, Immunology, Signal Transduction

## Abstract

Caspase-8 is essential for maintaining organismal integrity by preventing cell death and subsequent inflammation in specific epithelial and endothelial tissues. Here, we show that caspase-8 also controls a systemic, cell death-independent inflammatory pathway that is constitutively active during homeostasis. In vivo, selective caspase-8 inhibition produces, in the absence of other stimuli, marked neutrophilia driven by circulating proinflammatory and chemotactic cytokines and promotes bacterial clearance during infection. In vitro, caspase-8 inhibition triggers in neutrophils, but not in macrophages, a profound transcriptional response associated with the release of IL-1β and other cytokines. This process requires tonic TNF-α production by neutrophils, which acts autocrinally to sequentially activate RIPK1, RIPK3, MAPKs, and NF-κB. The IL-1β release induced by caspase-8 inhibition requires gasdermin D and neutrophil serine proteases, but not canonical inflammasome components. Our data uncover the mechanistic features of a neutrophil-centric, proinflammatory pathway that can be therapeutically targeted to augment host defenses against pathogens.

## Synopsis

Caspase-8 controls a systemic, cell death-independent inflammatory pathway that is constitutively activated by autocrine TNF-α during homeostasis. Its inhibition triggers neutrophilia, IL-1β release, and has therapeutic effects against lethal bacterial infections.

- Tonic TNF-α drives profound proinflammatory changes in the neutrophil transcriptome.
- Caspase-8 silences these changes, which require TNFR1, RIPK1 and RIPK3 activation.
- Inhibiting caspase-8 unleashes TNF-α-driven cytokine release, causing neutrophilia.
- IL-1β release by caspase-8 blockade needs inflammasome-independent GSDMD processing.

## Abstract (web summary)

Caspase-8 controls a systemic, cell death-independent inflammatory pathway that is constitutively activated by autocrine TNF-α during homeostasis. Its inhibition triggers neutrophilia, IL-1β release, and has therapeutic effects against lethal bacterial infections.

## Graphical abstract

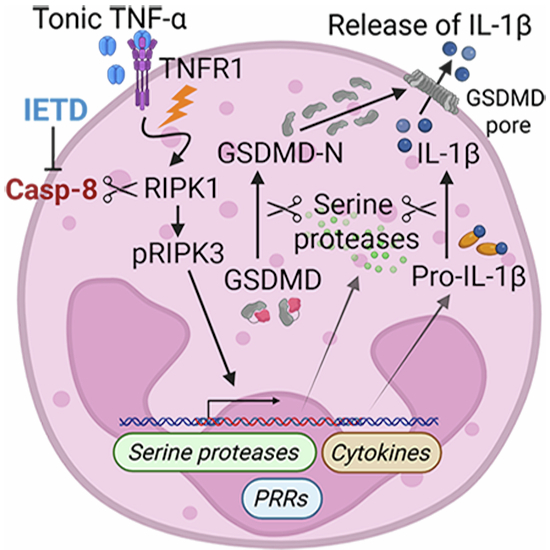

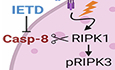

## Introduction

Host integrity depends on the recruitment of inflammatory cells to sites of pathogen entry and on the elimination of nonfunctional or infected cells by cell death programs, including apoptosis, pyroptosis, and necroptosis. Certain signaling molecules, such as cysteine-aspartic proteases (caspases) or receptor-interactive serine threonine kinases 1 and 3 (RIPK1 and RIPK3), are particularly relevant to both inflammation and cell death. These molecules are central to multiple activation pathways triggered by receptors that sense the presence of pathogens and tissue damage (Mancuso et al, 2009; Newton et al, 2021; Orning and Lien, 2021). Even in the absence of these danger signals—i.e., under homeostatic conditions—caspase-8 and RIPK1 play essential roles in maintaining tissue integrity by preventing spontaneously occurring cell death and inflammation in certain tissues. For example, these homeostatic functions are crucial for endothelial cell integrity during embryogenesis and, postnatally, for skin and intestinal epithelial cells as demonstrated by the deleterious consequences of genetic defects in caspase-8 or RIPK1 (Newton et al, 2024). RIPK1 is a widely expressed kinase with diverse functions, which vary depending on its enzymatic or scaffold function and the availability of different binding partners, such as adapter or effector molecules, within the cellular environment (Degterev et al, 2019). RIPK1 function is well-characterized in the context of signaling through tumor necrosis factor receptor type 1 (TNFR1), which responds to extracellular tumor necrosis factor-α (TNF-α), a crucial proinflammatory cytokine. TNFR1 activation results in the formation of a polyubiquitinated, multiprotein aggregate called complex I, in which RIPK1 has an essential scaffold function. Multiple members of complex I induce post-translational modifications (e.g., phosphorylation and polyubiquitination) of RIPK1, preventing its activation and promoting the production of pro-survival proteins, such as the long isoform of cellular FLICE inhibitory protein (c-FLIP_L_) via NF-κB transcription factor activation (Annibaldi and Meier, 2018; Kreuz et al, 2001). Despite being regulated by post-translational modifications, small amounts of RIPK1 become activated through autophosphorylation under physiological conditions. This activation results in the dissociation of RIPK1 from complex I and the formation of cytosolic complex-IIb aggregates containing the adapter FADD (Fas-associated protein with Death Domain) and caspase-8-cFLIP_L_heterodimers. These heterodimers play a crucial homeostatic role in cleaving RIPK1 and downregulating its activation (Feng et al, 2007; Lalaoui et al, 2020; Oberst et al, 2011). When caspase-8/cFLIP_L_ activity is inhibited or absent, active RIPK1 is free to activate RIPK3 and form large complexes with the downstream pseudokinase mixed lineage kinase domain-like protein MLKL, leading to necroptosis, an inflammatory form of cell death. Thus, under conditions of TNF-α stimulation, the survival of certain cells depends on post-transcriptional modifications of RIPK1 in complex I and on the proteolytic activity of cFLIP-caspase-8 heterodimers in complex IIb, both of which result in downregulation of RIPK1 activation.

Neutrophils are abundant, short-lived granulocytes that circulate in the bloodstream, patrol peripheral tissues, and play critical roles in antimicrobial defenses. Given their large numbers (about 500 billion in the body), any spontaneous proinflammatory activity in these cells must be tightly controlled to avoid the catastrophic consequences of generalized inflammation. We recently discovered that caspase-8 suppresses a constitutive proinflammatory program, which can be revealed by pharmacological caspase-8 inhibition (Lentini et al, 2023). However, the molecular mechanisms underlying this spontaneous, caspase-8-controlled inflammatory pathway remain only partially understood. Here we show that caspase-8 silences a cell-death-independent pathway driven by the constitutive production of small amounts of TNF-α, which acts as an autocrine via TNFR1to sequentially activate RIPK1 and RIPK3. RIPK3 activation/phosphorylation, in turn, leads to the transcription of a distinctive set of cell activation genes, including interleukin-1β (IL-1β), through mechanisms that are independent of MLKL, the downstream executor of necroptosis. Furthermore, mature IL-1β release depends on gasdermin D (GSDMD) activation via a pathway that is independent of canonical inflammasome components and instead requires the cooperative activity of elastase and cathepsin G serine proteases, which are stored in neutrophil granules. Our data reveal an unexpected, systemic proinflammatory pathway driven by tonic TNF-α production during adult homeostasis and regulated by caspase-8. In addition, pharmacological inhibition of caspase-8 can be leveraged therapeutically to enhance host defenses against bacterial infection.

## Results

### Acute caspase-8 inhibition produces neutrophilia through the release of G-CSF and CxcR2 ligands

**Figure 1:**
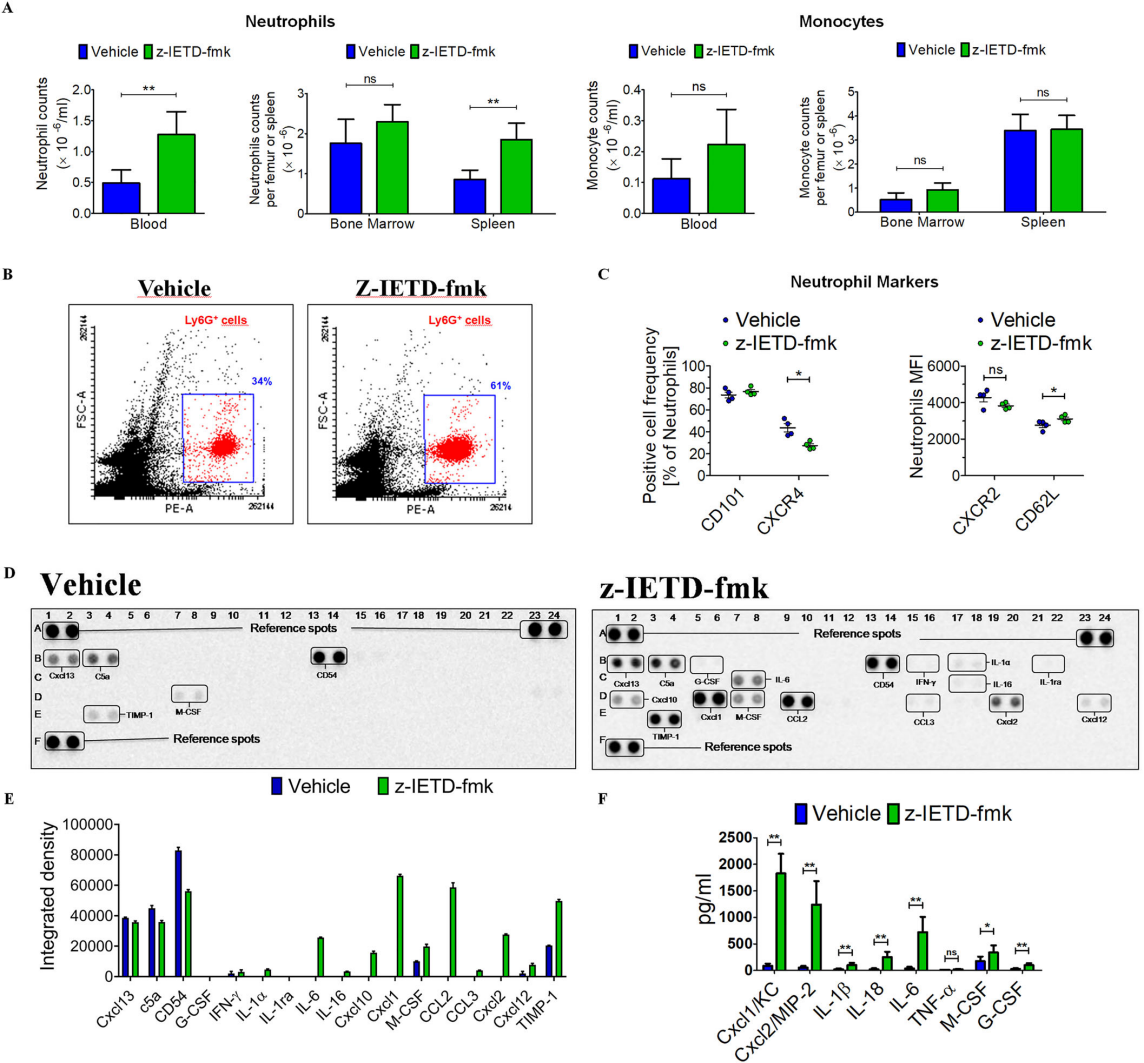
Caspase-8 inhibition with z-IETD-fmk induces neutrophilia in mice. Mice were inoculated i.p. with z-IETD-fmk (6 mg/kg in 0.2 ml) or vehicle (0.2 ml of 7.5% dimethyl sulfoxide in PBS), and blood, bone marrow, and spleen were collected at 4 h after inoculation. (**A**) Numbers of neutrophils and monocytes in the blood, bone marrow, and spleen (means + SDs of five determinations, each conducted in a different animal). (**B**) Immunofluorescence flow cytometry analysis of blood using phycoerythrin-conjugated antibodies directed against the Ly6G neutrophil marker. PE-A phycoerythrin area, FSC-A forward scatter area. (**C**) Immunofluorescence flow cytometry analysis of neutrophil surface markers in blood samples obtained at 4 h after z-IETD-fmk inoculation. MFI, mean fluorescence index. Points represent values obtained from four different animals, with bars indicating the mean. Error bars indicate standard deviations. (**D**) Membrane-based array analysis of cytokine plasma levels at 4 h after treatment with z-IETD-fmk or vehicle in the absence of additional stimuli. (**E**) Densitometric quantification of the cytokine arrays (*n* = 1 for each array) shown in (**D**). Columns and bars represent means + SDs of two technical replicates. (**F**) Cytokine plasma concentrations at 4 h after treatment with z-IETD-fmk or vehicle as determined by ELISA (means + SDs of five determinations, each conducted in a different animal). (**A**, **C**, **F**) *P < 0.05; **P < 0.01 by the Mann–Whitney test. ns non-significant. Source data are available online for this figure.

**Figure 2:**
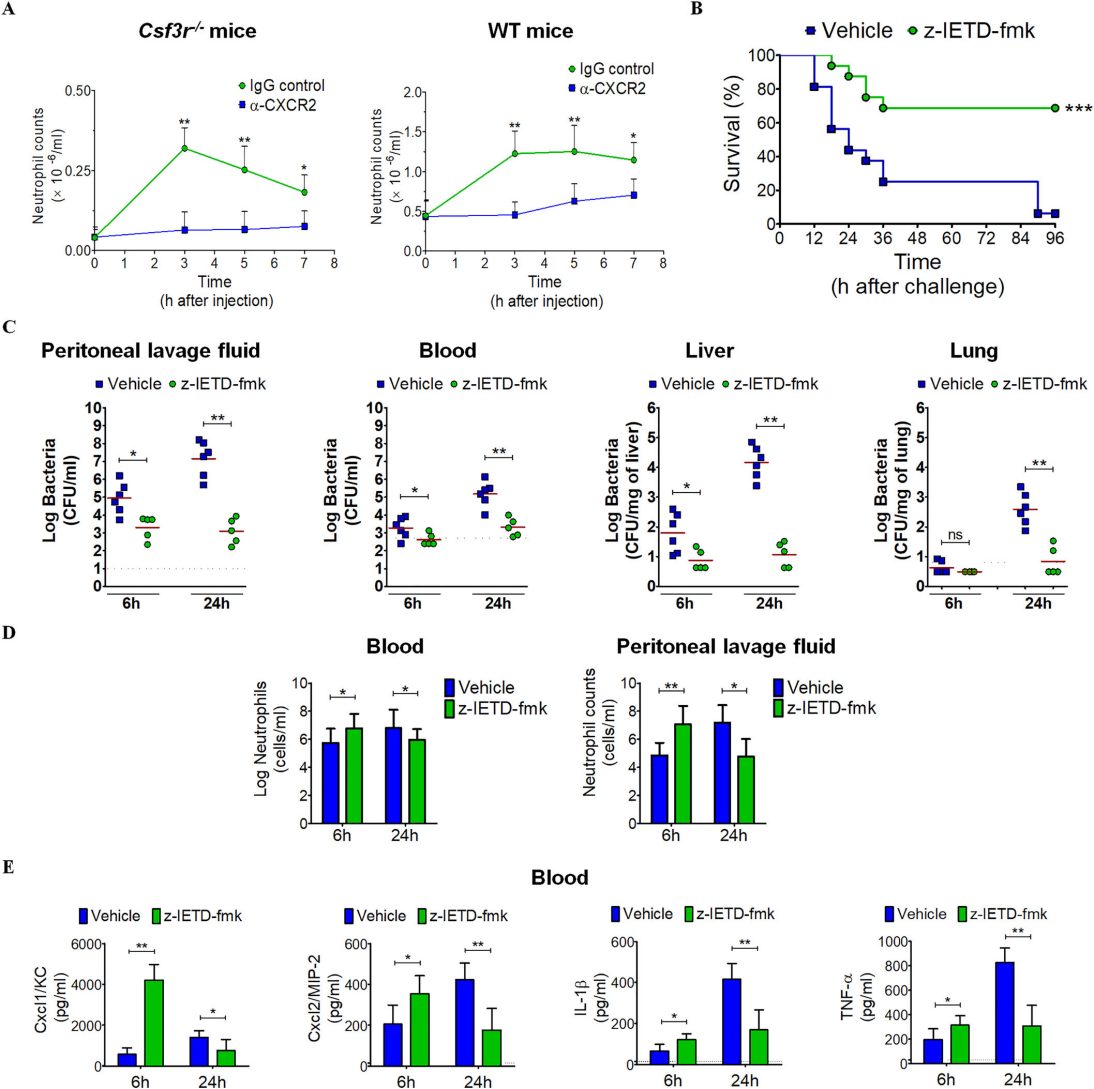
The neutrophilia induced by caspase-8 inhibition ameliorates lethal sepsis. (**A**) Effects of neutralizing antibodies directed against the CXCR2 receptor in mice lacking the G-CSF receptor (*Csf3r*^−/−^) and control wild-type (WT) mice. Mice were pretreated i.p. with anti-CXCR2 mAb (rat IgG_2A_) or rat IgG_2A_ isotype control (IgG control; both 6 mg/kg) 24 h before the i.p. administration of z-IETD-fmk or vehicle. Blood was collected at the indicated times after administration for measuring neutrophil counts, using five animals for each time point. Points and bars represent means +  SDs of five determinations, each conducted in a different animal. (**B**) Survival of mice treated i.p. with z-IETD-fmk or vehicle at 1 h after cecal ligation and puncture. (**C**–**E**) Mice were treated i.p. with z-IETD-fmk or vehicle at 1 h after cecal ligation and puncture. Blood, peritoneal lavage fluid, lungs, and liver were collected at 6 or 24 h post-infection to measure bacterial colony counts (**C**), neutrophil counts (**D**), and cytokine plasma levels (**E**). (**C**) Symbols represent values from individual animals; bars indicate mean values. (**D**, **E**) Columns and bars represent means plus SDs (*n* = 5 for each group). (**A**, **C**–**E**) *P < 0.05; **P < 0.01; ***P < 0.001 by the Mann–Whitney test. Source data are available online for this figure.

**Figure EV1:**
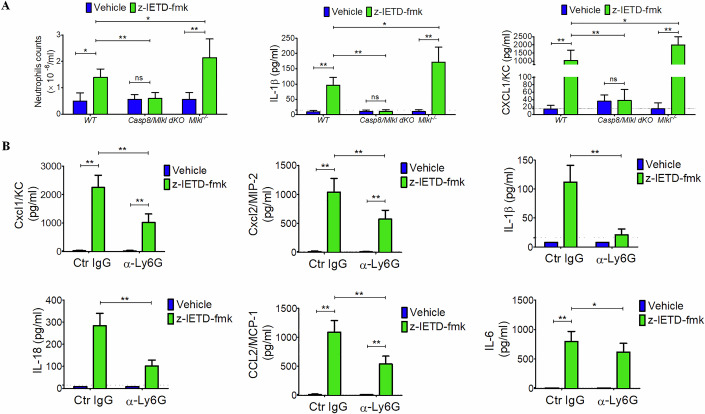
z-IETD-fmk-induced cytokine responses are due to specific caspase-8 inhibition and are significantly reduced after neutrophil depletion (related to Figs. 1 and 2). (**A**) Mice lacking MLKL (*Mlkl*^−/−^), both MLKL and caspase-8 (*Casp8/MLKL dKO*), or control wild-type (WT) mice were treated with z-IETD (6 mg/kg i.p.) or vehicle. Blood was collected 4 h later to determine neutrophil counts, plasma IL-1β, and plasma CXCL1 concentrations. Columns and bars represent the mean ± SDs of five determinations, each performed using samples from different animals, *P < 0.05; **P < 0.01 by the Mann–Whitney test. (**B**) Mice were pretreated with 100 μg of rat IgG_2A_mAb anti-mouse Ly6G (α-Ly6G) or rat IgG_2A_ isotype control (Ctr IgG) via i.p. injection 24 h prior to z-IETD-fmk or vehicle treatment. Blood was collected 4 h after inhibitor administration for measuring plasma cytokine levels. Columns and bars represent the mean ± SDs of five determinations, each conducted in a different animal. (**A**, **B**) *P < 0.05; **P < 0.01 by the Mann–Whitney test. Source data are available online for this figure.

We hypothesized that pharmacological blockade of caspase-8 might trigger systemic inflammatory responses, including leukocyte mobilization from hematopoietic tissues, a critical defense mechanism during infection. Surprisingly, treatment with the caspase-8 inhibitor z-IETD-fmk alone—without infection or other stimuli—was sufficient to more than double neutrophil (but not monocyte) numbers in both the blood and spleen (Fig. 1A,B). This neutrophilia was accompanied by the appearance of "*young”* (or freshly matured) neutrophils in the circulation (Fig. 1C), indicating their release from hematopoietic tissues. This interpretation was supported by analysis of phenotypic markers associated with neutrophil aging (Adrover et al, 2019; Casanova-Acebes et al, 2013), including increased CD62L expression and decreased CXCR4 levels after z-IETD-fmk administration (Fig. 1C). Importantly, neutrophilia was temporally associated with elevated circulating levels of several inflammatory mediators, including growth factors (G-CSF, M-CSF), cytokines (IL-1β, IL-6, and IL-18, but not TNF-α), and chemokines (Cxcl1 and Cxcl2; Fig. 1D–F). Since G-CSF and Cxcr2 agonists (such as Cxcl1 and Cxcl2) are potent inducers of neutrophil mobilization from the bone marrow (Semerad et al, 2002), we next examined the roles of the G-CSF receptor and Cxcr2 receptor in IETD-induced neutrophilia. In mice lacking the G-CSF receptor (*Csf3r*^−/−^), which displayed reduced basal neutrophil counts, IETD still induced an early, transient increase in circulating neutrophils (Fig. 2A). Notably, neutralizing anti-Cxcr2 antibodies completely abrogated IETD-induced increases in blood neutrophil counts in *Csf3r* KO animals, whereas in IETD-treated wild-type mice they only partially, albeit markedly, reduced this increase (Fig. 2A). These findings suggest that the neutrophilia induced by z-IETD-fmk is driven by the cooperative activities of G-CSF and CxcR2 ligands. To rule out off-target effects of IETD, we used caspase-8-deficient mice, specifically those lacking both caspase-8 and the necroptosis executioner MLKL, as isolated caspase-8 deficiency is embryonically lethal due to necroptosis (Tummers et al, 2020). As shown in Fig. EV1A, IETD-induced neutrophilia and IL-1β/Cxcl1 production were completely abrogated in caspase-8/MLKL double KO mice (but not in MLKL single KO mice), confirming that IETD specifically targets caspase-8 in the conditions used.

Collectively, these data demonstrate that the effects of z-IETD-fmk are due to specific caspase-8 inhibition, which promotes the release of inflammatory mediators through mechanisms distinct from necroptosis, ultimately leading to neutrophil mobilization from hematopoietic tissues into the bloodstream.

### Caspase-8 inhibition alleviates bacterial sepsis by enhancing neutrophil mobilization

We next explored whether the neutrophilia induced by caspase-8 inhibition could be harnessed to improve the outcome of severe bacterial infection. To this end, we used the cecal ligation puncture model, a well-established peritonitis/sepsis model that mimics key features of human sepsis (Rittirsch et al, 2009). Administration of z-IETD-fmk 1 h after challenge significantly reduced lethality (Fig. 2B), accompanied by an early increase in circulating cytokines, neutrophil blood counts, neutrophil recruitment to the peritoneal cavity, and bacterial clearance (Fig. 2C–E). Notably, at later time points, bacterial loads were markedly lower in the peritoneal cavity, blood, liver, and lungs of IETD-treated mice, resulting in reduced circulating cytokine levels and neutrophil counts compared to vehicle-treated controls (Fig. 2C–E). Together, these data suggest that the neutrophilia induced by caspase-8 inhibition can be therapeutically leveraged in a highly lethal sepsis model to promote bacterial clearance and enhance host survival.

### Caspase-8 inhibition profoundly affects the neutrophil but not the macrophage transcriptome

Next, to identify the cell types predominantly responsible for cytokine responses to IETD, mice were treated with anti-Ly6G antibodies, which resulted in nearly complete neutrophil depletion (blood neutrophil counts <1% of total leukocytes). Upon neutrophil depletion, IL-1β and IL-18 responses to the caspase-8 inhibitor were substantially decreased, whereas reductions in Cxcl1, Cxcl2, CCL2, and IL-6 levels were more modest but still significant (Fig. EV1B). Overall, these data suggest that neutrophils are at least partially involved in IETD-induced cytokine release. Since cytokine production is transcriptionally regulated, we next analyzed bone marrow-derived neutrophils for in vitro transcriptional responses to z-IETD-fmk in comparison with bone marrow-derived macrophages, which also represent a major cytokine-producing cell type.

**Figure 3:**
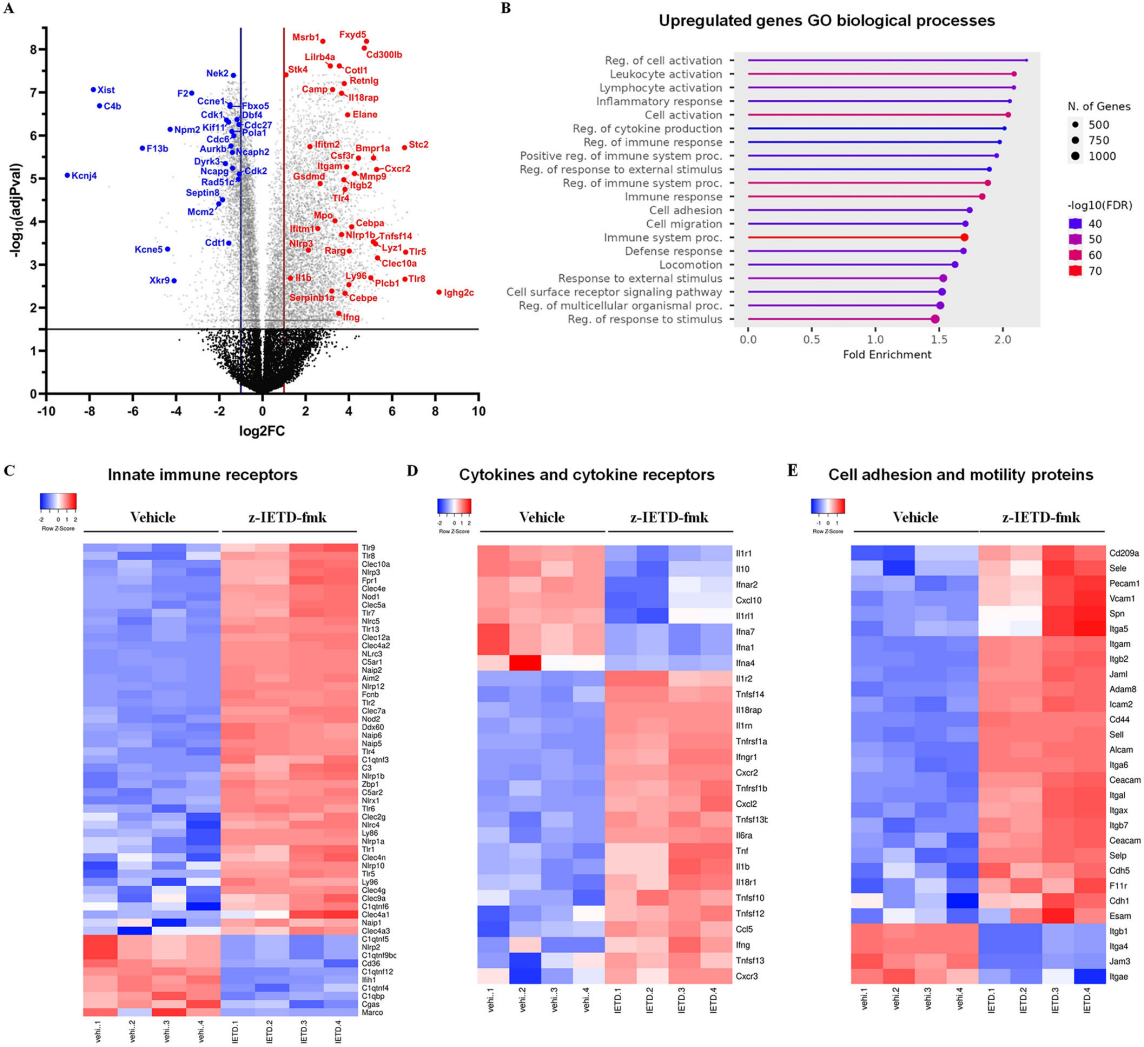
Caspase-8 inhibition induces extensive changes in the neutrophil transcriptome. Bone marrow-derived neutrophils were pretreated with z-IETD-fmk (50 μM) or vehicle for 3 h, followed by RNA extraction and sequencing. Shown are results from 4 separate experiments. (**A**) Volcano plot showing fold changes in gene expression in z-IETD-fmk-treated neutrophils compared with vehicle-treated neutrophils. Genes with a log2 fold change (log2FC) ≥ 1 and an adjusted *P* value (AdjPval) ≤ 0.05 were considered as differentially expressed. Selected upregulated (red) and downregulated genes (blue) are shown. (**B**) Gene expression profiling of IETD-upregulated neutrophil genes by Gene Ontology (GO) biological process enrichment analysis. FDR false discovery rate. See Fig. EV2 for enrichment analysis of IETD-downregulated genes. (**C**–**E**) Heatmaps of representative genes grouped by functional categories. IETD-upregulated and downregulated genes are shown in red and blue, respectively. See Fig. EV2 for heatmaps of additional gene groups. Source data are available online for this figure.

**Figure EV2:**
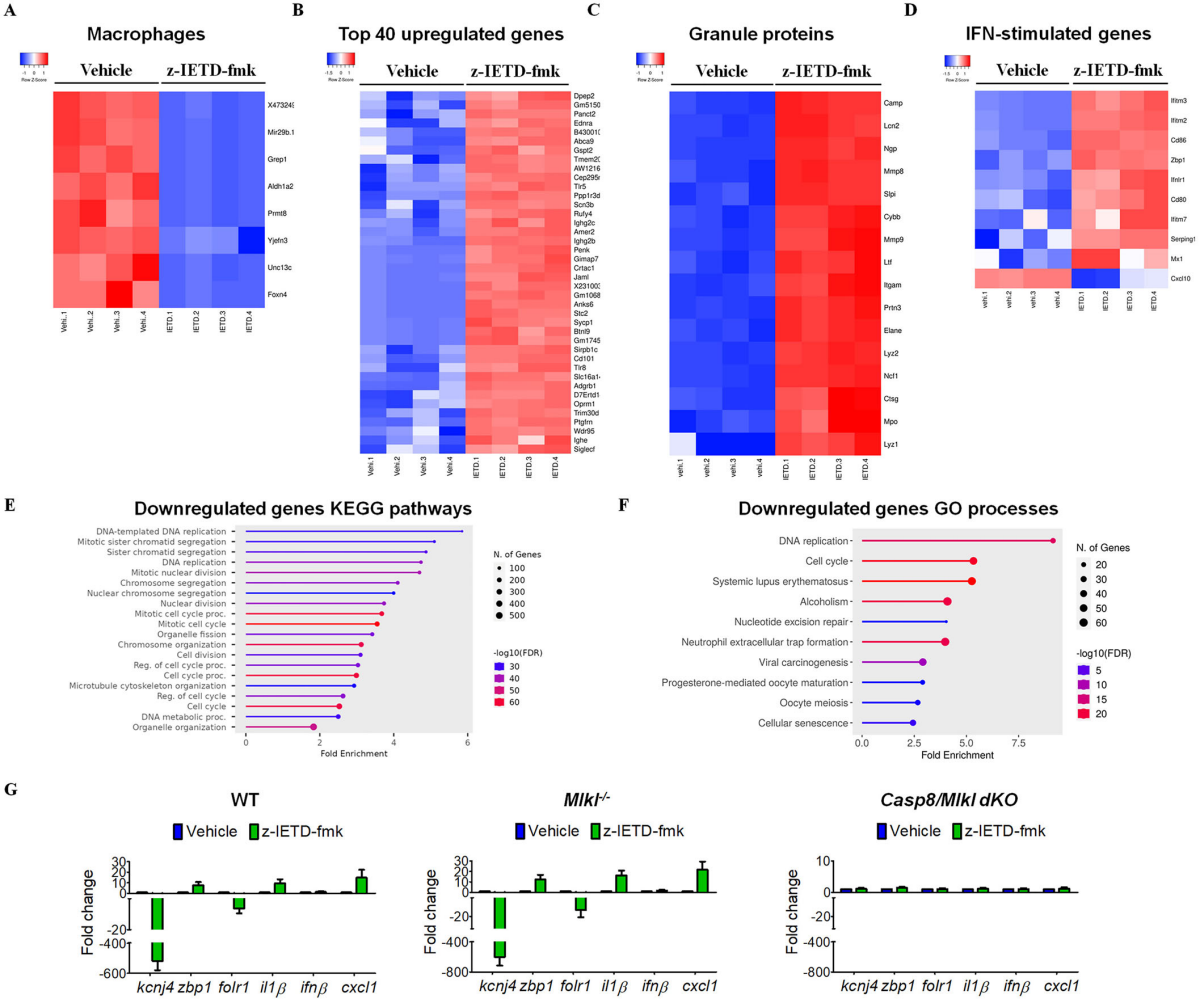
Caspase-8 inhibition alters the neutrophil transcriptome (related to Fig. 3). (**A**) Heatmap of significantly regulated genes in M-CSF-polarized macrophages exposed in vitro to z-IETD-fmk (50 μM) or vehicle. (**B**–**D**) Heatmaps of selected gene groups in bone marrow-derived neutrophils treated with z-IETD-fmk or vehicle. (**E**, **F**) Gene expression profiling by Gene Ontology (GO; (**E**) or KEGG pathway (**F**) enrichment analysis of genes downregulated by treatment with z-IETD-fmk in bone marrow-derived neutrophils. FDR, false discovery rate. (**G**) Real-time PCR analysis validation of RNA sequencing data using a panel of selected genes. Bone marrow-derived neutrophils obtained from mice lacking MLKL (*Mlkl*^−/−^), both MLKL and caspase-8 (*Casp8/Mlkl* dKO), or control wild-type (WT) animals were treated with z-IETD-fmk (50 μM for 3 h) or vehicle before RNA extraction and quantitative real-time PCR analysis. Source data are available online for this figure.

RNA sequencing analysis revealed that caspase-8 inhibition produces marked changes in the neutrophil transcriptome, with 5576 upregulated (log_2_ fold change ≥1 and adjusted *P* value < 0.05) and 1939 downregulated genes (Fig. 3A and GEO accession GSE288233). In striking contrast, only 8 genes (all moderately downregulated) were differentially expressed in macrophages (Fig. EV2A). Gene Ontology enrichment analysis of upregulated neutrophil genes identified functional categories associated with cell activation, including inflammatory response, cytokine production and regulation, immune response, cell adhesion, cell migration and receptor signaling (Fig. 3B). Highly (8-to 256-fold) upregulated genes encoded: pattern recognition receptors, co-receptors and damage associated molecular patterns (e.g., *Tlr4/5*/6/*8/13*, *Naip2*, *Nlrp1a/b*, *Nlrp12, Clec4g, Clec9a, Clec10a, Clec12a, Ly86, Ly96, S100G/A8/A9*; Figs. 3C and  EV2B); cytokines and cytokine receptors (e.g., *Ifng, Ccl6, Tnfsf14*,*Cxcr2, Csf3r, Ccr1, Ltbr, Il6ra, Il18rap*; Fig. 3D); cell adhesion and motility proteins (e.g., *Sell*, *Itgam, Itgb2, Fxyd5*, *Cd209a*, *Ceacam1*, *Ceacam2*, *Jaml*, *Adam8*, *Ednra*, *Adgrb1*, *Eml1*, *Actn1* (Fig. 3E); granule proteins (e.g., *Lyz1*, *Mmp8*, *Mmp9, Camp, Elane, Mpo, Retnlg;* Fig. EV2C); pro-survival factors (e.g., *Serpinb1a* and *Bmpr1a*); transcription factors involved in myeloid differentiation (e.g., *Cebpa*, *Cebpd*, *Cebpe, Hoxb3*); proteins involved in retinoic acid biosynthesis and signaling (e.g., *Rdh1*, *Rdh9*, *Aldh1a3* and *Rarg)*; long noncoding RNAs (e.g., *Gm5150, Gm1068* and *Gm1745*; Figs. 3A and  EV2B); and a set of IFN-stimulated genes (e.g., *Zbp1, Ifitm2/3/7*, *Cd80, Cd86, Mx1*; Fig. EV2D).

Gene Ontology and KEGG enrichment analysis of downregulated neutrophil genes revealed functions involved in DNA synthesis and repair and chromosome alignment (Fig. EV2E,F). Several downregulated RNA transcripts were represented by long noncoding RNAs or miRNAs (Fig. 3A and GEO accession GSE288233). To validate the RNA sequencing results and rule out off-target effects, expression of selected genes was assessed by RT-PCR in bone marrow-derived neutrophils from wild-type, caspase8/MLKL double KO, and MLKL single KO mice. IETD-induced changes in gene expression were observed in wild-type and MLKL-deficient, but not in double KO neutrophils (Fig. EV2G), confirming the caspase-8-specificity of our findings. Collectively, our data demonstrate that inhibition of the caspase-8 enzymatic activity is sufficient to trigger extensive transcriptional activation in neutrophils, but not in macrophages.

### Tonic TNF-α drives IETD-induced IL-1β production in neutrophils

**Figure EV3:**
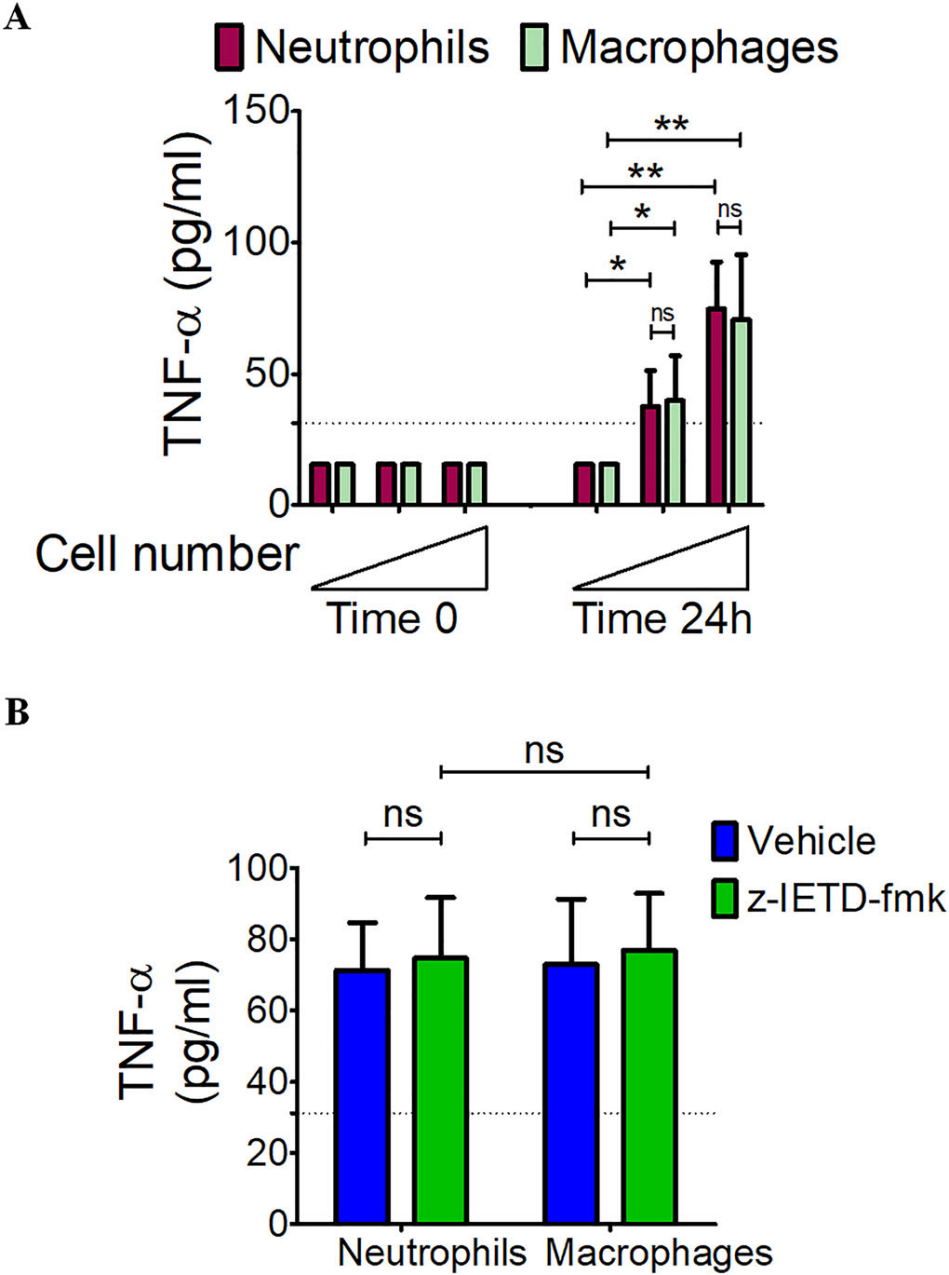
Neutrophils spontaneously produce TNF-α (related to Fig. 4). (**A**) Neutrophils or macrophages were cultured at increasing densities (2.5, 5, and 15 × 10⁶ cells/mL) for 24 h in the absence of stimuli, and TNF-α concentrations were measured in the culture supernatants. (**B**) Neutrophils or macrophages were cultured at a high cell density (15 × 10⁶ cells/mL) for 24 h in the presence of z-IETD-fmk or vehicle, and TNF-α concentrations were measured in the culture supernatants. Data are means + SDs (*n* = 5 biological replicates). (**A**, **B**) *P < 0.05; **P < 0.01 by the Mann–Whitney test. ns non-significant. Source data are available online for this figure.

**Figure 4:**
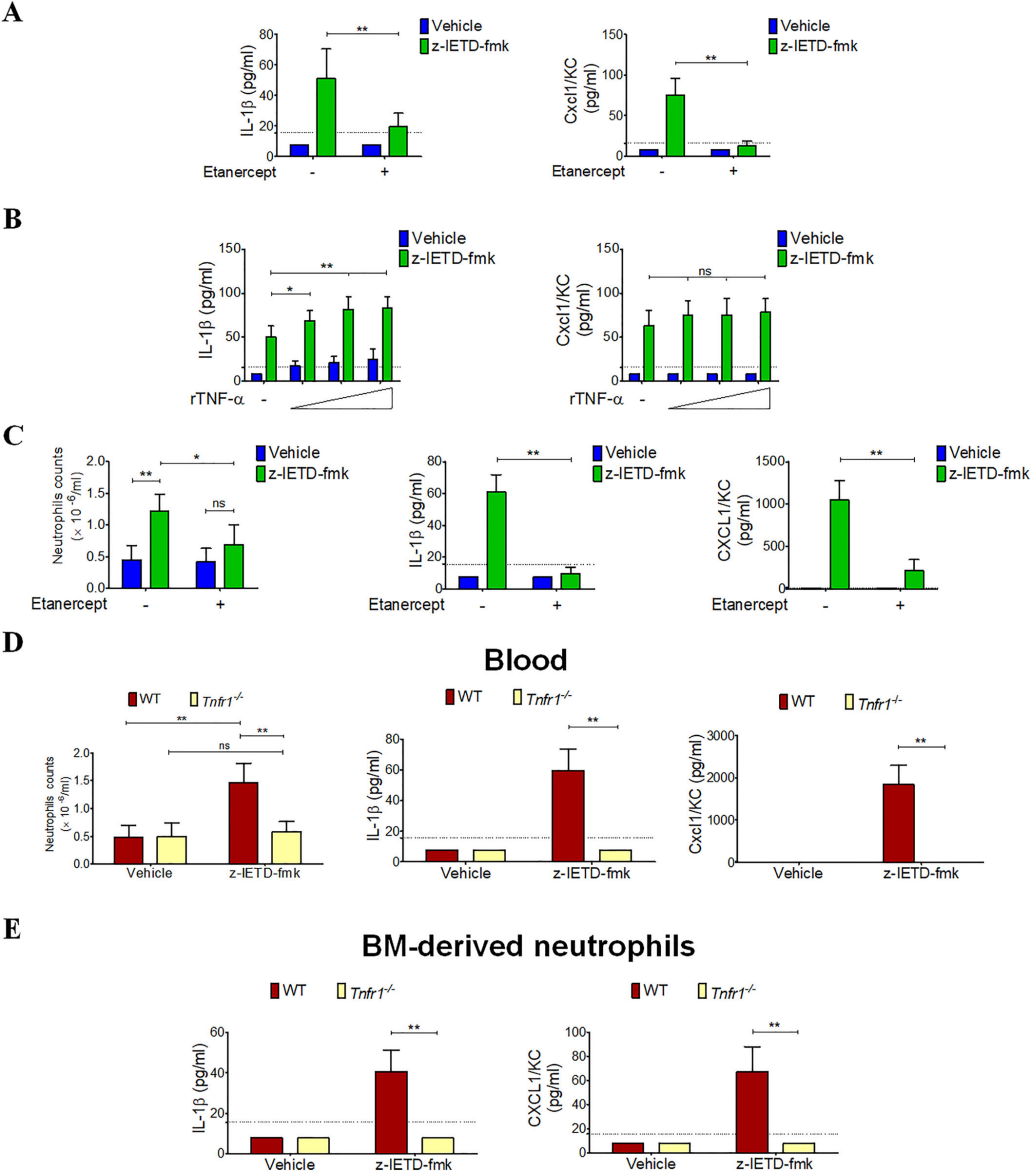
TNF-α and the type I TNF receptor drive cytokine release and neutrophilia upon caspase-8 inhibition. (**A**, **B**) Purified bone marrow-derived neutrophils were pretreated for 2 h with the TNF-α antagonist etanercept (**A**) or with increasing doses of mouse recombinant TNF-α (**B**), before the addition of z-IETD-fmk (50 μM) or vehicle. Samples of 24 h culture supernatants were tested for cytokine concentrations by ELISA. (**C**) Mice were pretreated with etanercept (6 mg/kg s.c.) or PBS 24 h prior to z-IETD-fmk administration. Neutrophil counts and plasma cytokine concentrations were measured in blood samples collected 4 h after administration of z-IETD-fmk (6 mg/kg i.p.). (**D**) Neutrophil counts and plasma cytokine concentrations were measured in blood samples collected 4 h after z-IETD-fmk administration in mice lacking the type I TNF receptor (*Tnfr1*^*−/−*^) or in control wild-type (WT) mice. (**E**) Bone marrow-derived neutrophils were obtained from *Tnfr1*^*−/−*^ or control WT mice and treated overnight with z-IETD-fmk (50 μM) or vehicle. Culture supernatants were tested for cytokine concentrations by ELISA. (**A**, **B**, **E**) Data are means +  SDs (*n* = 5 biological replicates). (**C**, **D**) Columns and bars represent means + SDs of five determinations, each conducted in a different animal. (**A**–**E**) **P < 0.01 by the Mann–Whitney test. ns non-significant. Source data are available online for this figure.

We next hypothesized that autocrine TNF-α is responsible for maintaining a state of constitutive activation in neutrophils, which is unleashed by caspase-8 inhibition. In support of this hypothesis, low, but detectable, levels of TNF-α were found in the supernatants of unstimulated cultures of highly purified bone marrow neutrophils, consistent with tonic TNF-α release (Fig. EV3A). These TNF-α levels were similar to those found in unstimulated macrophage cultures (Fig. EV3A), which have recently been shown to spontaneously release the cytokine (Luecke et al, 2023). Of note, treatment of neutrophils with z-IETD-fmk did not further increase TNF-α release over the levels observed in unstimulated cells (Fig. EV3B).To investigate the functional role of tonic TNF-α, neutrophil cultures were pretreated with the TNF antagonist etanercept prior to stimulation with IETD. Pretreatment with etanercept almost completely abrogated IETD-induced IL-1β and Cxcl1 release (Fig. 4A) and the exogenous addition of recombinant TNF-α potentiated IETD-induced IL-1β release (Fig. 4B). Similarly, in vivo administration of etanercept virtually abolished circulating cytokine elevations and significantly reduced neutrophilia in mice given z-IETD-fmk (Fig. 4C). To further assess the role of tonic TNF-α, we used mice lacking TNFR1, the primary TNF-α receptor. In contrast to wild-type mice, *Tnfr1*^−/−^ mice were completely unable to mount cytokine responses and did not exhibit neutrophilia in response to z-IETD-fmk administration (Fig. 4D). Moreover, *Tnfr1*^−/−^ neutrophils did not release cytokines in response to in vitro stimulation with the inhibitor (Fig. 4E). Collectively, these data indicate that tonic TNF-α production and autocrine TNFR1 signaling are essential for driving the production of IL-1β and other proinflammatory mediators both in vivo and in vitro upon caspase-8 inhibition.

### The kinase activity of RIPK1 is involved in IETD-induced neutrophil activation

**Figure 5:**
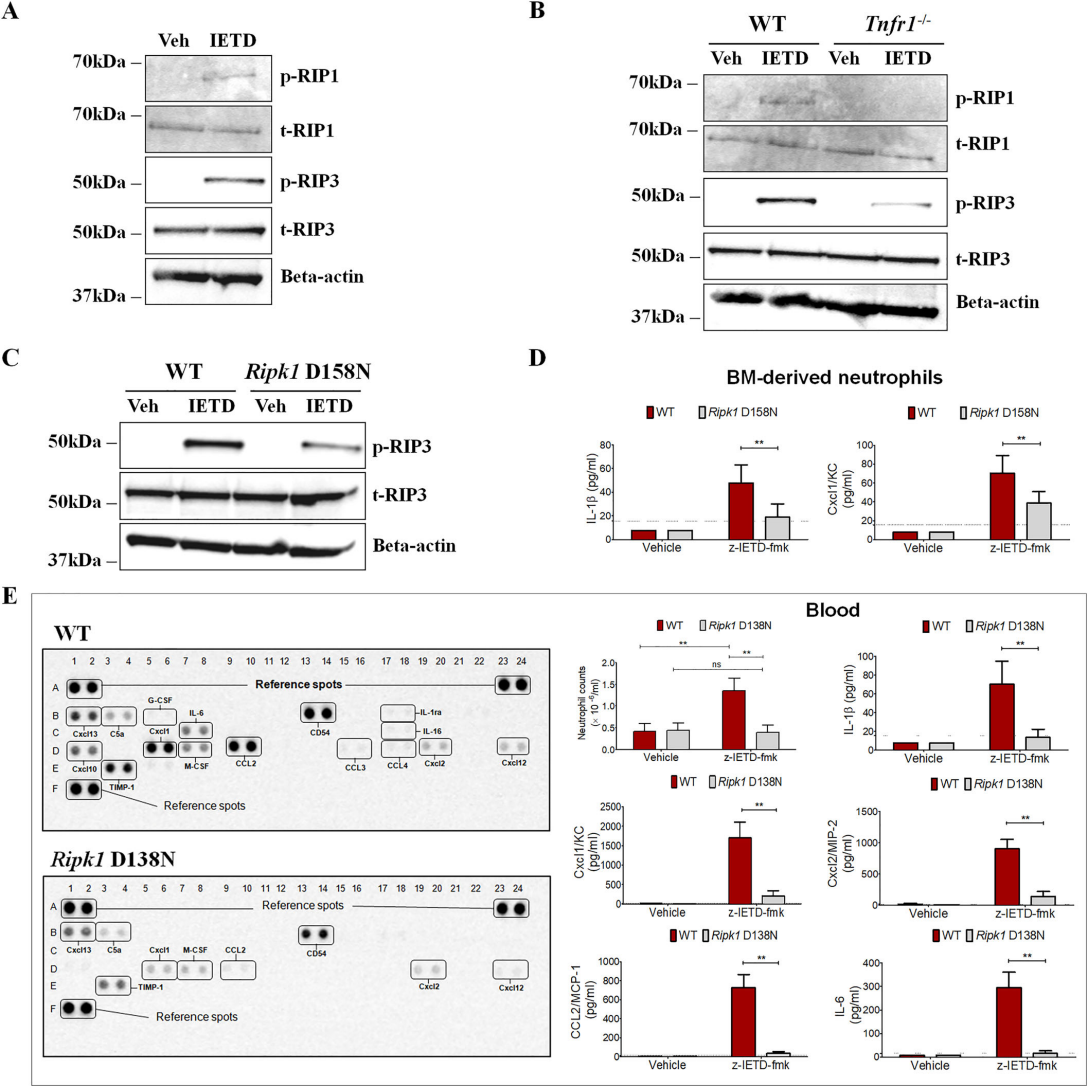
The kinase activity of RIPK1 promotes RIPK3 phosphorylation and cytokine release upon caspase-8 inhibition. (**A**, **B**) Caspase-8 inhibition induces TNF receptor-dependent RIPK1 and RIPK3 activation. Bone marrow-derived neutrophils from wild-type (WT) or type I TNF receptor-deficient (*Tnfr1*^−/−^) mice were treated with z-IETD-fmk (50 μM) or vehicle for 3 h. The activated forms of RIPK1 (pRIP1) and RIPK3 (pRIP3) were detected in cell lysates by western blot using phospho-specific antibodies. Total RIPK1 (t-RIP1) and RIPK3 (t-RIP3) were detected using antibodies directed against the respective full-length molecules. (**C**) Bone marrow neutrophils from mice with defective RIPK1 kinase activity (*Ripk1* D158N) or control wild-type (WT) mice were treated with z-IETD-fmk (50 μM) or vehicle, and Western blot analysis was performed on cell lysates obtained at 3 h post-treatment. (**D**) Bone marrow neutrophils with defective RIPK1 kinase activity (*Ripk1* D158N) or control wild-type (WT) neutrophils were treated with z-IETD-fmk (50 μM) or vehicle, and cytokine concentrations were measured by ELISA in culture supernatants obtained 24 post-treatment. Data are means + SDs (*n* = 5 biological replicates). (**E**) Mice with defective RIPK1 kinase activity (*Ripk1* D158N) or control WT mice were treated with z-IETD-fmk (6 mg/kg i.p.). Blood was collected 4 h later for plasma cytokine array analysis (left panels) or measurement of neutrophil counts and plasma cytokine concentrations by ELISA (right panels). Columns and bars represent means +  SDs of five determinations, each conducted in a different animal. (**A**–**C**) β-actin blots, used as loading controls, derive from the same experiment and were processed in parallel. (**D**, **E**) **P < 0.01 by the Mann–Whitney test. ns non-significant. Source data are available online for this figure.

**Figure EV4:**
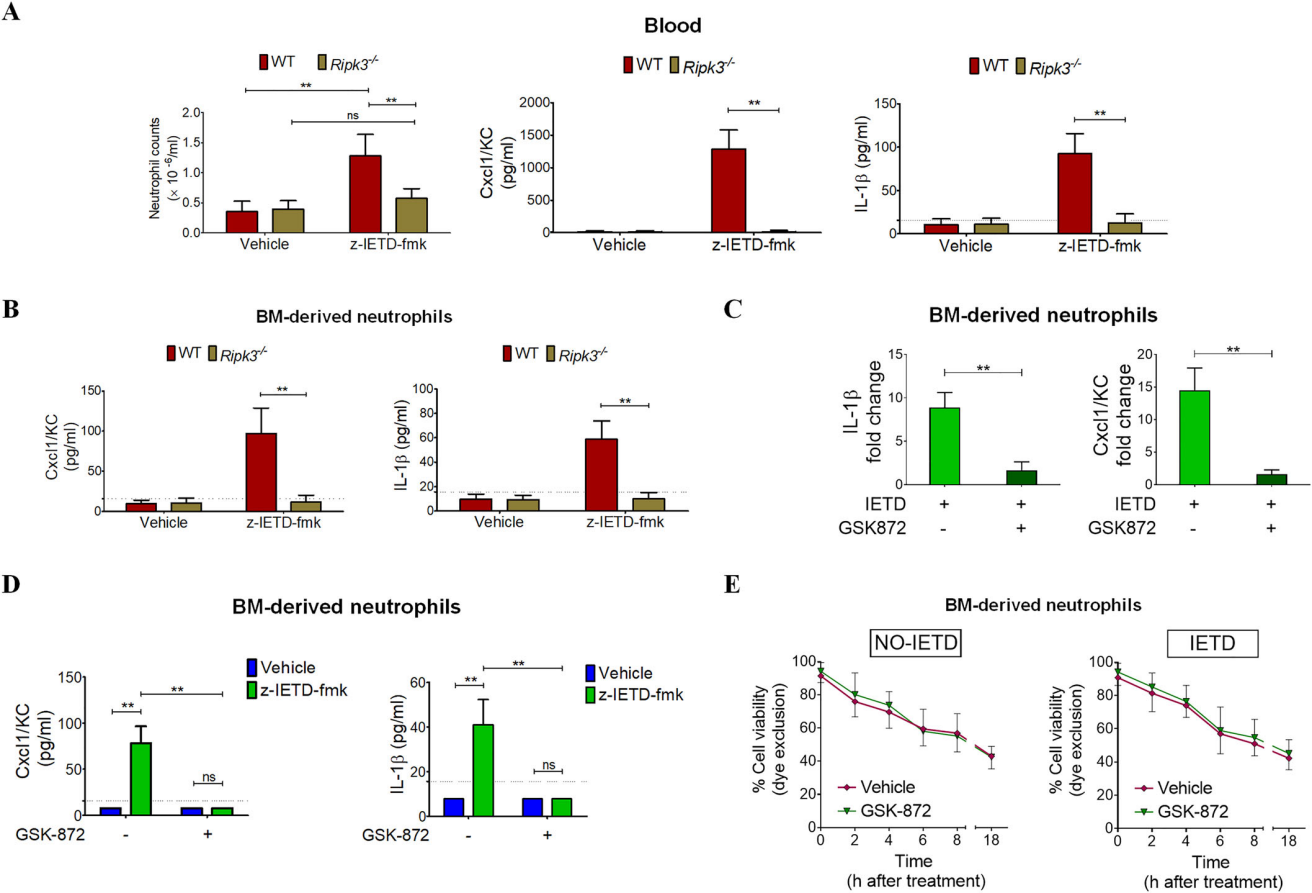
IETD-induced neutrophilia and cytokine release require the kinase activity of RIPK3 (related to Fig. 5). (**A**) Mice lacking RIPK3 (Ripk3^−/−^) or control wild-type (WT) mice were treated with z-IETD (6 mg/kg i.p.). Blood was collected at 4 h to measure neutrophil counts and plasma cytokine levels by ELISA. (**B**) Bone marrow-derived neutrophils from Ripk3^−/−^ or WT mice were treated overnight with z-IETD-fmk (50 μM) or vehicle. Cytokine concentrations were measured in the culture supernatants. (**C**) Bone marrow-derived neutrophils were pretreated for 1 h with the RIPK3 inhibitor GSK-872 (2 μΜ) or its vehicle before exposure to z-IETD-fmk (50 μM). Cells were collected for RNA extraction and quantitative real-time PCR analysis of pro-IL1β and Cxcl1 gene transcripts (left and right panels, respectively) at 3 h after exposure to z-IETD-fmk. (**D**) Bone marrow-derived neutrophils were pretreated for 1 h with the RIPK3 inhibitor GSK-872 (2 μΜ) or its vehicle before exposure to z-IETD-fmk (50 μM). After 24 h, cytokine concentrations were measured in the culture supernatants. (**E**) Bone marrow-derived neutrophils were pretreated for 1 h with the RIPK3 inhibitor GSK-872 (2 μΜ) or its vehicle before exposure to z-IETD-fmk (50 μM) and collected at the indicated times for determination of viability. (**A**) Columns and bars represent the mean ± SDs of five determinations, each performed using samples from different animals. (**B**, **C**) Columns bars represent the mean + SDs (*n* = 5 biological replicates). (**D**) Points and bars represent the mean + SDs (*n* = 5 biological replicates). (**A**–**D**) **P < 0.01 by the Mann–Whitney test. ns non-significant. Source data are available online for this figure.

Since TNFR1 activation can recruit RIPK1 to the receptor, we next examined the role of this kinase. Treatment with IETD resulted in phosphorylation of RIPK1 and RIPK3, a downstream kinase, as assessed by western blot analysis using phospho-specific antibodies (Fig. 5A). Notably, RIPK1 phosphorylation was completely abrogated, and RIPK3 phosphorylation was largely (> 50%) reduced, in IETD-stimulated *Tnfr1*^−/−^ neutrophils, suggesting that activation of these kinases is downstream of TNFR1 signaling (Fig. 5B). To analyze the functional consequences of RIPK1 activation, in further experiments we used *Ripk1*^*D138N/D138N*^ (RIPK1 kinase dead or RIPK1-KD) mice, which carry a single amino acid substitution that results in defective RIPK1 autophosphorylation/activation. Western blot analysis revealed that RIPK1-KD isolated neutrophils showed reduced (> 50%) RIPK3 phosphorylation upon caspase-8 inhibition (Fig. 5C). Furthermore, these cells were unable to produce IL-1β in response to IETD, while production of Cxcl1 was partially but significantly inhibited (Fig. 5D). Accordingly, nearly all of the cytokine elevations observed in wild-type mice (including elevations in circulating levels of IL-1β, IL-1ra, CXCL10, IL-6, IL-16, CCL3, and CCL4) were abrogated in IETD-inoculated RIPK1-KD mice. The remaining cytokines (such as Cxcl1/2/12/13, CCL2, and M-CSF, TIMP1) showed partially decreased levels (Fig. 5E). Notably, cytokine responses to IETD were completely abrogated in the absence of RIPK3 both in vivo and in vitro (Fig. EV4A,B). Moreover, the RIPK3 inhibitor GSK-872 completely abolished IETD-induced upregulation of *IL-1β* and *Cxcl1* mRNA and the production of these cytokines (Fig. EV4C,D). The absence of cell death under these conditions (Fig. EV4E) excluded the possibility that the observed reduction in gene expression resulted from caspase-8-dependent apoptosis, which can occur upon exposure to GSK-872 (Mandal et al, 2014). Thus, these findings indicate that the kinase activity of RIPK3, rather than its scaffold function, is essential for neutrophil responses to IETD stimulation. Collectively these data indicate that the kinase activities of RIPK1 and 3 are sequentially required for IETD-induced neutrophil activation. The slightly different phenotype of *Ripk3*^*−/−*^ *vs Ripk1*^*D138N/ D138N*^ mice (complete abrogation *vs* partial, albeit marked, reduction of cytokine responses, respectively) is likely due to low-grade, residual RIPK3 phosphorylation in the absence of RIPK1 kinase activity. The mechanisms responsible for this residual RIPK3 phosphorylation are currently under scrutiny.

### IETD-induced IL1-β release in neutrophils requires gasdermin D but not canonical inflammasome components

**Figure 6:**
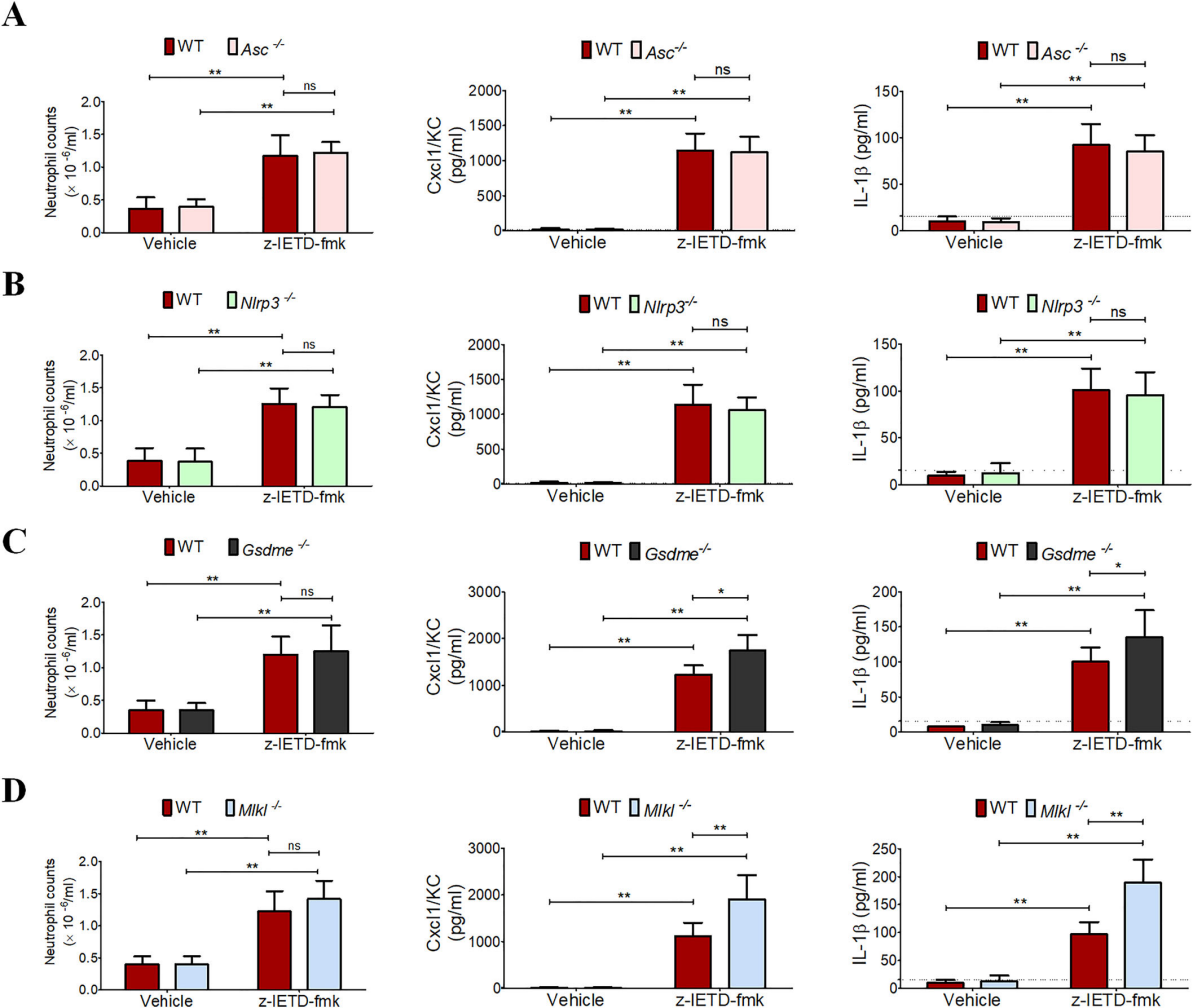
The canonical inflammasome components ASC and NLRP3, and the pore-forming molecules MLKL and GSDME, are dispensable for IL-1β release upon caspase-8 inhibition. (**A**–**D**) Mice lacking ASC (*Asc*^*−/−*^; **A**), NLRP3 (*Nlrp3*^*−/−*^; **B**), GSDME (*Gsdme*^*−/−*^; **C**), MLKL (*Mlkl*^*−/−*^; **D**), or control wild-type(WT) mice were treated with z-IETD-fmk (6 mg/kg i.p.) or vehicle. Blood was collected 4 h later to determine neutrophil counts and plasma cytokine concentrations. Columns and bars represent means + SDs of five determinations, each conducted in a different animal. (**A**–**D**) *P < 0.05; **P < 0.01 by the Mann–Whitney test. ns non-significant. Source data are available online for this figure.

**Figure EV5:**
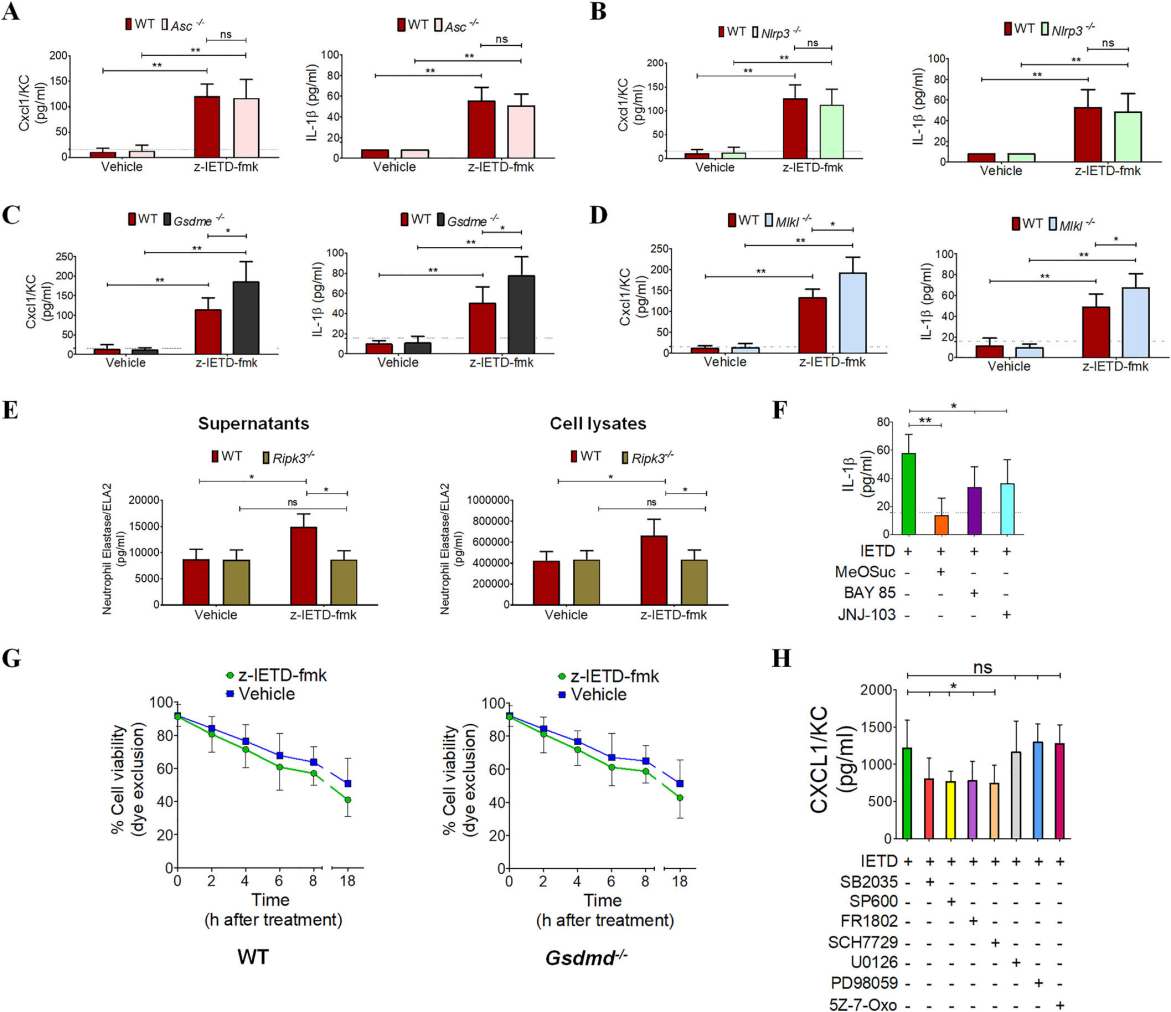
IETD-induced IL-1β release is independent of NLRP3, ASC, and cell death, but requires neutrophil serine protease activity (related to Figs. 6 and 7). Bone marrow-derived neutrophils were isolated from mice lacking ASC (*Asc*^−/−^; (**A**)), NLRP3 (*Nlrp3*^−/−^; (**B**)), GSDME (*Gsdme*^−/−^; (**C**)), MLKL (*Mlkl*^−/−^; (**D**)), or from control wild-type (WT) mice. Neutrophils were cultured overnight in the presence of z-IETD-fmk or vehicle. CXCL1 and IL-1β levels were measured in culture supernatants by ELISA. (**E**) Effect of z-IETD-fmk (50 μM) on the expression of neutrophil elastase in supernatants and cell lysates of RIPK3^−/−^ and wild-type neutrophils. Cell cultures' supernatants and cell lysates were obtained at 4 h after stimulation. Neutrophil elastase levels were measured by ELISA. (**F**) Effects of neutrophil serine protease inhibitors on IETD-induced IL-1β release in bone marrow neutrophils. Neutrophils were pretreated with the broad neutrophil serine protease inhibitor MeOSuc-AAPV-CMK (MeOSuc), the specific elastase inhibitor BAY 85-8501 (BAY 85), or the cathepsin G inhibitor JNJ-10311795 (JNJ-103) for 2 h before the addition of z-IETD-fmk (50 μM) or vehicle. After overnight culture, IL-1β concentrations were measured in the culture supernatants. (**G**) Spontaneous cell death in bone marrow neutrophils cultured in vitro with z-IETD-fmk (50 μM) or DMSO vehicle. Cells were isolated from wild-type (WT) or GSDMD-deficient (*Gsdmd*^*−/−*^) mice. (**H**) Bone marrow-derived neutrophils were pretreated for 2 h with the indicated inhibitors of signaling kinases before the addition of z-IETD-fmk (50 μM) or vehicle. After overnight incubation, CXCL1 levels were measured by ELISA in culture supernatants. In all panels, data are means + SDs (*n* = 5 biological replicates). Source data are available online for this figure.

**Figure 7:**
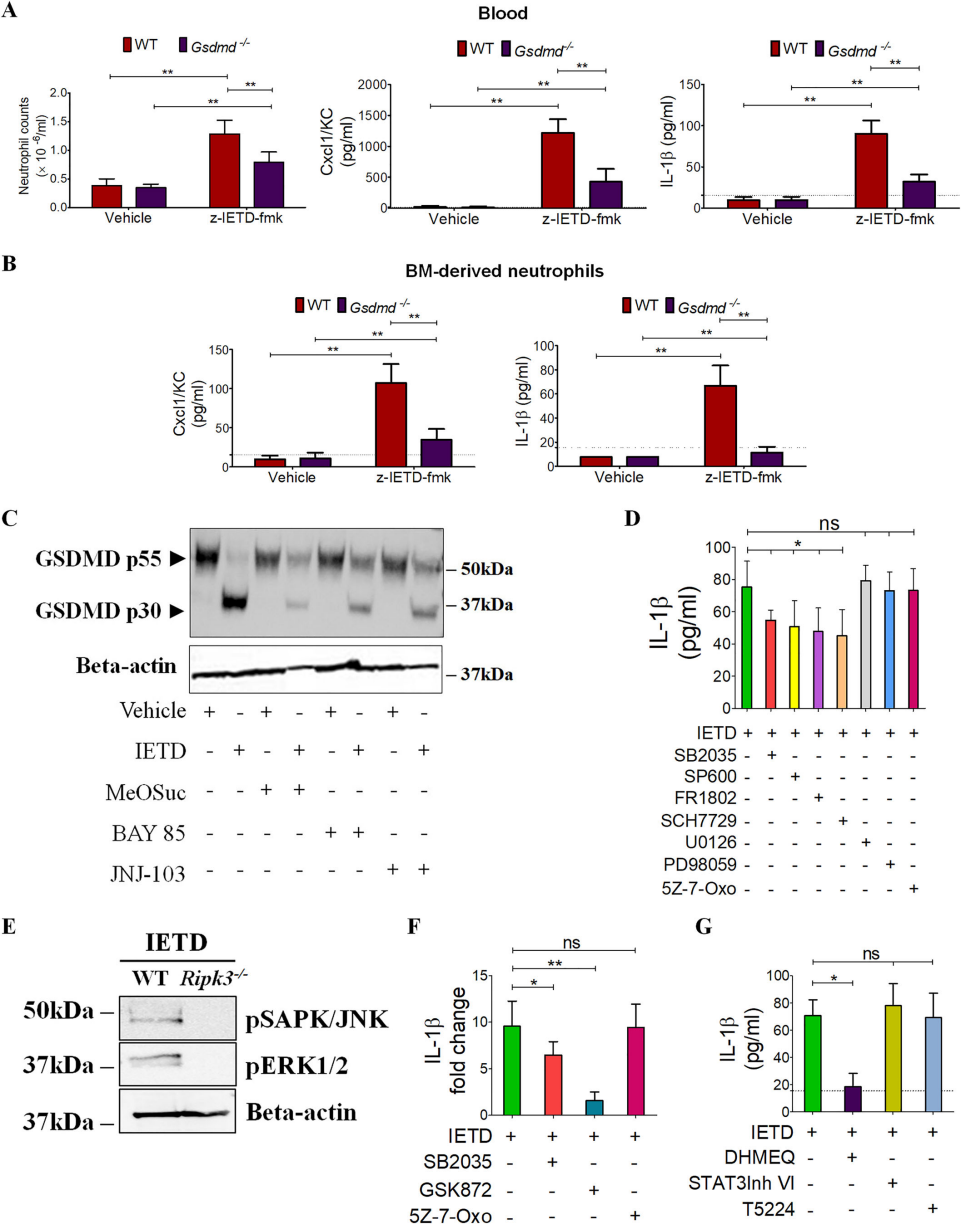
GSDMD is processed by neutrophil serine proteases and mediates IETD-induced IL-1β release. (**A**) Mice lacking GSDMD (*Gsdmd*^*−/−*^) or control wild-type (WT) mice were treated with z-IETD-fmk (6 mg/kg, i.p.). Blood was collected 4 h later to determine neutrophil counts and plasma cytokine concentrations. Columns and bars represent means + SD of five determinations, each conducted in a different animal. (**B**) Bone marrow-derived neutrophils from *Gsdmd*^*−/−*^or WT mice were treated overnight with z-IETD-fmk, and cytokine concentrations were measured in the culture supernatants. (**C**) Effect of neutrophil serine protease inhibitors on GSDMD processing. Bone marrow-derived neutrophils were pretreated with the broad neutrophil serine protease inhibitor MeOSuc-AAPV-CMK (MeOSuc), the specific elastase inhibitor BAY 85-8501 (BAY 85), or the cathepsin G inhibitor JNJ-10311795 (JNJ-103) for 2 h before the addition of z-IETD-fmk (50 μM) or vehicle. Neutrophil lysates were collected 3 h after z-IETD-fmk treatment and processed for western blot analysis using anti-GSDMD antibodies. (**D**) Bone marrow-derived neutrophils were pretreated for 2 h with the indicated inhibitors of signaling kinases before the addition of z-IETD-fmk (50 μM) or vehicle. After overnight incubation, IL-1β levels were measured by ELISA in culture supernatants. (**E**) Bone marrow-derived neutrophils from mice lacking RIPK3 (*Ripk3*^*−/−*^) or control WT mice were treated with z-IETD-fmk (50 μM) or vehicle. Western blot analysis was performed on cell lysates obtained 2 h after z-IETD-fmk addition, using phospho-specific antibodies directed against the activated forms of JNK (pSAPK/JNK) or ERK1/2 (pERK1/2) MAPKs. (**F**) Relative mRNA expression levels of IL-1β in bone marrow-derived neutrophils pretreated with p38MAPK inhibitor (SB2035), RIPK3 inhibitor (GSK872), or TAK1 inhibitor (5Z-7-Oxo) for 2 h and then exposed to z-IETD-fmk (50 μM) or vehicle for 3 h. (**G**) Bone marrow-derived neutrophils were pretreated for 2 h with the indicated transcription factor inhibitors before the addition of z-IETD-fmk (50 μM) or vehicle. After overnight incubation, IL-1β levels were measured by ELISA in culture supernatants. (**B**, **D**, **F**, **G**) Data are means + SDs (*n* = 5 biological replicates). (**C**, **E**) β-actin blots, used as loading controls, derive from the same experiment and were processed in parallel. (**A**, **B**, **D**, **F**, **G**) *P < 0.05; **P < 0.01 by the Mann–Whitney test. ns non-significant. Source data are available online for this figure.

In most cell types, IL-1β release requires assembly of the inflammasome, a large multiprotein complex that contains activated caspases. These caspases mediate the processing of pro-IL1-β and activate pore-forming proteins, such as gasdermins (GSDMs). Given the ability of pharmacological caspase-8 inhibition to cause the release of IL-1β both in vivo and in vitro, we next investigated the role of canonical inflammasome components such as NLRP3 and ASC using KO mice lacking these molecules. First, we found that *Asc*^−/−^ mice are indistinguishable from wild-type controls in their ability to produce mature IL-1β or other cytokines after IETD administration, either in vivo or in vitro (Figs. 6A and EV5A). Similarly, neutrophils lacking the inflammasome sensor NLRP3, which is essential for inflammasome activation in response to a wide range of stimuli, did not differ from wild-type cells in their in vitro responses to IETD (Figs. 6B and EV5B). Collectively, these data indicate that IETD-induced IL-1β release is largely independent of canonical inflammasome components, such as ASC and NLRP3. Efficient extracellular release of ΙL-1β requires pore-forming proteins, such as gasdermin D (GSDMD) or gasdermin E (GSDME), which are activated by the proteolytic activity of caspases following inflammasome assembly. In addition to caspases, serine proteases—abundantly present in primary granules—can also activate gasdermins in neutrophils (Yow et al, 2022). MLKL, another pore-forming protein, has been shown to facilitate the release of intracellular contents in granulocytes (D’Cruz et al, 2018). Therefore, we tested mice lacking GSDMD, GSDME, or MLKL for their ability to release IL-1β after treatment with the z-IETD-fmk caspase-8 inhibitor. While GSDME and MLKL were totally dispensable for in vivo responses to IETD administration (Fig. 6C,D), IL-1β levels and neutrophilia were severely reduced in mice lacking GSDMD (Fig. 7A). Accordingly, neutrophils lacking GSDME or MLKL produced significantly higher amounts of the cytokine when exposed to z-IETD-fmk (Fig. EV5C,D), while the opposite was observed in neutrophils lacking GSDMD, which exhibited significantly reduced IL-1β levels compared to wild-type cells (Fig. 7B).

In addition, GSDMD processing was detected by western blot in lysates from wild-type neutrophils exposed to IETD (Fig. 7C). We therefore hypothesized that GSDMD processing might result from increased cleavage by serine proteases, such as neutrophil elastase and cathepsin G, whose genes (along with those encoding other granule proteins) were significantly upregulated by IETD in our previous RNA-sequencing experiments (Fig. EV2C). We first found that neutrophil elastase was upregulated also at the protein level and in a RIPK3-dependent manner in both cell lysates and supernatants from IETD-treated neutrophils (Fig. EV5E). Supporting a role for serine proteases in IETD-induced GSDMD activation, pretreatment with MeOSuc-AAPV-CMK, a broad neutrophil serine protease inhibitor, largely prevented GSDMD processing (Fig. 7C) as well as IL-1β release (Fig. EV5F). Moreover, the specific elastase inhibitor BAY 85-8501 and the cathepsin G inhibitor JNJ-10311795 each partially inhibited GSDMD processing (Fig. 7C) and IL-1β release (Fig. EV5F). Since GSDMD activation can lead to pyroptosis, a form of programmed cell death, we assessed neutrophil viability for up to 24 h following IETD treatment. Despite GSDMD activation, no differences in cell death were observed between GSDMD-deficient and wild-type neutrophils upon IETD treatment (Fig. EV5G), indicating that, under these conditions, GSDMD activation does not induce pyroptosis. These findings are consistent with the notion that GSDMD activation does not necessarily trigger pyroptosis and instead can be required for IL-1β release from viable cells (Devant and Kagan, 2023; Evavold et al, 2018). Collectively, these data indicate that elastase and cathepsin G, both stored in primary granules, cooperate in the processing and activation of GSDMD upon caspase-8 inhibition in neutrophils, thereby promoting IL-1β release without requiring pyroptosis or ASC/NLRP3-dependent inflammasomes.

### IETD-induced cytokine production depends on NF-κB and MAPK activation

Next, we focused on the mechanisms leading to cytokine induction downstream of RIPK3 by using selective inhibitors of signaling molecules and transcription factors. Previous studies have shown that RIPK1/3 activation can induce cytokine production in macrophages through the ERK mitogen-activated protein kinase (MAPK) and the NF-κB transcription factor (Najjar et al, 2016). Therefore, we tested MAPK and NF-κB small molecule inhibitors for their ability to attenuate IETD-induced cytokine production. In vivo, IL-1β and Cxcl1 responses to IETD were reduced by pre-treating mice with direct inhibitors of p38 (SB203580), JNK (SP600125), or ERK1/2 (FR180204 or SCH772984; Figs. 7D and EV5H). In contrast, indirect ERK inhibitors, such as U0126or PD98059 that act on the upstream MEK1/2 kinases, showed little or no effect, suggesting that ERK activation in this context does not involve the classical MAPK activation pathway typically triggered by growth factors. RIPK1 can also function as a scaffold to activate proinflammatory signaling via the TAK1 kinase that, in turn, activates MAPK and NF-κB (Newton, 2020). However, the Tak1-specific inhibitor 5Z-7-oxozeaenol did not inhibit IETD-induced in vivo cytokine production (Figs. 7D and EV5H). Moreover, in agreement with the reduction in cytokine levels previously observed in vivo by treatment with MAPK inhibitors, exposure to IETD resulted in RIPK3-dependent p38, JNK, and ERK1/2 phosphorylation in bone marrow neutrophils, as detected by western blot (Fig. 7E). Furthermore, *il1b* gene activation was prevented by pretreatment with MAPK or RIPK3 inhibitors, but not with a TAK inhibitor (Fig. 7F). We next focused on the role of the NF-κB, STAT3, and AP-1 transcription factors. Inhibition of NF-κB using DHMEQ significantly reduced IL-1β responses to IETD in isolated neutrophils, while no effects were observed upon inhibition of the STAT3 or AP-1 transcription factors (Fig. 7G). Collectively, these data indicate that the MAPK kinases p38, ERK1/2, and JNK, as well as NF-κB, are activated downstream of RIPK3, but not TAK1, to induce *il1b* gene activation and IL-1β release in IETD-stimulated neutrophils.

## Discussion

The enzymatic activity of caspase-8 is essential for suppressing RIPK1 hyperactivation during homeostasis, and chronic defects in this regulatory function result in tissue damage and uncontrolled inflammation (Schwarzer et al, 2020; Varfolomeev et al, 1998; Weinlich et al, 2013). Since many of these catastrophic events are rescued by co-deletion of RIPK3 or MLKL, all pathological changes associated with defective caspase-8 function have been traditionally attributed to necroptosis resulting from the sequential activation of RIPK1, RIPK3, and MLKL. However, it is now well-established that activated RIPK1 and/or RIPK3 can also induce inflammatory damage by promoting apoptosis or pyroptosis, in addition to necroptosis (Fritsch et al, 2019; Newton et al, 2024). Whether and by which mechanisms defective caspase-8 function can trigger inflammation in the absence of cell death during homeostasis remains less clear. In vivo, we find here that acute inhibition of caspase-8 activity results in marked inflammatory changes, as evidenced by profound neutrophilia and elevated circulating cytokine levels, without evidence of necroptosis, pyroptosis, or other forms of cell death. These responses are not associated with clinical signs or evidence of tissue damage, do not affect the skin or the intestines (which are the primary target organs of chronic caspase-8 deficiency), and are not due to off-target effects of the caspase-8 inhibitor or to other non-specific events, since they are abrogated in the absence of caspase-8. Collectively, our findings reveal an unexpected, systemic, and constitutively activated proinflammatory pathway that can be potently unleashed in vivo by pharmacological caspase-8 inactivation.

Previous studies have shown that pharmacological inhibition of caspase-8 can produce proinflammatory cytokine elevations in vivo, although the underlying molecular mechanisms have only been partially explored (Lentini et al, 2023). Here, we find that caspase-8 inhibition triggers a massive transcriptional program in neutrophils affecting over one-fourth of the entire genome. This response is strictly dependent on caspase-8 and is not due to off-target effects of the inhibitor. Moreover, under identical conditions, macrophages show virtually no transcriptional response. This striking cell-type specificity aligns with the notion that caspase-8 functions are dictated by the intracellular context, including upstream signaling pathways and the availability of alternative binding partners (Newton, 2020; Orning and Lien, 2021). Caspase-8 inhibition induces a unique gene expression landscape in neutrophils, marked by the prominent upregulation of innate immunity receptors and co-receptors, adhesion and migration molecules, survival factors, granule proteins, and cytokines and their receptors, while several factors involved in neutrophil extracellular trap (or NET) formation are moderately downregulated. Notably, the upregulated genes include those typically expressed at both early (e.g., granule proteins) and late (e.g., *Il1b*, *Csf3r*, *Ccl6*, *Fxyd5*) stages of neutrophil differentiation and aging (Grieshaber-Bouyer et al, 2021). Most of the downregulated genes were associated with DNA synthesis pathways, which may seem surprising for short-lived, terminally differentiated cells like neutrophils. However, recent studies suggest that neutrophils retain DNA replication machinery, which plays a role in repairing oxidative DNA damage caused by both spontaneous and induced reactive oxygen species (Azzouz and Palaniyar, 2022; Harbort et al, 2015).

Although caspase-8 inhibition induced the production of a broad array of proinflammatory cytokines, it did not increase TNF-α release in our studies. However, blockade of TNF-α or deletion of *Tnfr1* prevented inhibitor-induced RIPK1/3 phosphorylation and abolished cytokine elevations both in vivo and in vitro. In addition, we show that bone marrow-derived neutrophils produce small but detectable amounts of TNF-α in the absence of external stimuli, similar to recent observations in macrophages (Luecke et al, 2023). Taken together, these findings suggest that tonic TNF-α production initiates the caspase-8-controlled pathway by promoting the sequential phosphorylation of RIPK1 and RIPK3. Much like type I interferon, tonic TNF-α production is essential for hematopoiesis (Espin-Palazon et al, 2014; Gogoleva et al, 2021; Gough et al, 2012). While TNF-α is not essential for embryonic development in mice, it is released in a specific spatiotemporal pattern during gestation. Failure of caspase-8 to prevent TNF-α-dependent necroptosis of yolk sac endothelial cells by cleaving RIPK1 leads to embryonic lethality (Dillon et al, 2014; Newton et al, 2024; Zhang et al, 2021). Postnatally, TNF-α can trigger necroptosis and inflammation in skin and intestinal epithelia under conditions of IKK-NF-κB or caspase-8 deficiency (Newton, 2020; Orning and Lien, 2021). However, it remains unclear whether TNF-α is produced spontaneously or in response to commensal organisms in these latter contexts. Collectively, our findings suggest that neutrophils, being highly abundant, may serve as a significant source of tonic TNF-α and, as such, could play an important role in its effects under both homeostatic conditions and in the context of impaired caspase-8 function.

We also demonstrate that RIPK3 kinase activity is absolutely required for the cytokine responses induced by caspase-8 inhibition. These findings support the idea that, under certain conditions, RIPK3 activation can drive cytokine production independently of MLKL and necroptosis. For example, RIPK3 was necessary for cytokine responses in macrophages activated with LPS in the presence of a pan-caspase inhibitor (Najjar et al, 2016), in LPS-stimulated dendritic cells (Moriwaki and Chan, 2016), and in virally infected brain cells (Daniels et al, 2017). Moreover, consistent with these studies, we find that RIPK3-dependent cytokine production requires the transcription factor NF-κB. However, our findings differ from previous work in that we demonstrate that specific caspase-8 inhibition alone is sufficient to trigger RIPK3-dependent cytokine production, whereas earlier studies required exogenous, pathogen-derived stimuli to induce cytokine responses. Under certain conditions, enzymatically inactive caspase-8 can promote inflammasome formation and IL-1β release through mechanisms involving the adapter ASC and caspase-1/11. For example, in the absence of MLKL, enzymatically inactive caspase-8 provides a nucleation signal for ASC-dependent inflammasome formation, leading to pyroptotic death of mouse intestinal epithelial cells and perinatal lethality (Newton et al, 2019). Moreover, in macrophages lacking inhibitor of apoptosis proteins (IAPs), LPS stimulation induces RIPK3- and NLRP3-dependent canonical inflammasome activation (Lawlor et al, 2015). However, we found no evidence that the proinflammatory effects of caspase-8 inhibition depend on the canonical inflammasome in our experimental system. Both ASC and NLRP3 were dispensable for IL-1β release and neutrophilia induction upon treatment with the IETD caspase-8 inhibitor. Instead, IL-1β release was primarily dependent on GSDMD activation and processing, which were largely mediated by the cooperative activity of elastase and cathepsin-G, two serine proteases found in neutrophil granules. These findings align with the notion that GSDMD can be cleaved and activated not only by caspase-1/11 but also by serine proteases (Burgener et al, 2019; Chen et al, 2018; Kambara et al, 2018; Sollberger et al, 2018). Collectively, our data suggest that upon acute caspase-8 inhibition, neutrophils release IL-1β via a serine protease- and GSDMD-dependent mechanism that is largely independent of canonical inflammasome components. Furthermore, neutrophils appear to be the primary source of in vivo IL-1β production under these conditions. Regarding other cytokines, our data suggest that cell types other than neutrophils may also be involved. However, cultured macrophages did not show upregulation of cytokine genes in response to caspase-8 inhibition. Future investigations will focus on identifying additional cell types that may be activated in vivo upon caspase-8 blockade.

In conclusion, we found that caspase-8 controls a neutrophil-centric pathway, sequentially activated by autocrine TNF-α production, RIPK1/3 phosphorylation, and NF-κB- and MAPK-dependent activation of proinflammatory and chemotactic cytokine genes. Although IETD-induced caspase-8 inhibition did not increase cell death in neutrophil cultures at the population level, further observations involving single-cell analyses will be required to more precisely assess the relationship between caspase-8–regulated transcriptional activity and activation of cell death pathways. Notably, the caspase-8-controlled pathway described here involves IL-1β release through GSDMD activation by neutrophil serine proteases. Activation of this pathway using a pharmacological caspase-8 inhibitor enhanced host defenses in mice subjected to cecal ligation and puncture, a well-characterized model of lethal bacterial sepsis. Nonetheless, the therapeutic use of caspase-8 inhibitors in human sepsis will require validation in larger animal models and the development of less toxic compounds, as only a few interventions that were effective in mice have to date been translated into clinical efficacy in septic patients.

## Methods

**Table:** Reagents and tools table

Reagent/resource	Reference or source	Identifier or catalog number
**Experimental models**		
Mouse: C57BL/6	Charles River Laboratories	Strain Code 027
Mouse: *Tnfr1*^*−/−*^	Jackson Laboratory, Bar Harbor, Maine, USA	Strain #002818
Mouse: *Ripk3*^*−/−*^	Genentech, Inc., CA, USA	N/A
Mouse: *Ripk1* D138N	University of Cologne, Germany	N/A
Mouse: *Csf3r*^*−/−*^	Jackson Laboratory, Bar Harbor, Maine, USA	Strain #017838
Mouse: *Mlkl*^*−/−*^	The Walter and Eliza Hall Institute of Medical Research, Australia	N/A
Mouse: *Mlkl*^*−/−*^*Casp8*^*−/−*^	University Health Network, Toronto, Ontario, Canada	N/A
Mouse: *Nlpr3*^*−/−*^	Genentech, Inc., CA, USA	N/A
Mouse: *Asc*^*−/−*^	Millennium Pharmaceuticals, Cambridge, MA	N/A
Mouse: *Gsdme*^*−/−*^	Jackson Laboratory, Bar Harbor, Maine, USA	Strain #032411
Mouse: *Gsdmd*^*−/−*^	Genentech, Inc., CA, USA	N/A
**Chemicals, enzymes, and other reagents**		
Z-IETD-FMK (Caspase-8 Inhibitor)	Sellckchem (Selleck Chemicals LLC)	Cat #S7314
Dimethyl Sulfoxide (DMSO)	Sigma-Aldrich	Cat #D8418
Dulbecco′s Phosphate Buffered Saline (PBS)	Sigma-Aldrich	Cat #D8537
Dulbecco′s Phosphate Buffered Saline 10X	Sigma-Aldrich	Cat #D1408
Corning® Cell Culture Grade Water	Corning	Cat #25-055-CV
RPMI 1640 Medium	Sigma-Aldrich	Cat #R8758
Fetal Bovine Serum (FBS)	Sigma-Aldrich	Cat #F7524
Penicillin Streptomycin Solution 100X	Corning	Cat #30-002-Cl
Distilled Water Sterile Tissue Culture Tested	EuroClone	Cat #ECM0970L
Dulbecco’s Phosphate Buffer Saline w/o Calcium w/o Magnesium (DPBS)	EuroClone	Cat #ECB4004L
Percoll PLUS	GE Healthcare	Cod #17-5445-02
Recombinant Murine Macrophage Colony-Stimulating Factor (M-CSF)	PeproTech	Cat #315-02
RIPA Buffer (10×)	Cell Signaling Technology	Cat #9806
Phenylmethylsulfonyl fluoride (PMSF) protease inhibitor	Thermo Scientific	Cat #36978
Diisopropylfluorophosphate (DFP)	Sigma-Aldrich	Cat #D0879
AEBSF Ready Made Solution	Sigma-Aldrich	Cat #SBR00015
Red Blood Cell Lysis Buffer	Roche	REF #11814389001
4X Bolt™ LDS Sample Buffer	Invitrogen	Cat #B0007
10X Bolt Sample Reducing Agent	Invitrogen	Cat #B0009
Bovine Serum Albumin (BSA)	Sigma-Aldrich	Cat #A7906
Immobilon Forte Western HRP substrate	Millipore	WBLUF0100
3,3′,5,5′-Tetramethylbenzidine (TMB) Liquid Substrate System for ELISA	Sigma-Aldrich	Cat #T0440
Recombinant Mouse TNF‑α	R&D Systems	Cat #410-MT
TRIzol™ Reagent	Invitrogen	Cat #15596018
TaqMan™ Gene Expression Assay (FAM) Inventoried Mouse ACTB (Actin, Beta) Endogenous Control	Applied Biosystems™	Cat #4352341E
TaqMan™ Gene Expression Assay (FAM) Inventoried IL-1beta Mm00434228_m1	Applied Biosystems™	Cat #4331182
TaqMan™ Gene Expression Assay (FAM) Inventoried Cxcl1 Mm04207460_m1	Applied Biosystems™	Cat #4331182
TaqMan™ Gene Expression Assay (FAM) Inventoried Kcnj4 Mm02027786_s1	Applied Biosystems™	Cat #4331182
TaqMan™ Gene Expression Assay (FAM) Inventoried Zbp1 Mm01247052_m1	Applied Biosystems™	Cat #4331182
TaqMan™ Gene Expression Assay (FAM) Inventoried Folr1 Mm00433355_m1	Applied Biosystems™	Cat #4331182
TaqMan™ Gene Expression Assay (FAM) Inventoried Ifn-1beta Mm00439552_s1	Applied Biosystems™	Cat #4331182
Etanercept	MedChemExpress	Cat #HY-108847
5Z-7-Oxozeaenol	MedChemExpress	Cat #HY-12686
SB203580	MedChemExpress	Cat #HY-10256
PD98059	MedChemExpress	Cat #HY-12028
U0126-EtOH	MedChemExpress	Cat #HY-12031
FR180204	MedChemExpress	Cat #HY-12275
SCH772984	MedChemExpress	Cat #HY-50846
SP600125	MedChemExpress	Cat #HY-12041
T5224	MedChemExpress	Cat #HY-12270
MeOSuc-AAPV-CMK	MedChemExpress	Cat #HY-136888
JNJ-10311795	MedChemExpress	Cat #HY-122161
BAY-85-8501	MedChemExpress	Cat #HY-19908
GSK-872	MedChemExpress	Cat #HY-101872
DHMEQ	MedChemExpress	Cat #HY-14645
InSolution STAT3 Inhibitor VI	Calbiochem	Cat #501919-59-1
Purified Rat Anti-Mouse CD16/CD32 (Mouse BD Fc Block™) Clone 2.4G2 (RUO)	BD Biosciences	Cat #553142
CD16/CD32 Monoclonal Antibody (93), eBioscience™	Invitrogen	Cat #14-0161-82
PE Rat Anti-Mouse Ly-6G Clone 1A8	BD Biosciences	Cat #551461
PE Rat IgG2a, κ Isotype Control	BD Biosciences	Cat #553930
PE-CF594 Rat Anti-Mouse Ly-6G Clone 1A8 (RUO)	BD Biosciences	Cat #562700
F4/80 Monoclonal Antibody (BM8), eFluor™ 450, eBioscience™	Invitrogen	Cat #48-4801-82
Rat IgG2a kappa Isotype Control (eBR2a), eFluor™ 450, eBioscience™	Invitrogen	Cat #48-4321-82
PE/Cyanine7 anti-mouse F4/80 Antibody	Biolegend	Cat #123114
BV480 Rat Anti-CD11b Clone M1/70 (RUO)	BD Biosciences	Cat #566117
CD101 Monoclonal Antibody (Moushi101), APC, eBioscience™	Invitrogen	Cat #17-1011-82
CD24 Monoclonal Antibody (M1/69), APC-eFluor™ 780, eBioscience™	Invitrogen	Cat #47-0242-82
Alexa Fluor® 700 Hamster Anti-Mouse CD11c Clone HL3 (RUO)	BD Biosciences	Cat #560583
BUV737 Mouse Anti-Mouse CD182 (CXCR2) Clone 3F10_B3 (RUO)	BD Biosciences	Cat #752827
APC anti-mouse CD182 (CXCR2) Antibody Clone SA044G4 (RUO)	Biolegend	Cat #149312
BV605 Rat Anti-Mouse CD184 Clone 2B11/CXCR4 (RUO)	BD Biosciences	Cat #740378
PerCP/Cyanine5.5 anti-mouse CD184 (CXCR4) Antibody Clone L276F12 (RUO)	Biolegend	Cat #146510
BV421 Rat Anti-Mouse Ly-6C Clone AL-21 (RUO)	BD Biosciences	Cat #562727
BUV805 Rat Anti-Mouse CD45 Clone 30-F11 (RUO)	BD Biosciences	Cat #748370
Brilliant Violet 570™ anti-mouse CD62L Antibody (RUO)	Biolegend	Cat #104433
CD62L (L-Selectin) Monoclonal Antibody (MEL-14), eFluor™ 450, eBioscience	Invitrogen	Cat #48-0621-82
BV650 Mouse Anti-Mouse CD64 a and b Alloantigens Clone X54-5/7.1 (also known as X54-5/7.1) (RUO)	BD Biosciences	Cat #740622
BV711 Rat Anti-Mouse I-A/I-E Clone 2G9 (RUO)	BD Biosciences	Cat #743874
FITC Rat anti-Mouse CD172a (Sirpa) Clone P84 (RUO)	BD Biosciences	Cat #560316
PE anti-mouse CD54 Antibody (RUO)	Biolegend	Cat #116108
Ly-6G Monoclonal Antibody (1A8-Ly6g), Functional Grade, eBioscience™	Invitrogen	Cat #16-9668-82
Rat IgG2a kappa Isotype Control (eBR2a), Functional Grade, eBioscience™	Invitrogen	Cat #16-4321-82
Mouse CXCR2/IL-8RB Antibody	R&D Systems	Cat #MAB2164
Rat IgG2a Isotype Control	Invitrogen	Cat # 02-9688
Goat polyclonal to beta Actin- Loading Control antibody	Abcam	Cat #ab8229
Mouse IL-1 beta /IL-1F2 Antibody	R&D Systems	Cat #AF-401-NA
anti-Caspase-1 (p20) (mouse), mAb (Casper-1)	AdipoGen Life Sciences	Cat #AG-20B-0042-C100100
anti-Caspase-1 (p10) (mouse), mAb (Casper-2)	AdipoGen Life Sciences	Cat #AG-20B-0044-C100100
Phospho-RIP3 (Thr231/Ser232) (E7S1R) Rabbit mAb	Cell Signaling Technology	Cat #91702
RIP3 (D8J3L) Rabbit mAb	Cell Signaling Technology	Cat #15828
Phospho-RIP (Ser166) (E7G6O) RabbitmAb	Cell Signaling Technology	Cat #53286
RIP (D94C12) XP® Rabbit mAb	Cell Signaling Technology	Cat #3493
Anti-GSDMD antibody [EPR20859]	Abcam	Cat #ab219800
Polyclonal Swine Anti-Rabbit Immunoglobulins/HRP	Dako	Code number #P0399
Polyclonal Rabbit anti-Goat IgG HRP-conjugated Antibody	R&D Systems	Cat #HAF017
Mouse IgG Horseradish Peroxidase-conjugated Antibody	R&D Systems	Cat #HAF007
Mouse IL-1 beta/IL-1F2 DuoSet ELISA	R&D Systems	Cat #DY401
Mouse CXCL2/MIP-2 DuoSet ELISA	R&D Systems	Cat #DY452
Mouse CXCL1/KC DuoSet ELISA	R&D Systems	Cat #DY453
Mouse TNF-alpha DuoSet ELISA	R&D Systems	Cat #DY410
Mouse IL-18 DuoSet ELISA	R&D Systems	Cat #DY7625
Mouse IL-6 DuoSet ELISA	R&D Systems	Cat #DY406
Mouse CCL2/JE/MCP-1 DuoSet ELISA	R&D Systems	Cat #DY479
Mouse M-CSF DuoSet ELISA	R&D Systems	Cat #DY416
Mouse GM-CSF DuoSet ELISA	R&D Systems	Cat #DY415
Mouse Neutrophil Elastase/ELA2 DuoSet ELISA	R&D Systems	Cat #DY4517-05
Proteome Profiler Mouse Cytokine Array Kit, Panel A	R&D Systems	Cat #ARY006
Micro BCA™ Protein Assay Kit	ThermoFisher Scientific	Cat #23235
Pyrochrome® LAL Chromogenic Endotoxin Testing Reagent	Associates of Cape Cod, Inc.	Cat #C1500
LIVE/DEAD Fixable Aqua Dead Cell Stain Kit, for 405 nm excitation	Invitrogen	Cat #L34957
RNeasy Mini Kit	QIAGEN	Cat. No. / ID:74004
M-MLV Reverse Transcriptase	Invitrogen	Cat #28025013
Illumina Stranded Total RNA Prep Ribo-Zero Plus	Illumina	Cat ID #20040525
NextSeq 2000 P3 flow cell and reagents	Illumina	Cat ID #20040560
**Software**		
GrapdhPad Prism	GraphPad Software, Inc.	RRID:SCR_002798 (http://www.graphpad.com/)
Image Lab™ Software	Bio-Rad	Cat #1709690
i-control™Software for Infinite 200 PRO plate reader	TECAN	https://lifesciences.tecan.com/
CFX Maestro Software for CFX Real-Time PCR Instruments	Bio-Rad	Cat #12013758
FlowJo Software	TreeStar	https://www.flowjo.com/
ImageJ Software	National Institutes of Health and the Laboratory for Optical and Computational Instrumentation (LOCI, University of Wisconsin)	https://imagej.net/
Galaxy server, web-based platform	Center for Comparative Genomics and Bioinformatics at Penn State and the Department of Biology at Johns Hopkins University	https://usegalaxy.org/
ShinyGO 0.81 web-based platform	South Dakota State University (SDSU)	https://bioinformatics.sdstate.edu/go/
Heatmapper web-based platform	University of Alberta, Edmonton, Canada	http://www.heatmapper.ca/
**Other**		
BD Trucount™ Absolute Counting Tubes	BD Biosciences	Cat #340334
Precision Plus Protein™ WesternC™ Blotting Standard	Bio-Rad	Cat #1610376
Bolt 4 to 12%, Bis-Tris Mini Protein Gel	Invitrogen	Cat #NW04120B0X
20X Bolt MOPS SDS Running Buffer	Invitrogen	Cat #B0001
Immun-Blot PVDF Membrane	Bio-Rad	Cat #1620177
96 Well TC-Treated Microplates	Sigma-Aldrich	SKU CLS3599
96-well microplates with U-bottom, cellGrade™ BRANDplates®	VWR International	Cat #781960
Corning® Costar® TC-Treated Multiple Well Plates	Sigma-Aldrich	Cat #CLS3516
Corning®Costar® TC-Treated Multiple Well Plates	Sigma-Aldrich	Cat #CLS3512
Corning®Costar® TC-Treated Multiple Well Plates	Sigma-Aldrich	Cat #CLS3524
Nanodrop 2000 spectrophotometry	ThermoFisher Scientific	N/A
Bio-Rad Molecular Imager ChemiDoc™ XRS	Bio-Rad	Cat #1708265
Corning® Falcon® Cell Strainer 70 µm	Corning	Cat #CLS352350
Infinite® 200 PRO plate reader	TECAN	https://lifesciences.tecan.com/
Illumina NextSeq2000 instrument	Illumina	N/A
BD FACSCanto™ II Clinical Flow Cytometry System	BD Biosciences	N/A
FACSymphony Flow Cytometry System	BD Biosciences	N/A
CFX96 Touch Real-Time PCR Detection System	Bio-Rad	RRID:SCR_018064

### Mice

Six- to eight-week-old C57BL/6 wild-type (WT) female mice were obtained from Charles River Laboratories. Although data presented here were obtained with female mice only, sex-related differences in responses to caspase-8 inhibition or other treatments were not detected in additional experiments.*Csf3r*^*−/−*^ (Jackson Laboratory), *Mlkl*^*−/−*^ (Murphy et al, 2013), *Mlkl*^*−/−*^*Casp8*^*−/−*^ (Salmena et al, 2003), *Tnfr1*^*−/−*^ (Yang et al, 2010) (Jackson Laboratory), *Ripk1D158N* (Polykratis et al, 2014), *Ripk*3^*−/−*^ (Newton et al, 2004), *Asc*^*−/−*^ (Millennium Pharmaceuticals), *Nlrp3*^*−/−*^ (Costa et al, 2012)*, Gsdme*^*−/−*^ (Jackson Laboratory) and *Gsdmd*^*−/−*^ (Kayagaki et al, 2015) mice were all on a C57Bl/6 background. All mice were housed in individually ventilated cages under specific pathogen-free conditions in the animal facilities of the Department of Pathology of the University of Messina. All studies were performed in strict accordance with international guidelines for the use of laboratory animals and were approved by the relevant national authority (Ministero della Salute of Italy, permits no. 112/2023-PR and no. 1024/2024-PR). All efforts were made to minimize the number of animals used and their suffering.

### In vivo caspase-8 inhibition

z-IETD-fmk (a caspase-8 inhibitor) was dissolved in dimethyl sulfoxide (DMSO) to a 25 mM concentration and given to mice i.p. at a dose of 6 mg/kg of body weight. This dose was selected based on previous studies showing that it was able to induce, in vivo, transcriptionally regulated inflammatory changes by specifically inhibiting caspase-8 while not inducing off-target effects (Lentini et al, 2023). Endotoxin contamination in z-IETD-fmk preparations was measured using the Pyrochrome amebocyte lysate test. In selected experiments, mice were pretreated with etanercept (TNF inhibitor, 6 mg/kg, s.c.) or with GSK-872 (RIPK3 inhibitor, 25 mg/kg, i.p.) 24 h before z-IETD-fmk or vehicle administration (i.p.). In CXCR2 receptor neutralization experiments, WT and *Csf3r*^*−/−*^ mice were injected i.p. with anti-CXCR2 mAb (rat IgG_2A_) or rat IgG_2A_ isotype control (both 6 mg/kg) 24 h before z-IETD-fmk or vehicle administration (i.p.). After IETD treatment, blood, peritoneal lavage fluid, bone marrow, and spleen were obtained and analyzed for cell counts and cytokine determinations as previously described (Fama et al, 2020; Lentini et al, 2020). Briefly, blood was collected from the submandibular (facial) vein into 1.5 mL tubes containing 10% sodium heparin. Mice were euthanized via cervical dislocation and immediately dissected to obtain the spleen, the femurs, and the tibias. The spleen was cleaned of any attached fat and lymph nodes and minced into small pieces (~ 0.2 cm^2^) in sterile 35 mm culture dishes. The dissociated splenic and bone marrow tissues were then passed through a 70-µm cell strainer to obtain single-cell suspensions. Cell viability was measured by live/dead cell counting using the LIVE/DEAD Fixable Aqua Dead Cell Stain Kit.

### Cecal ligation and puncture

Cecal ligation and puncture was performed as previously described (Rittirsch et al, 2009). Briefly, 6- to 8-week-old female C57BL/6 mice were anesthetized with an i.p. injection of tiletamine and zolazepam (35 mg/kg each), combined with xylazine (15 mg/kg). After shaving and disinfecting the area, a midline incision approximately 1.5 cm in length was made to expose the cecum, which was then ligated with a 4.0 suture thread at a point roughly two-thirds of the distance between the distal pole and the ileal valve. A through-and-through puncture was performed in a mesenteric-to-antimesenteric direction using a 22-gauge needle. The cecum was gently squeezed to ensure patency by allowing a small drop of fecal material to extrude. After repositioning the cecum within the abdominal cavity, the peritoneal and muscle layers were sutured, and the skin was closed with metallic clips. Fluid resuscitation was provided via subcutaneous (s.c.) injection of 50 μl/g of prewarmed normal saline, and postoperative analgesia was administered with tramadol (20 mg/kg) s.c. every 6 h. A control group of sham-operated animals underwent the same procedure, excluding the ligation and puncture steps. Either z-IETD-fmk (6 mg/kg) or vehicle was administered i.p. at 1 h post-cecal puncture. After the challenge, animals were monitored for the onset of clinical symptoms. Disease severity was evaluated using a scoring system based on pre-determined clinical criteria and humane endpoints (Huet et al, 2013). Animals showing signs of irreversible disease were humanely euthanized. In some groups of mice, peritoneal lavage fluid, blood, and organ homogenates were collected and analyzed to determine CFU counts, cell numbers, and cytokine levels. To obtain peritoneal lavage samples, 2 ml of buffered saline was injected into the peritoneal cavity, and 1.7–1.9 ml of fluid was aspirated. Unconcentrated samples were used to measure cytokine levels.

### In vitro cell stimulation

Bone marrow-derived neutrophils were obtained from the femurs and tibias of 6–8-week-old female mice as previously described (Lentini et al, 2022; Lentini et al, 2021). Purity of neutrophil preparations was >95%, as assessed by flow cytometry. For in vitro stimulation experiments, cells were seeded in 96-well microtiter plates at a concentration of 5 × 10^5^ per well in 0.2 ml of RPMI medium with 10% fetal bovine serum. When indicated, neutrophils were pretreated with the TNF-α antagonist etanercept (1 µg/ml) or with increasing doses of mouse recombinant TNF-α (10, 25, and 50 ng/ml) at 2 h before the addition of z-IETD-fmk (50 µM) or vehicle. Cells were then cultured for the indicated length of time in the presence of z-IETD-fmk or vehicle. For the inhibitor studies, neutrophils were pretreated for 2 h in the presence or absence of intracellular kinase inhibitors: 5Z-7-Oxozeaenol (TAK1 inhibitor, 1 μM), SB203580 (p38MAPK inhibitor, 5 μM), PD98059 (MEK1 inhibitor, 50 μM), U0126-EtOH (MEK1/2 inhibitor, 1 μM) FR180204 (ERK1/2 inhibitor, 1 μM), SCH772984 (ERK1/2 inhibitor, 250 nM), SP600125 (JNK inhibitor, 10 μM), T5224 (AP-1 inhibitor, 20 μM), MeOSuc-AAPV-CMK (serine protease inhibitor, 1 μM), JNJ-10311795 (cathepsin G inhibitor, 10 μM), BAY 85-8501 (a specific elastase inhibitor, 30 μM), GSK-872 (RIPK3 inhibitor, 5 μM), DHMEQ (NF-kB inhibitor, 20 μM) (all purchased from MedChemExpress), and STAT3 Inhibitor VI (STAT3 inhibitor; Calbiochem, 50 μM).

### Cytokine determinations

Samples were assayed for cytokine/chemokine concentrations using the following assays (all from R&D Systems): Proteome Profiler Mouse Cytokine Array Kit; CXCL1/KC DuoSet; CXCL2/MIP-2 DuoSet; TNF-α DuoSet; IL-1βDuoSet; IL-6 DuoSet; IL-18 DuoSet; CCL2/JE/MCP-1 DuoSet; M-CSFDuoSet; GM-CSF DuoSet. The lower detection limits of these assays were 3.9 (CCL2), 7.8 (GM-CSF), 15.6 (IL-1β, IL-6, M-CSF, CXCL1 and 2), 31.3 (TNF-α), and 46.9 pg/ml (IL-18). Neutrophil elastase was measured using the Mouse Neutrophil Elastase/ELA2 DuoSet ELISA (R&D Systems). Proteome Profiler Mouse Cytokine Array images were captured with Bio-Rad’s ChemiDoc XRS system, and the optical integrated density was calculated using ImageJ software, each spot being normalized to the average intensity of reference spots. Neutrophil depletion was achieved as previously described (Lentini et al, 2023). Briefly, 100 µg of rat monoclonal anti-mouse Ly-6G Ab (clone 1A8) or IgG2_A_ isotype control was injected i.p. at 24 h before z-IETD-fmk or vehicle treatment.

### Gene expression qPCR measurements

Total RNA was extracted from 3 × 10^6^ bone marrow neutrophils after 3 h of treatment with z-IETD-fmk (50 μM) or vehicle and retrotranscribed. Expression of the genes encoding *IL-1β*, *kcnj4* (potassium inwardly-rectifying channel, subfamily J, member 4), *zbp1* (Z-DNA binding protein 1), *folr1* (folate receptor 1), *ifnβ*, and *cxcl1*was determined by qPCR using a CFX96 Touch Real-Time PCR Detection System (Bio-Rad), exactly as described previously (Biondo et al, 2005).

### RNA-seq library preparation and sequencing

Bone marrow-derived macrophages were cultured for one week in medium containing macrophage colony-stimulating factor, as previously described (Lentini et al, 2022). Bone marrow-derived neutrophils were obtained as described above. Total RNA was extracted from TRIzol lysates of 3 × 10^6^ bone marrow neutrophils or macrophages treated for 3 h with z-IETD-fmk (50 μM) or vehicle in the course of four separate experiments. RNA integrity was validated by RNA integrity number algorithm analysis, as previously described (Lentini et al, 2021; Lentini et al, 2018). Sequencing libraries were prepared using the Illumina Stranded Total RNA Prep Ribo-Zero Plus for stranded total RNA sequencing according to the manufacturer’s instructions. Total RNA sequencing was performed on an Illumina NextSeq2000 instrument using a NextSeq 2000 P3 flow cell and reagents. Raw sequencing data were uploaded to the Galaxy web platform (https://usegalaxy.org/) for analysis. Reads were cleaned with Trimmomatic (Galaxy Version 0.39+galaxy2), mapped on the *Mus musculus* genome with HISAT2 (Galaxy Version 2.2.1+galaxy1), and annotated with annotate MyIDs (Galaxy Version 3.18.0+galaxy0). Gene counts (featureCounts, Galaxy Version 2.0.8+galaxy0) were analyzed for differential expression with limma-voom (Galaxy Version 3.58.1+galaxy0)(Phipson et al, 2016). Comparisons were normalized using a trimmed mean of *M* values (TMM), and raw *P* values were adjusted using Benjamini and Hochberg multiple tests. Adjusted *P* values lower than 0.05 were considered significant. Volcano plots were generated using the GraphPad Software; pathway enrichment analysis was performed by the ShinyGO 0.81 software (https://bioinformatics.sdstate.edu/go/); heatmaps were generated using Heatmapper (http://www.heatmapper.ca/). Raw sequencing reads are publicly available at GEO (GSE 288233).

### Western blot analysis

Bone marrow-derived neutrophils were analyzed as described (Lentini et al, 2022; Lentini et al, 2021), with some modifications. Briefly, cells were seeded on 6-well plates (5 × 10^6^/well in 2 ml of RPMI 1640 supplemented with 10% FCS) and collected at 3 h after the addition of z-IETD-fmk or vehicle as a control. When indicated, peritoneal cell lysates were obtained from mice at 4 h after i.p. injection with z-IETD-fmk or vehicle. Both neutrophils and peritoneal cells were washed three times with ice-cold PBS and lysed in RIPA lysis buffer [20 mM Tris-HCl (pH 7.5), 150 mM NaCl, 1 mM Na_2_EDTA, 1 mM EGTA, 1% NP-40, 1% sodium deoxycholate, 2.5 mM sodium pyrophosphate, 1 mM beta-glycerophosphate, 1 mM Na_3_VO_4_, 1 µg/ml leupeptin, 1 mM PMSF, 3 mM DFP, 10 mM Na_4_P_2_O_7_, and 0.5 mM AEBSF]. Lysates were then centrifuged at 13,000×*g* for 15 min at 4 °C to eliminate cellular debris. Protein concentration in each sample was determined using the Micro BCA Protein Assay Kit. Protein samples (30 μg of protein per lane) were run on precast Bolt Bis-Tris 4–12% gels with 1× MOPS buffer and transferred to PVDF membranes. Membranes were washed in TBS-T (Tris Buffered Saline with 0.1% Tween-20) and blocked with TBS-T containing 5% BSA for 2 h. Membranes were subsequently incubated with primary antibodies in TBS-T containing 1% BSA at 4 °C overnight. The following primary antibodies were used: phospho-RIP (Ser166) (E7G6O) rabbit mAb, RIP (D94C12) XP rabbit mAb, phospho-RIP3 (Thr231/Ser232) (E7S1R) rabbit mAb, RIP3 (D8J3L) rabbit mAb, anti-mouse IL-1 beta/IL-1F2 antibody, anti-Caspase-1 (p10) mouse mAb (Casper-2), anti-Caspase-1(p20) mouse mAb (Casper-1), anti-GSDMD antibody [EPR20859] and anti-beta-actin. After incubation, membranes were washed with TBS-T and incubated with secondary antibody (anti-rabbit, anti-mouse, or anti-goat IgG HRP-linked antibodies) for 2 h at room temperature in TBS-T containing 1% BSA. Protein bands were visualized by Immobilon Forte Western HRP substrate and detected using Bio-Rad’s ChemiDoc XRS system. Beta-actin was used as a loading control.

### Immuno-staining and flow cytometric analysis

Single-cell suspensions were obtained from spleen, bone marrow, peritoneal lavage, and blood of mice treated with z-IETD-fmk or vehicle. Flow cytometry procedures and instrument setup were carried out as previously described, with some modifications (Brummelman et al, 2019). Briefly, cells were collected from mice, washed three times with PBS, and stained in the dark for 20 min with LIVE/DEAD Fixable Aqua Dead Cell Stain Kit, according to the manufacturer’s instructions. Negative cells were considered viable. Cells were then blocked with Fc blocking reagent (Clone 24G2) for 20 min and stained for surface markers for 20 min in the dark with different antibody mixes. All used antibodies are listed in the Reagents and Tools Table. Cells were analyzed on FACSymphony or FACS Canto II flow cytometers (both from BD Bioscience). Data analysis was performed using FlowJo software.

### Statistical analysis

Survival data were analyzed by Kaplan–Meier survival plots. All other data were analyzed by the Mann–Whitney test. Differences were considered significant when *P* values were less than 0.05.

## Supplementary information

Peer Review File

Source data Fig. 1

Source data Fig. 2

Source data Fig. 3

Source data Fig. 4

Source data Fig. 5

Source data Fig. 6

Source data Fig. 7

Figure EV1 Source Data

Figure EV2 Source Data

Figure EV3 Source Data

Figure EV4 Source Data

Figure EV5 Source Data

Expanded View Figures

## Data Availability

Tables summarizing global genome gene expression analysis can be found at NCBI Gene Expression Omnibus, GEO accession GSE288233. Raw RNA sequence data (Sequence read archive or SRA) have been deposited at the NHI Submission Portal BioProject PRJNA1214786 (Transcriptome modulation by Caspase-8 inhibition): https://www.ncbi.nlm.nih.gov/bioproject/PRJNA1214786/. The source data of this paper are collected in the following database record: biostudies:S-SCDT-10_1038-S44319-026-00813-5.
