# Supplementary figures and images for: Caspase-8 silences cell death-independent constitutive immune activation driven by tonic TNF-α
Source: EMBO Rep. 2026 Jun 8;27(14):4054–78. doi: 10.1038/s44319-026-00813-5 (PMC13400634; doi:10.1038/s44319-026-00813-5)

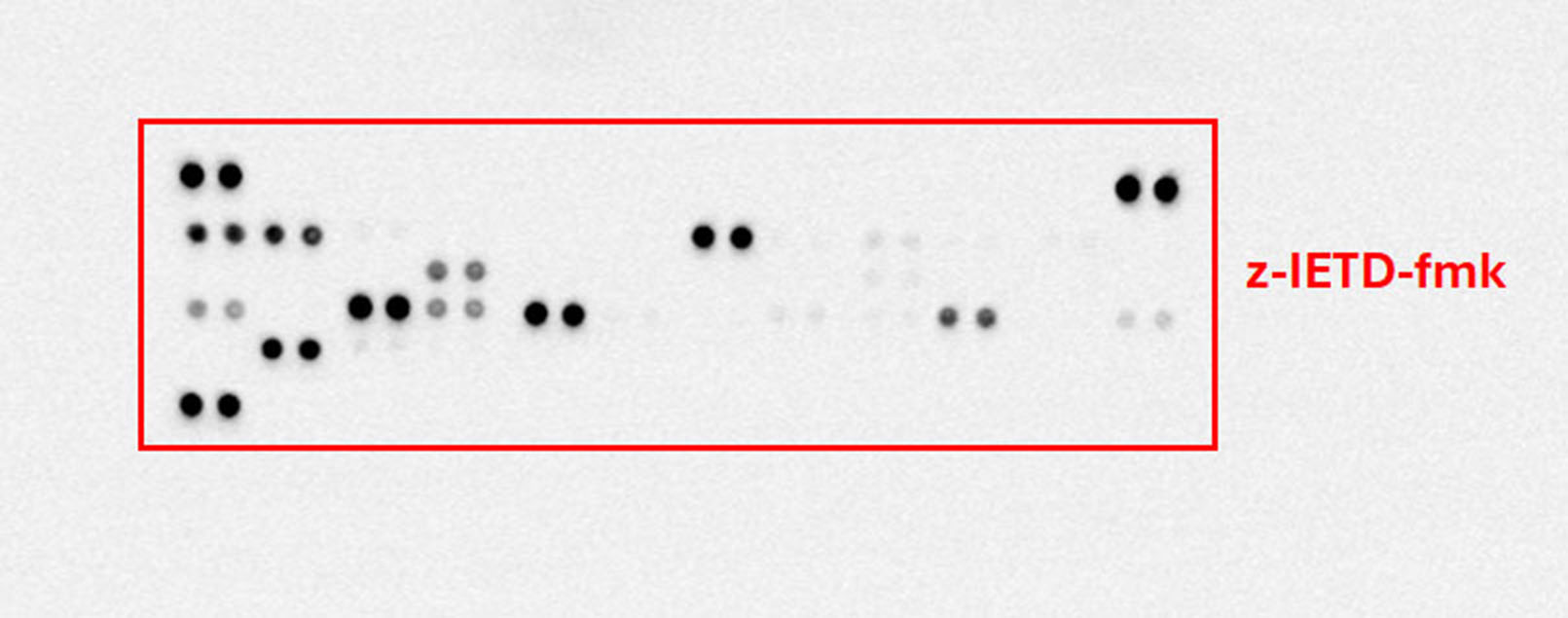

Supplement: Supplementary file 2 — Source data Fig. 1 [file 44319_2026_813_MOESM2_ESM.zip › FIG 1/FIG 1D/UNCROPPED PROFILER IETD.tif]

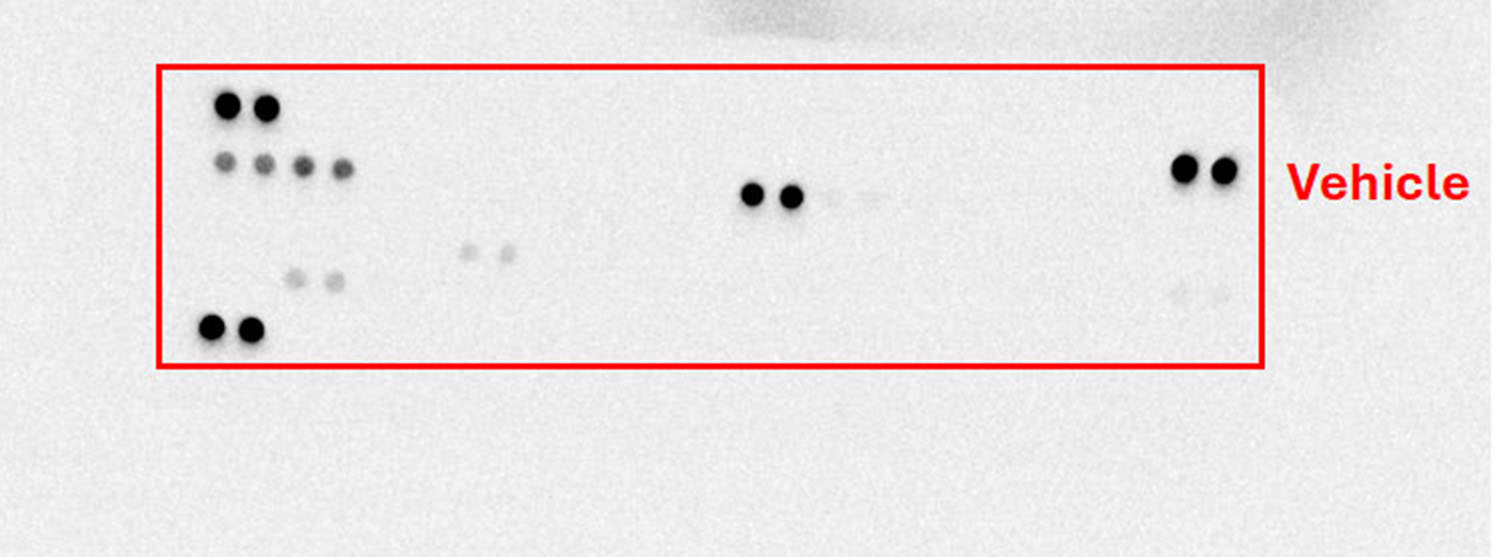

Supplement: Supplementary file 2 — Source data Fig. 1 [file 44319_2026_813_MOESM2_ESM.zip › FIG 1/FIG 1D/UNCROPPED PROFILER VEHICLE.tif]

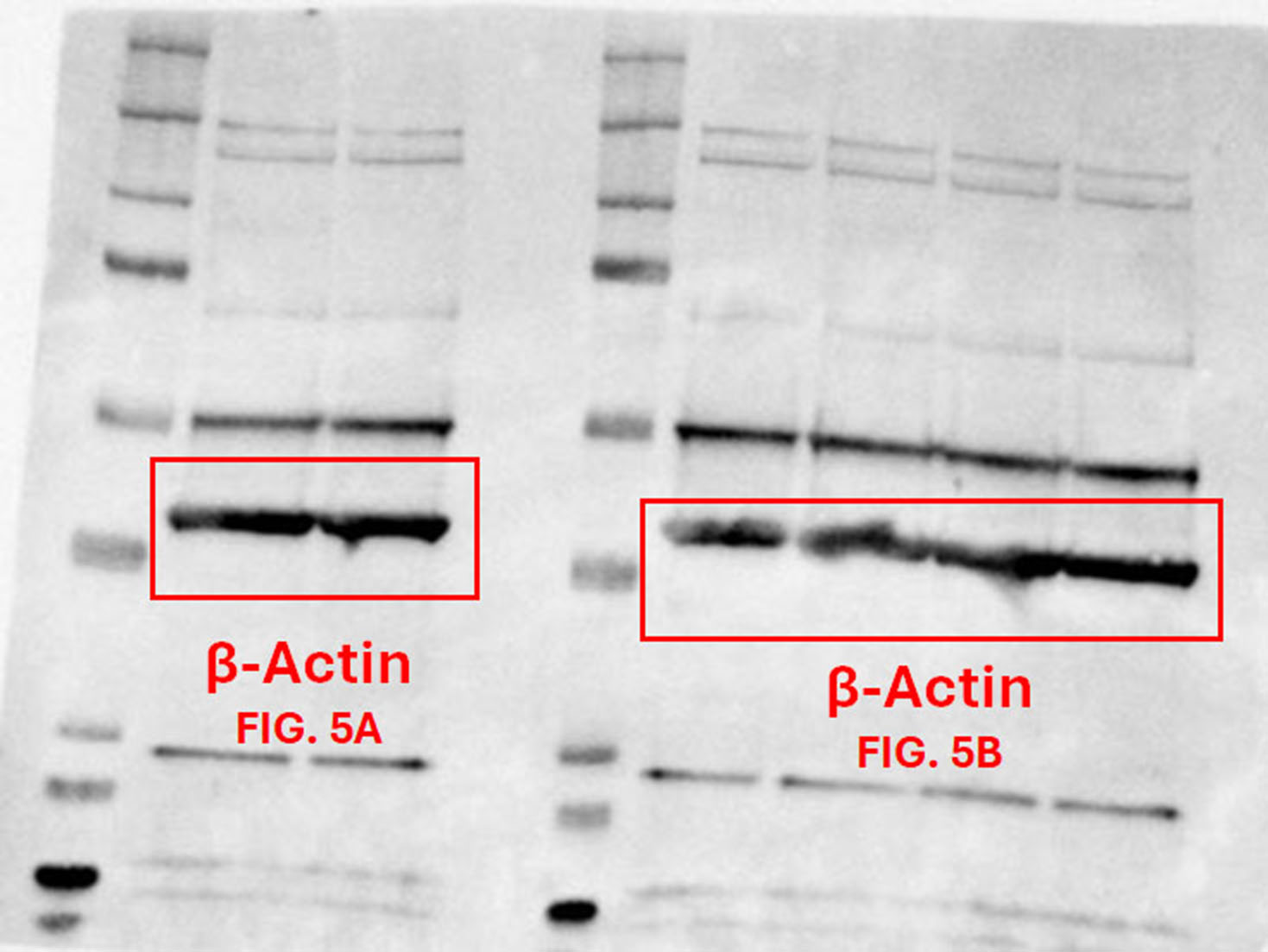

Supplement: Supplementary file 6 — Source data Fig. 5 [file 44319_2026_813_MOESM6_ESM.zip › FIG 5/FIG 5A/UNCROPPED beta ACTIN.tif]

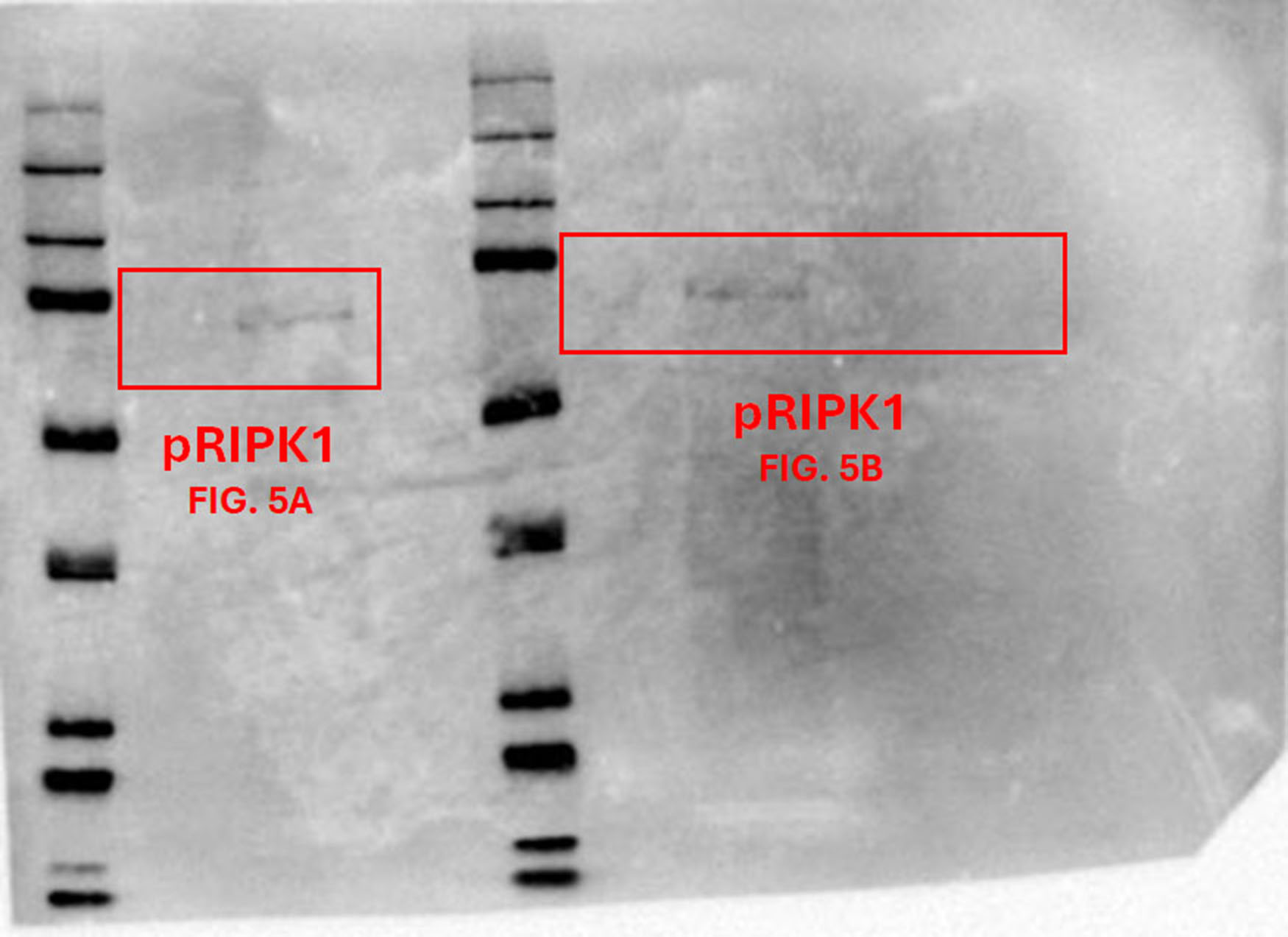

Supplement: Supplementary file 6 — Source data Fig. 5 [file 44319_2026_813_MOESM6_ESM.zip › FIG 5/FIG 5A/UNCROPPED pRIPK1.tif]

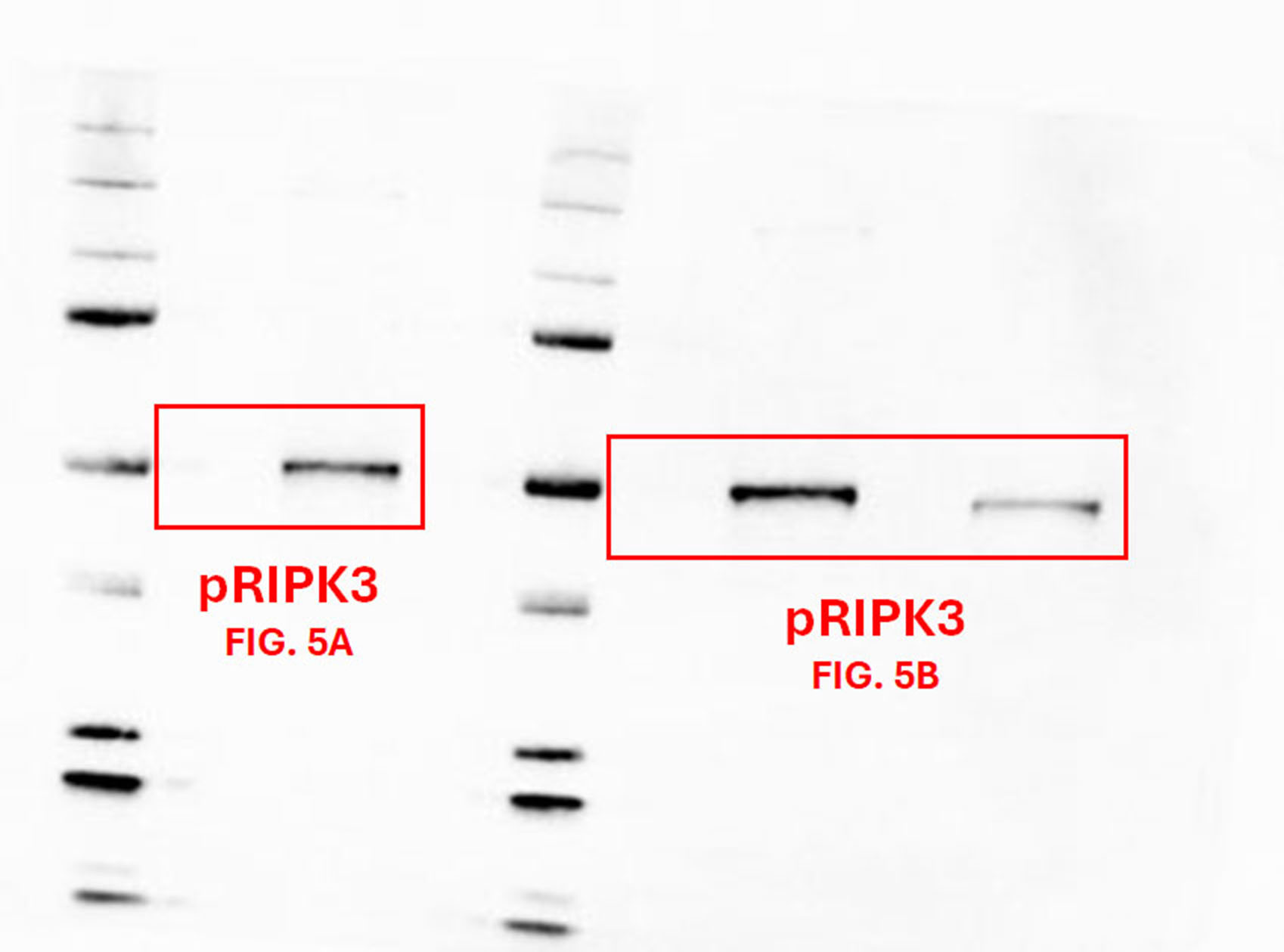

Supplement: Supplementary file 6 — Source data Fig. 5 [file 44319_2026_813_MOESM6_ESM.zip › FIG 5/FIG 5A/UNCROPPED pRIPK3.tif]

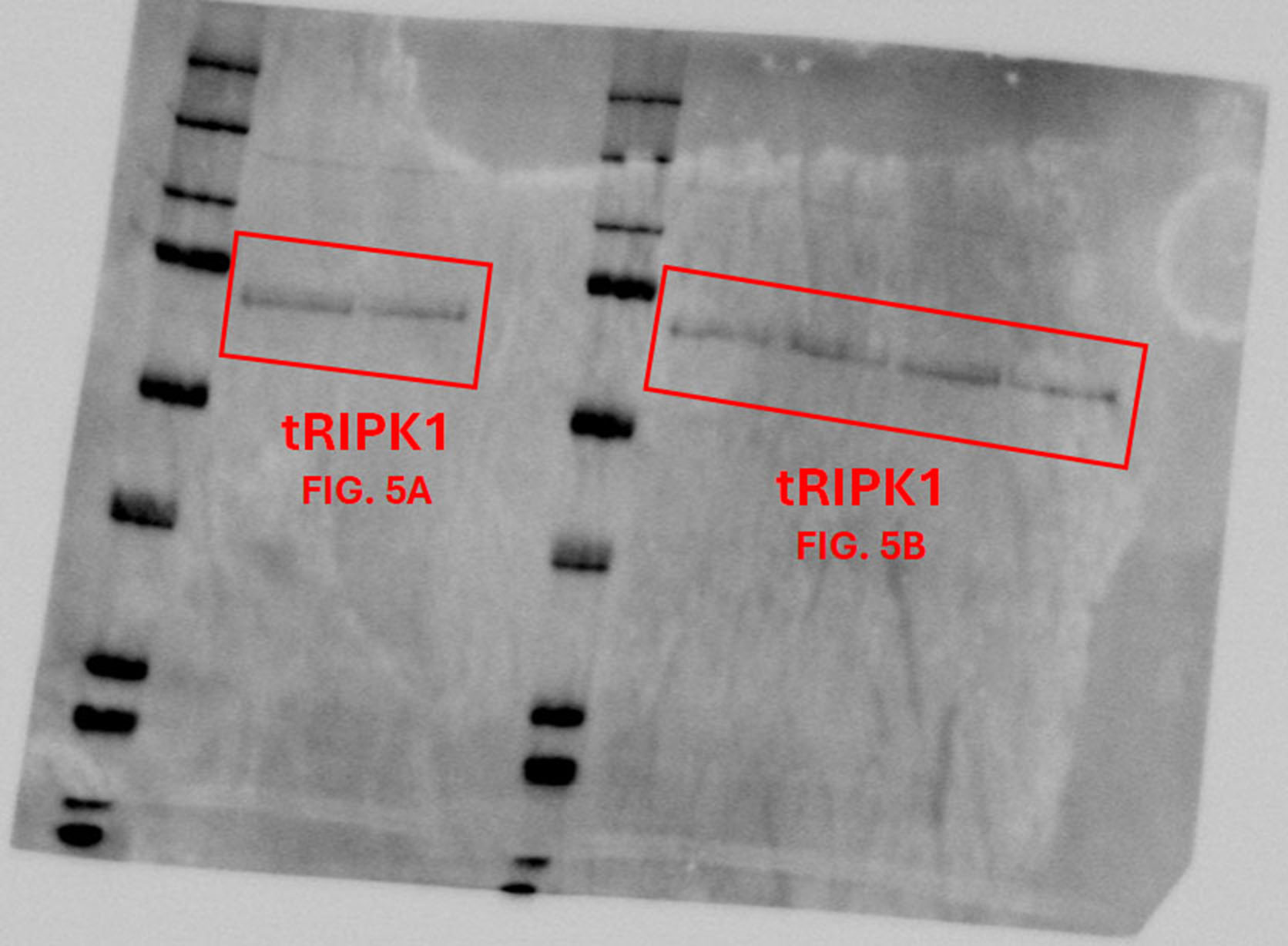

Supplement: Supplementary file 6 — Source data Fig. 5 [file 44319_2026_813_MOESM6_ESM.zip › FIG 5/FIG 5A/UNCROPPED tRIPK1.tif]

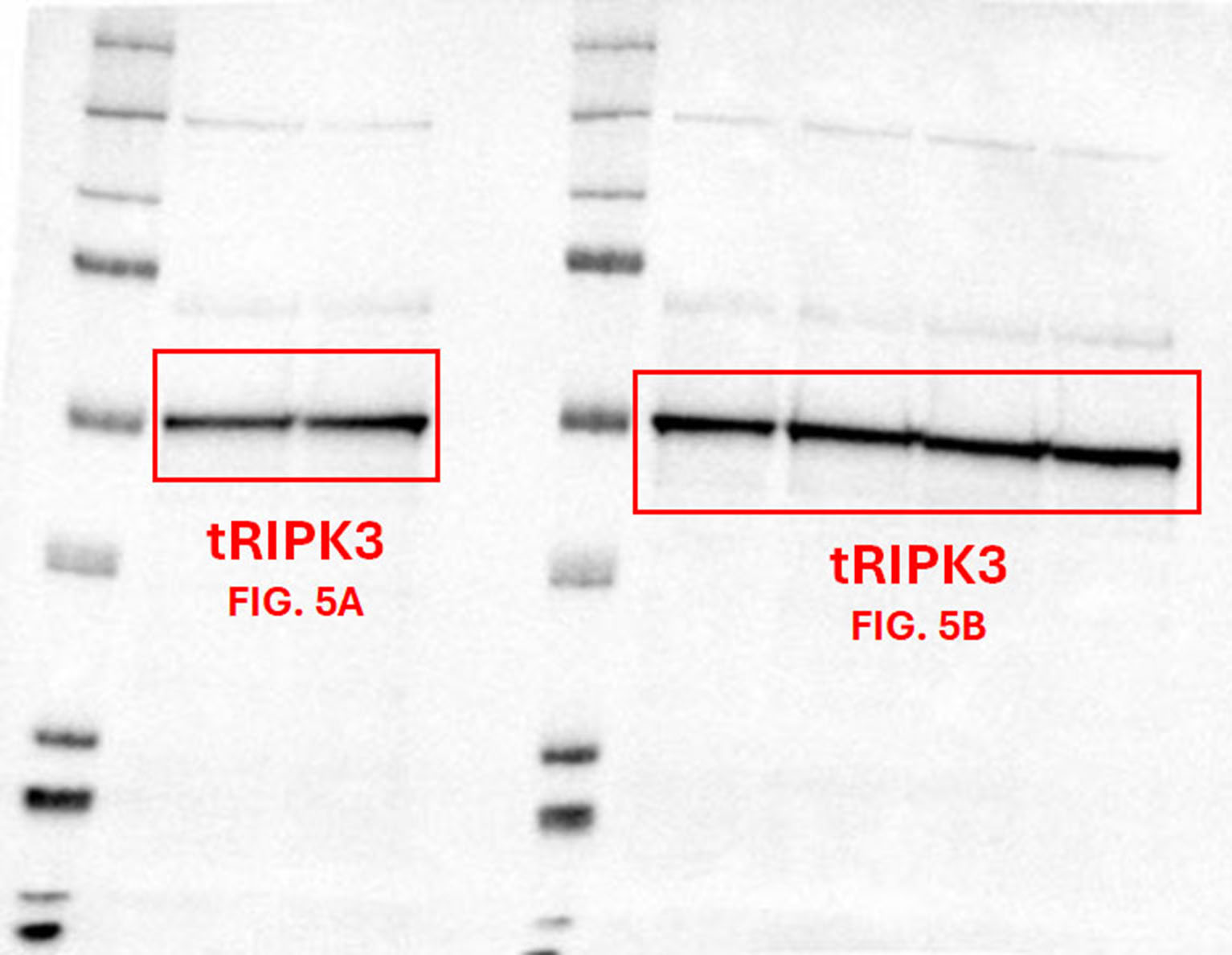

Supplement: Supplementary file 6 — Source data Fig. 5 [file 44319_2026_813_MOESM6_ESM.zip › FIG 5/FIG 5A/UNCROPPED tRIPK3.tif]

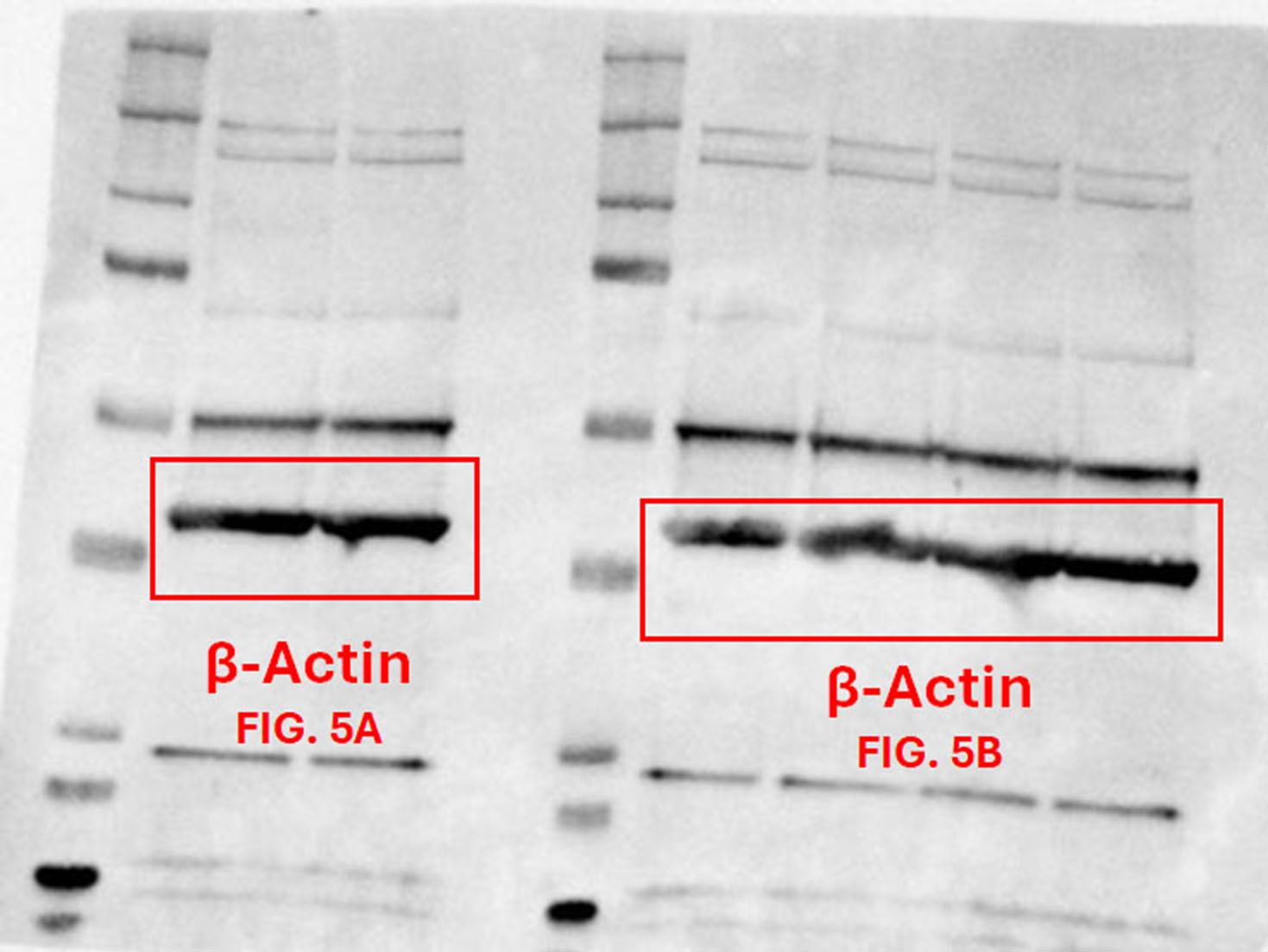

Supplement: Supplementary file 6 — Source data Fig. 5 [file 44319_2026_813_MOESM6_ESM.zip › FIG 5/FIG 5B/UNCROPPED beta ACTIN.tif]

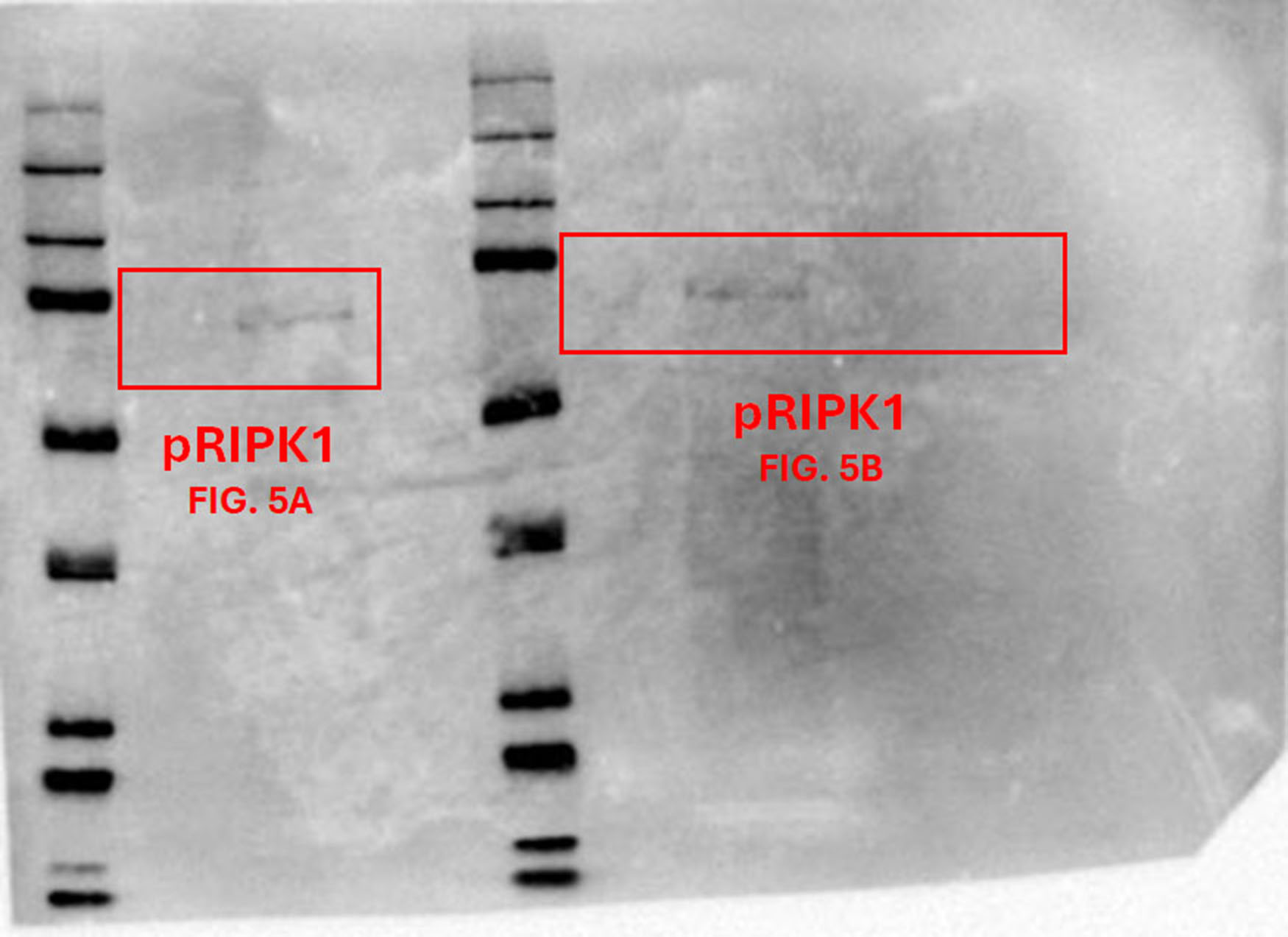

Supplement: Supplementary file 6 — Source data Fig. 5 [file 44319_2026_813_MOESM6_ESM.zip › FIG 5/FIG 5B/UNCROPPED pRIPK1.tif]

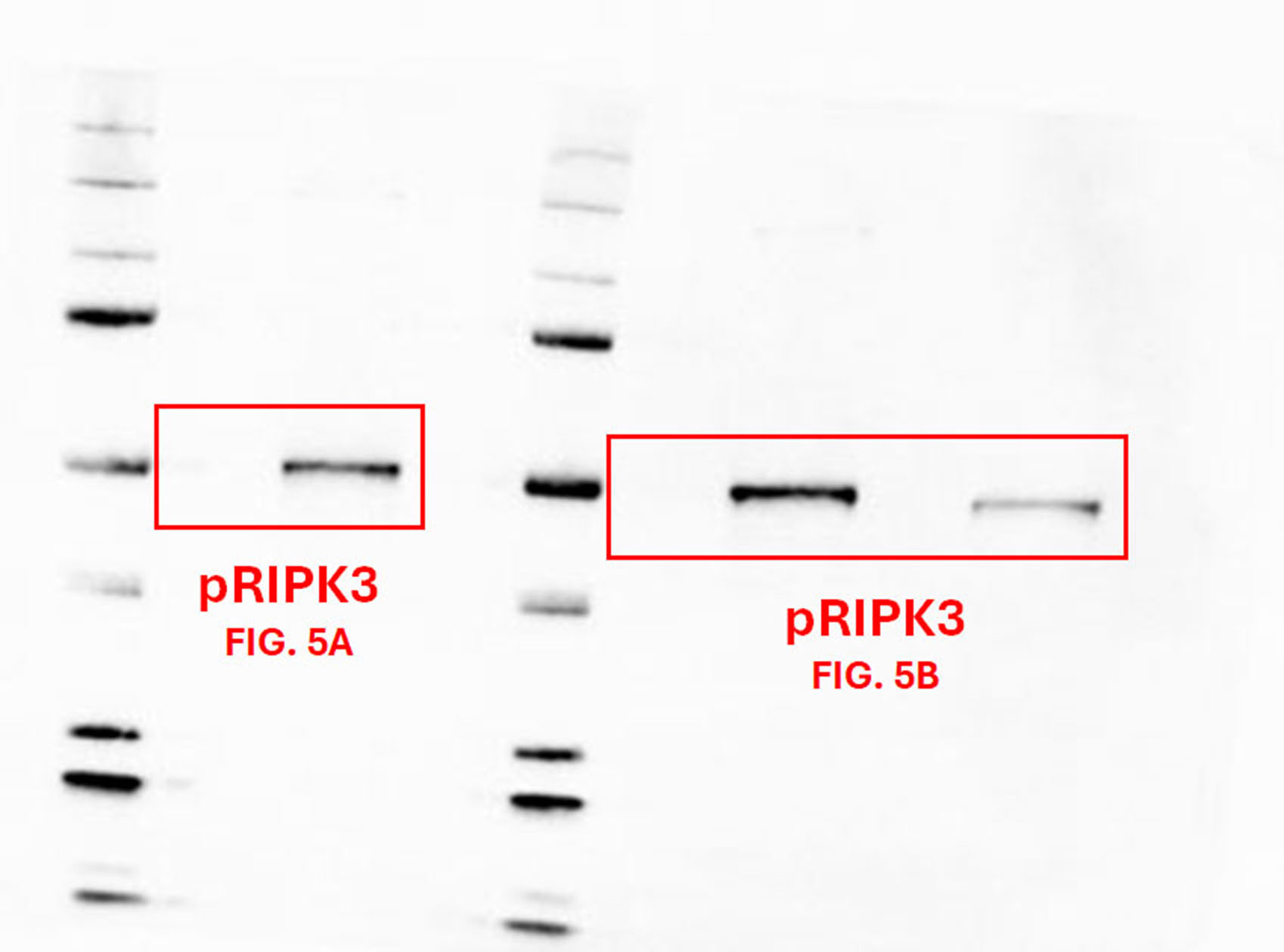

Supplement: Supplementary file 6 — Source data Fig. 5 [file 44319_2026_813_MOESM6_ESM.zip › FIG 5/FIG 5B/UNCROPPED pRIPK3.tif]

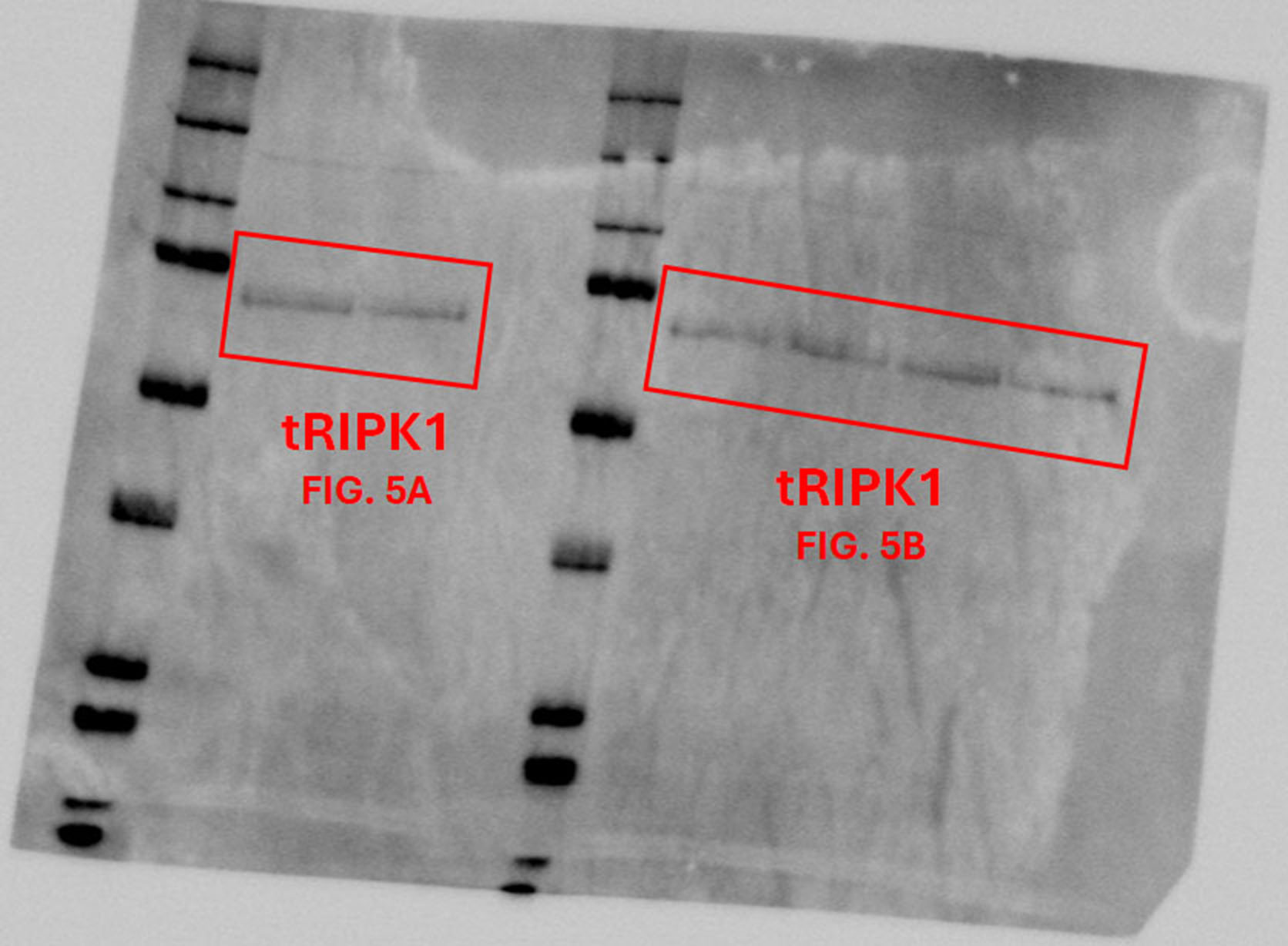

Supplement: Supplementary file 6 — Source data Fig. 5 [file 44319_2026_813_MOESM6_ESM.zip › FIG 5/FIG 5B/UNCROPPED tRIPK1.tif]

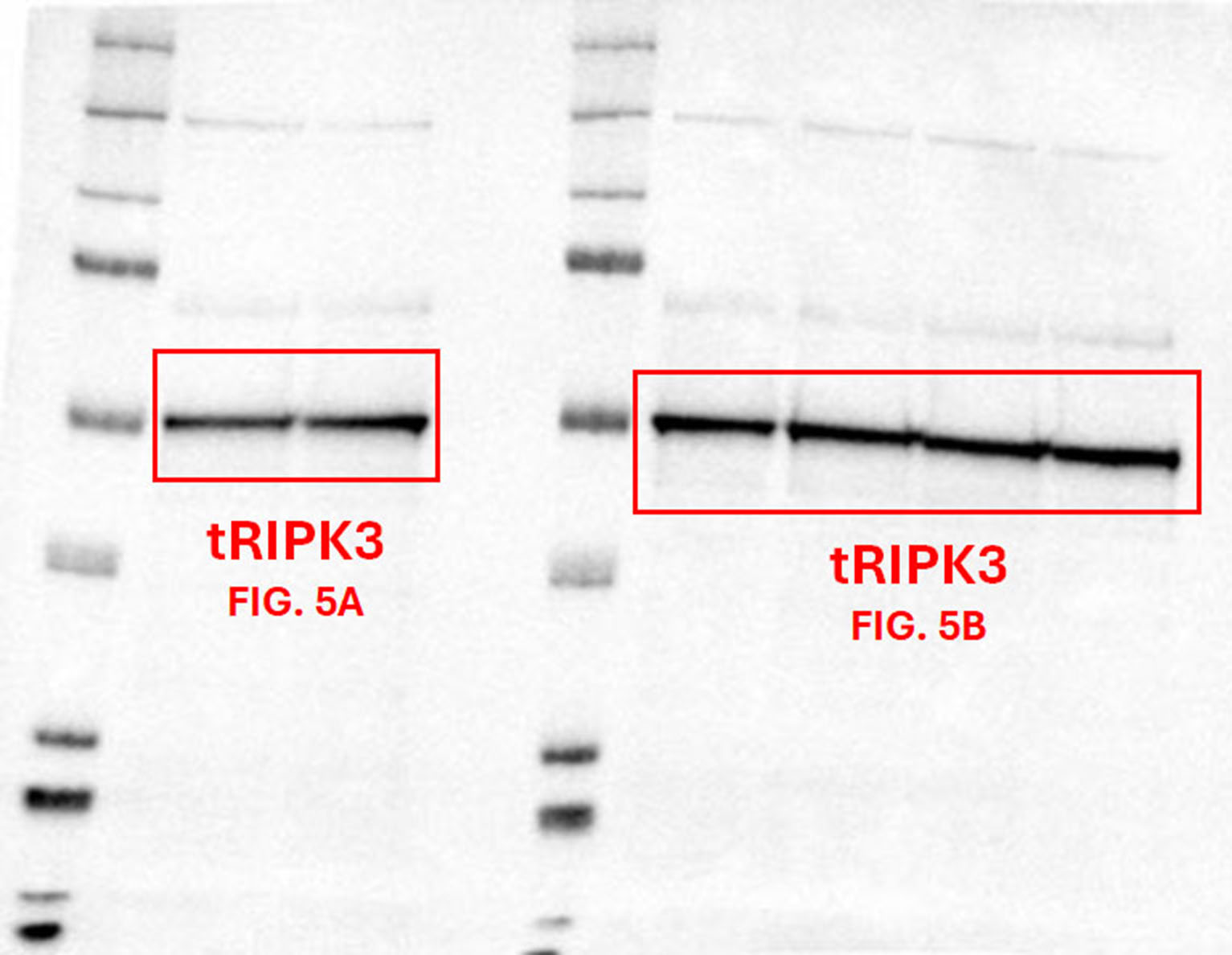

Supplement: Supplementary file 6 — Source data Fig. 5 [file 44319_2026_813_MOESM6_ESM.zip › FIG 5/FIG 5B/UNCROPPED tRIPK3.tif]

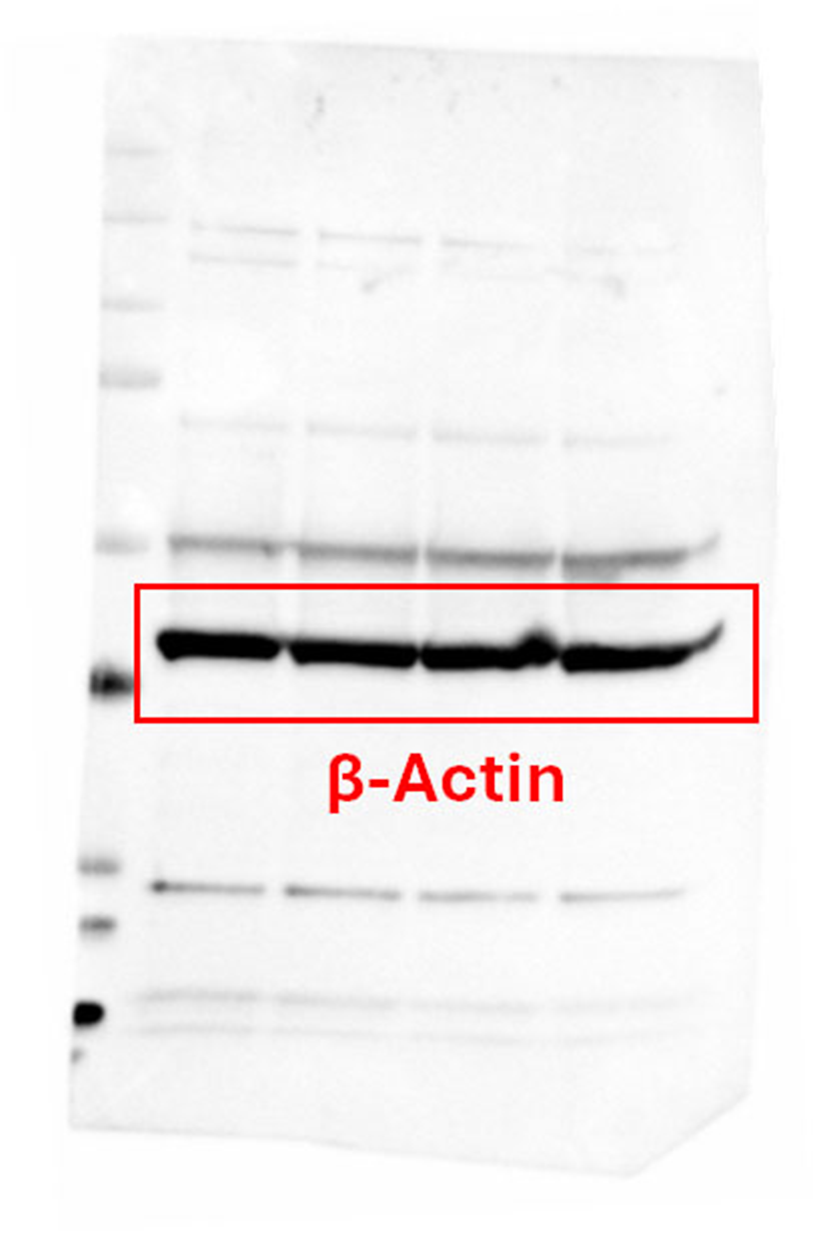

Supplement: Supplementary file 6 — Source data Fig. 5 [file 44319_2026_813_MOESM6_ESM.zip › FIG 5/FIG 5C/UNCROPPED beta ACTIN.tif]

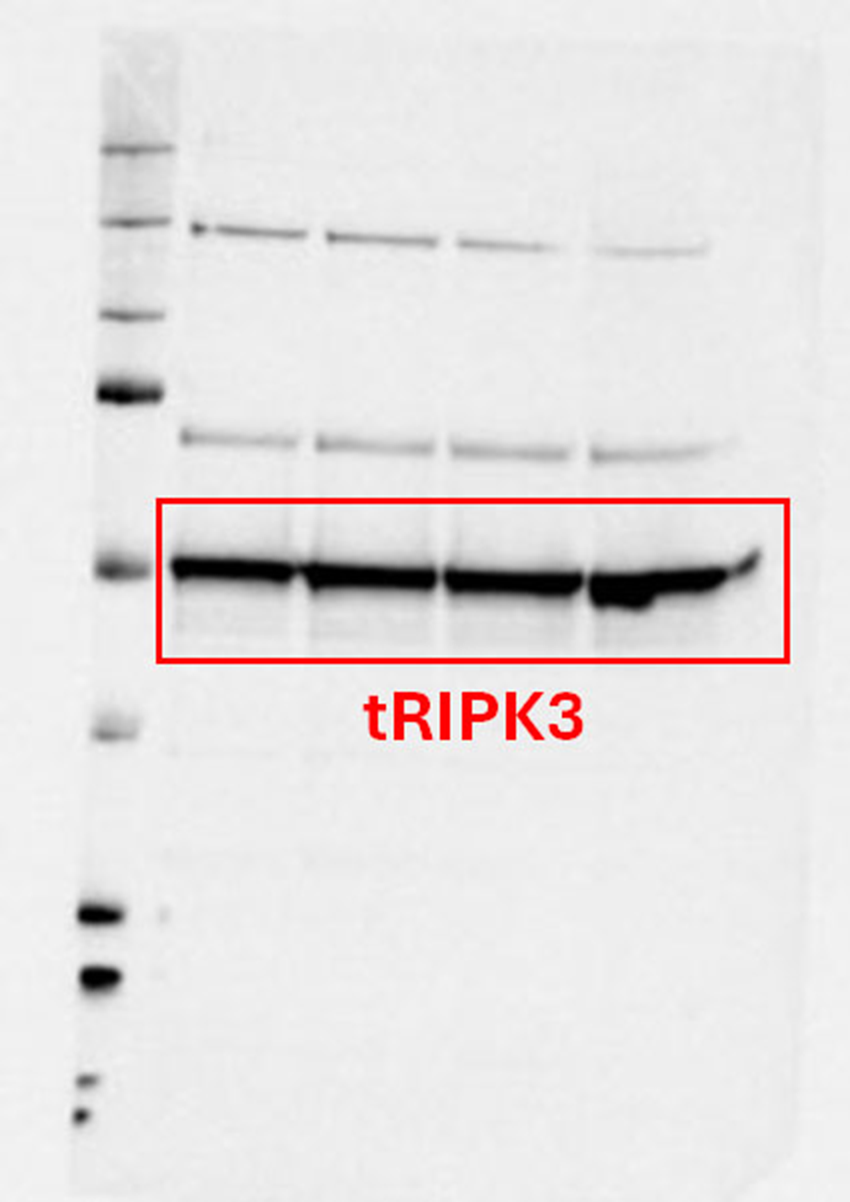

Supplement: Supplementary file 6 — Source data Fig. 5 [file 44319_2026_813_MOESM6_ESM.zip › FIG 5/FIG 5C/UNCROPPED tRIPK3.tif]

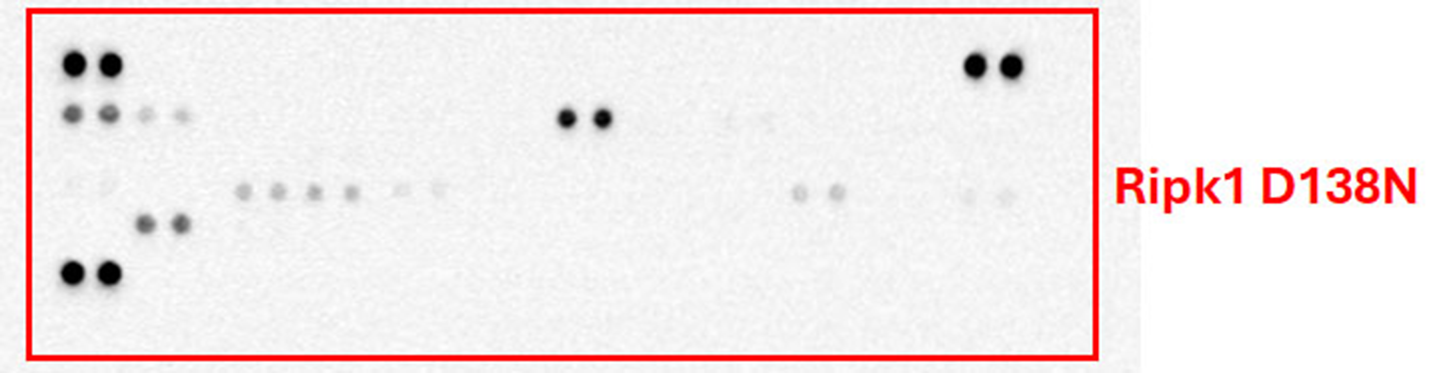

Supplement: Supplementary file 6 — Source data Fig. 5 [file 44319_2026_813_MOESM6_ESM.zip › FIG 5/FIG 5E/5E Ripk1 D138N.tif]

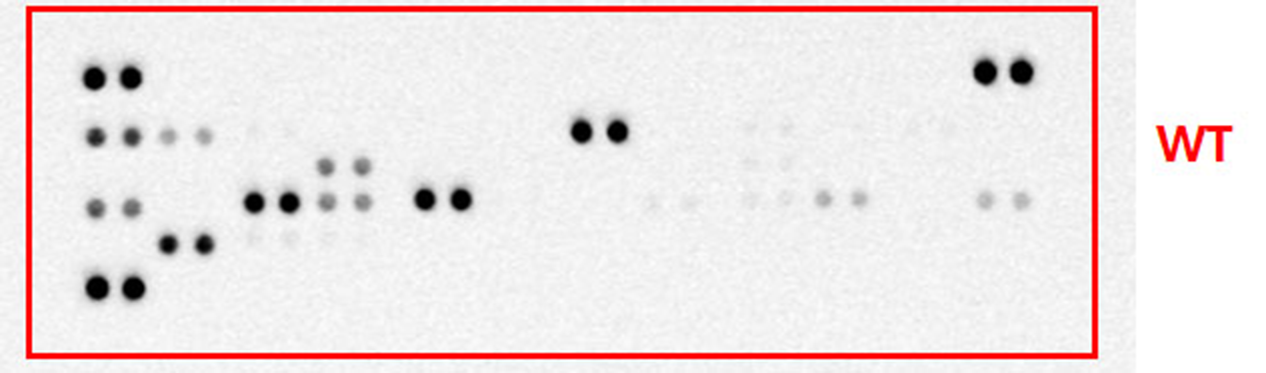

Supplement: Supplementary file 6 — Source data Fig. 5 [file 44319_2026_813_MOESM6_ESM.zip › FIG 5/FIG 5E/5E WT.tif]

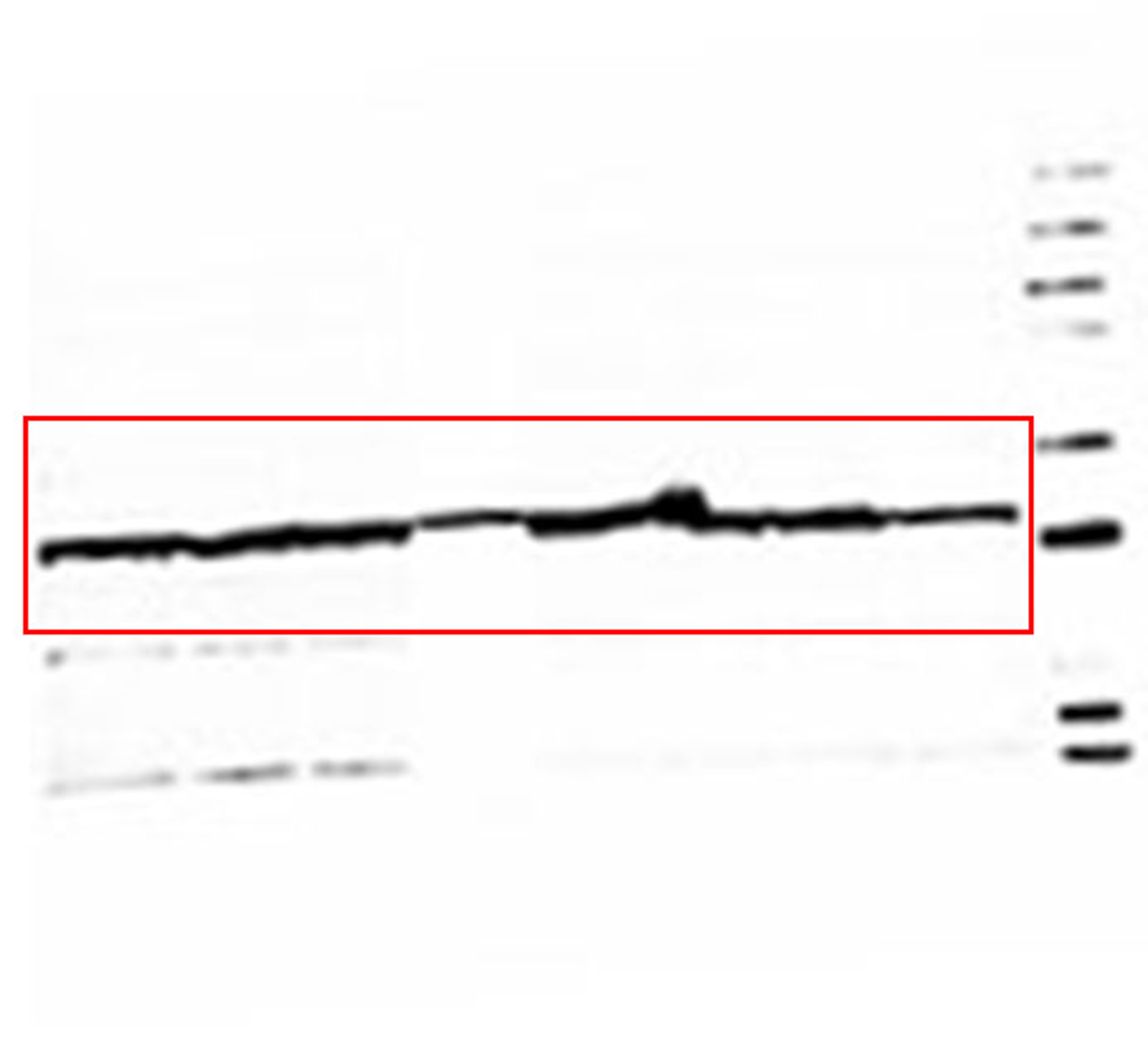

Supplement: Supplementary file 8 — Source data Fig. 7 [file 44319_2026_813_MOESM8_ESM.zip › FIG 7/FIG 7C/UNCROPPED B-ACTIN.tif]

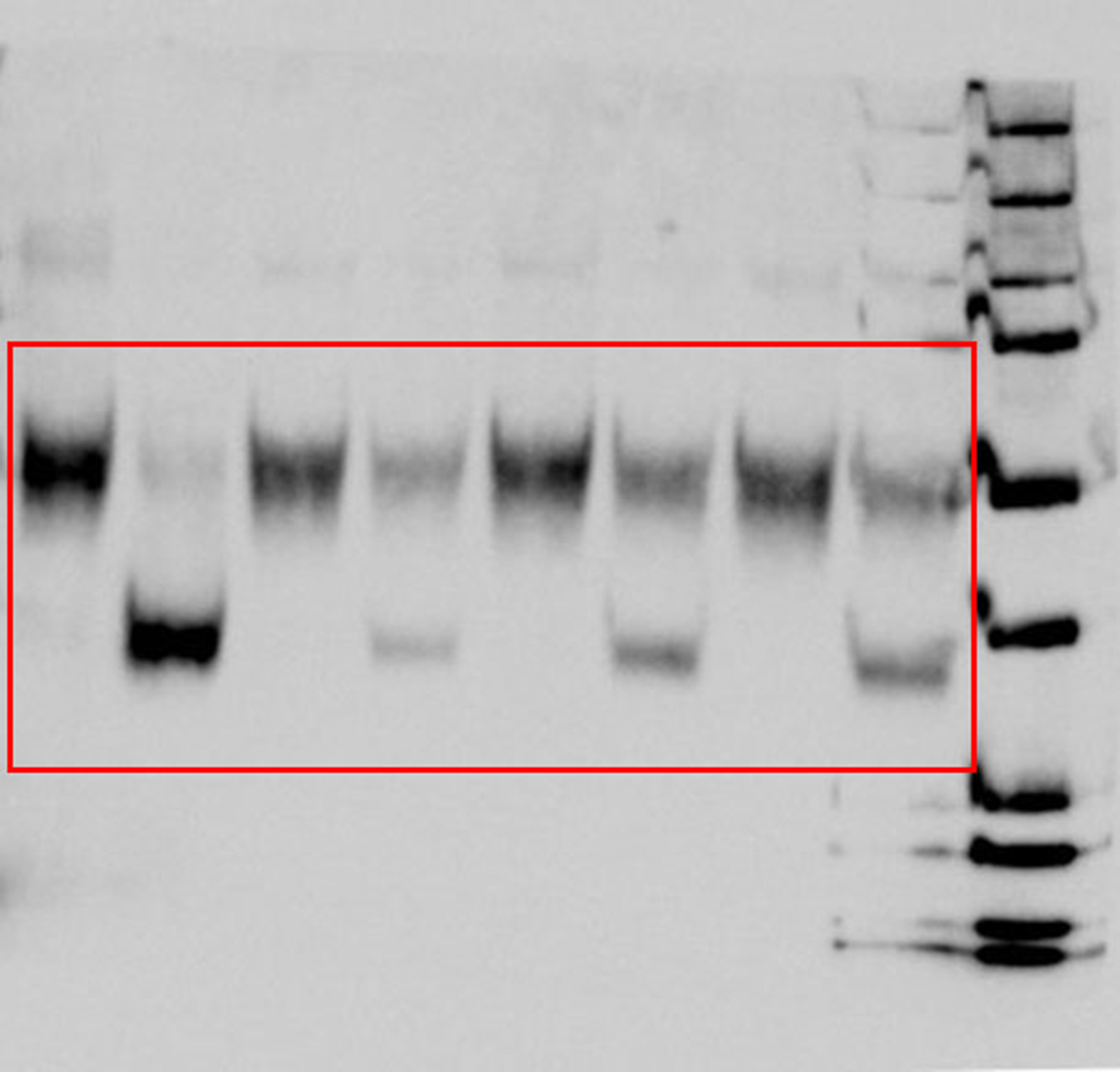

Supplement: Supplementary file 8 — Source data Fig. 7 [file 44319_2026_813_MOESM8_ESM.zip › FIG 7/FIG 7C/UNCROPPED GSDMD.tif]

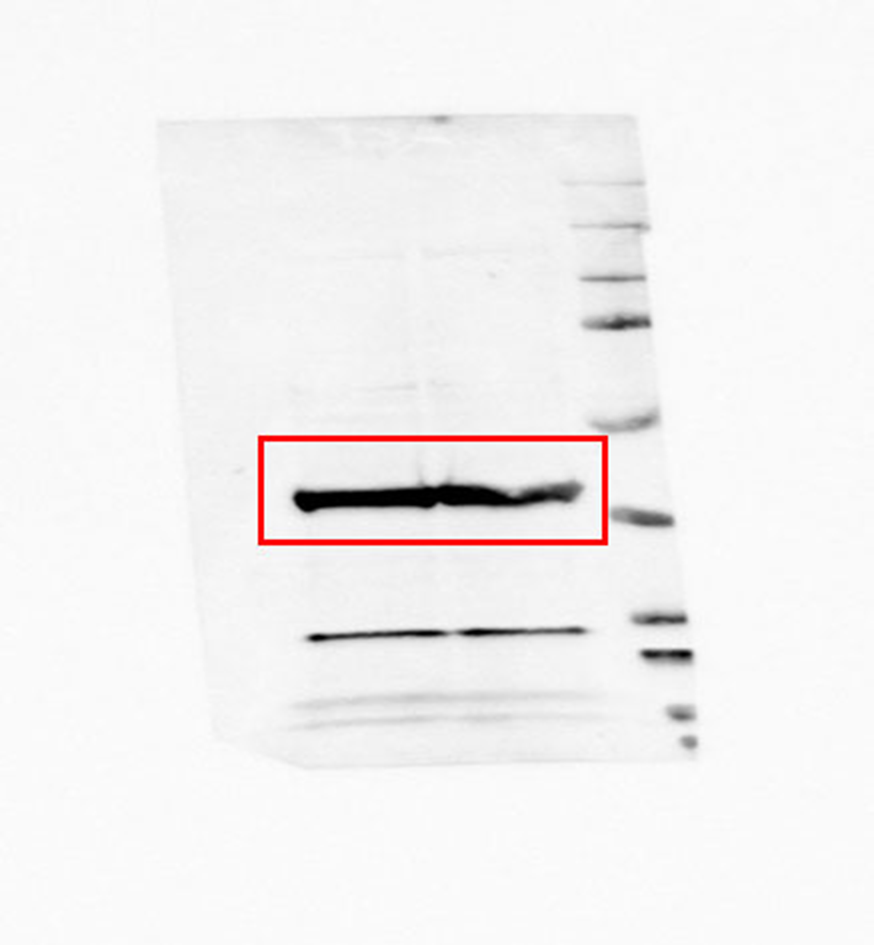

Supplement: Supplementary file 8 — Source data Fig. 7 [file 44319_2026_813_MOESM8_ESM.zip › FIG 7/FIG 7E/UNCROPPED Beta actin.tif]

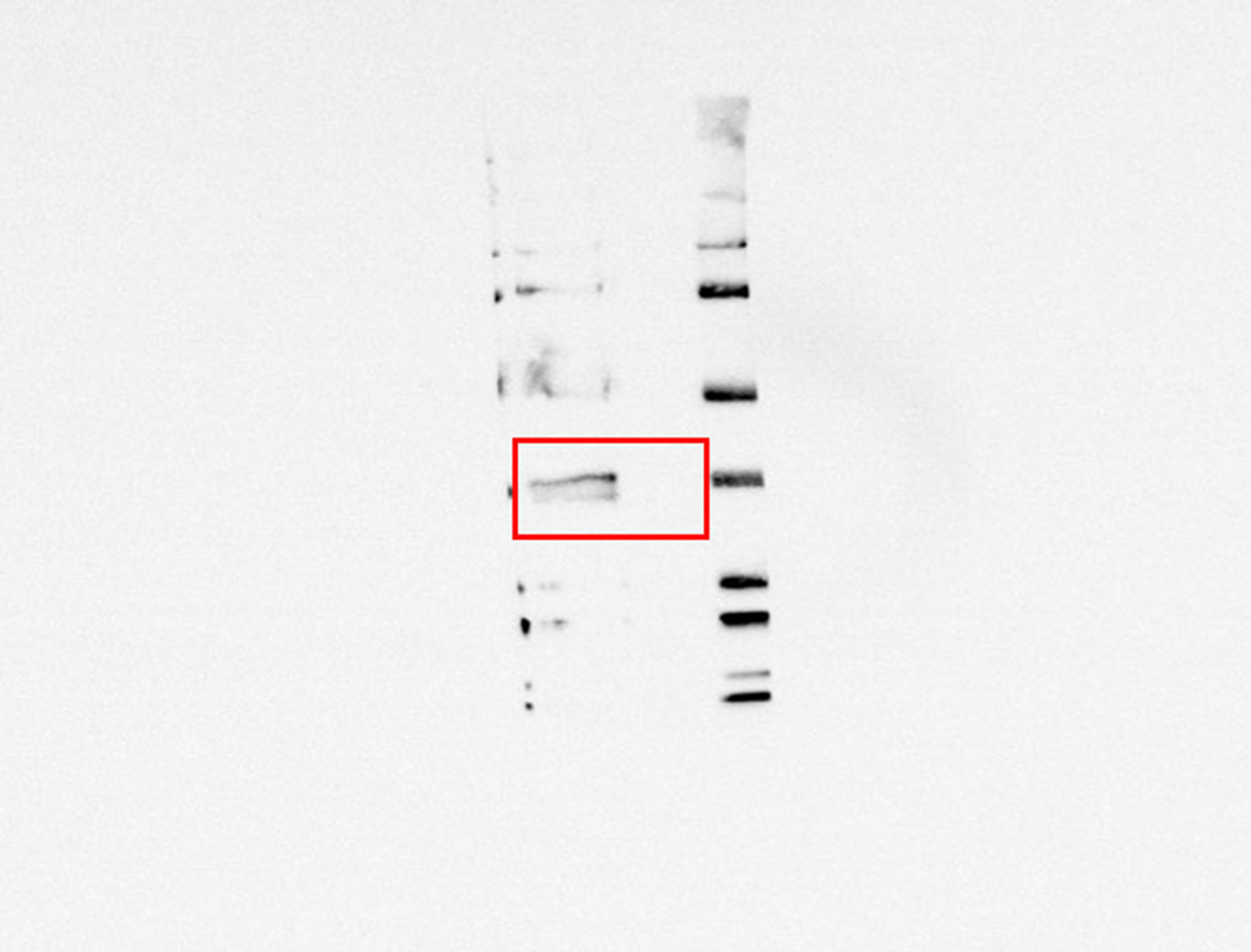

Supplement: Supplementary file 8 — Source data Fig. 7 [file 44319_2026_813_MOESM8_ESM.zip › FIG 7/FIG 7E/UNCROPPED Phospho-ERK1_2.tif]

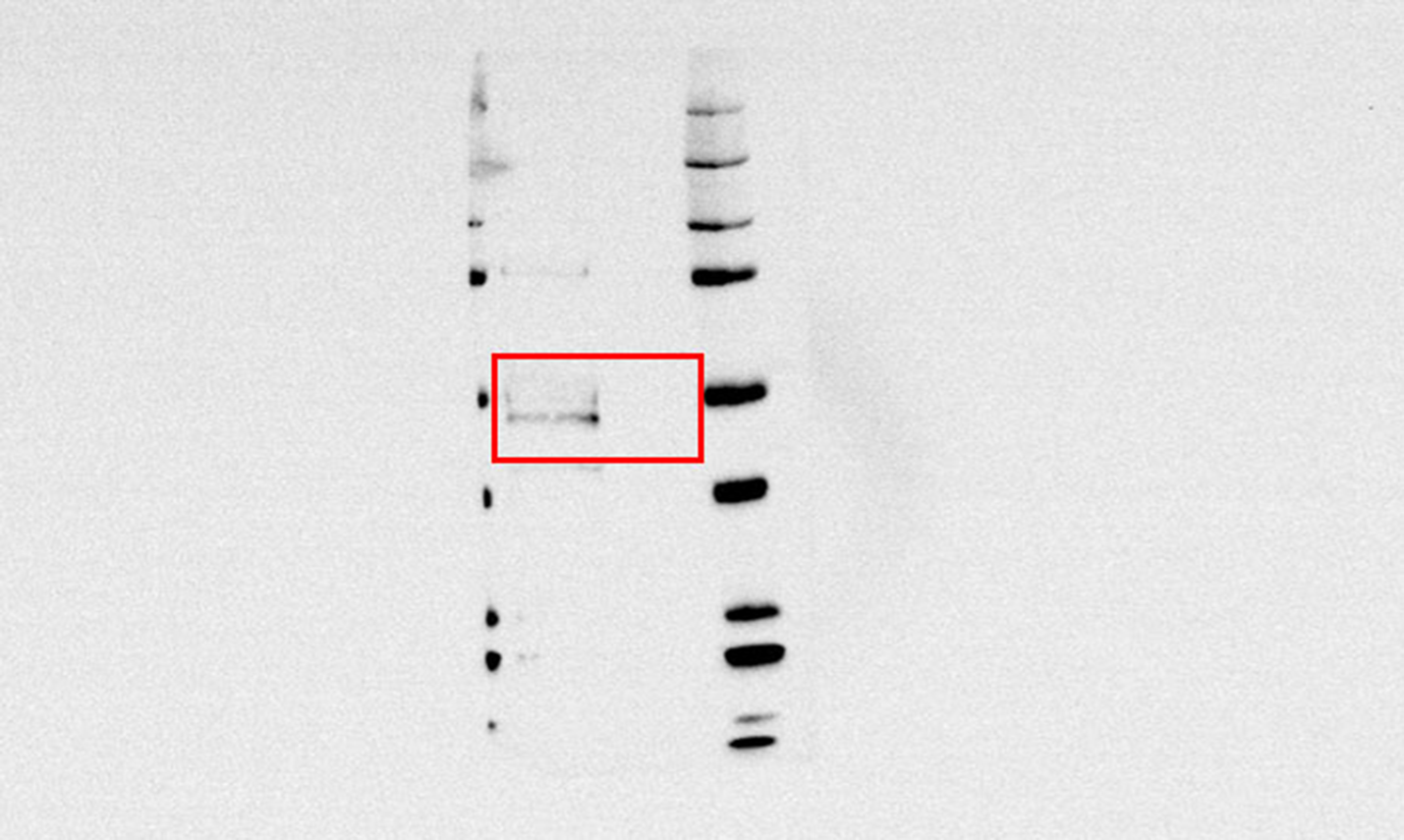

Supplement: Supplementary file 8 — Source data Fig. 7 [file 44319_2026_813_MOESM8_ESM.zip › FIG 7/FIG 7E/UNCROPPED Phospho-SAPKJNK.tif]
