# Supplementary material for: Caspase-8 silences cell death-independent constitutive immune activation driven by tonic TNF-α
Source: EMBO Rep. 2026 Jun 8;27(14):4054–78. doi: 10.1038/s44319-026-00813-5 (PMC13400634; doi:10.1038/s44319-026-00813-5)
Supplement: Supplementary file 14 — Expanded View Figures [file 44319_2026_813_MOESM14_ESM.pdf]

# Expanded View Figures

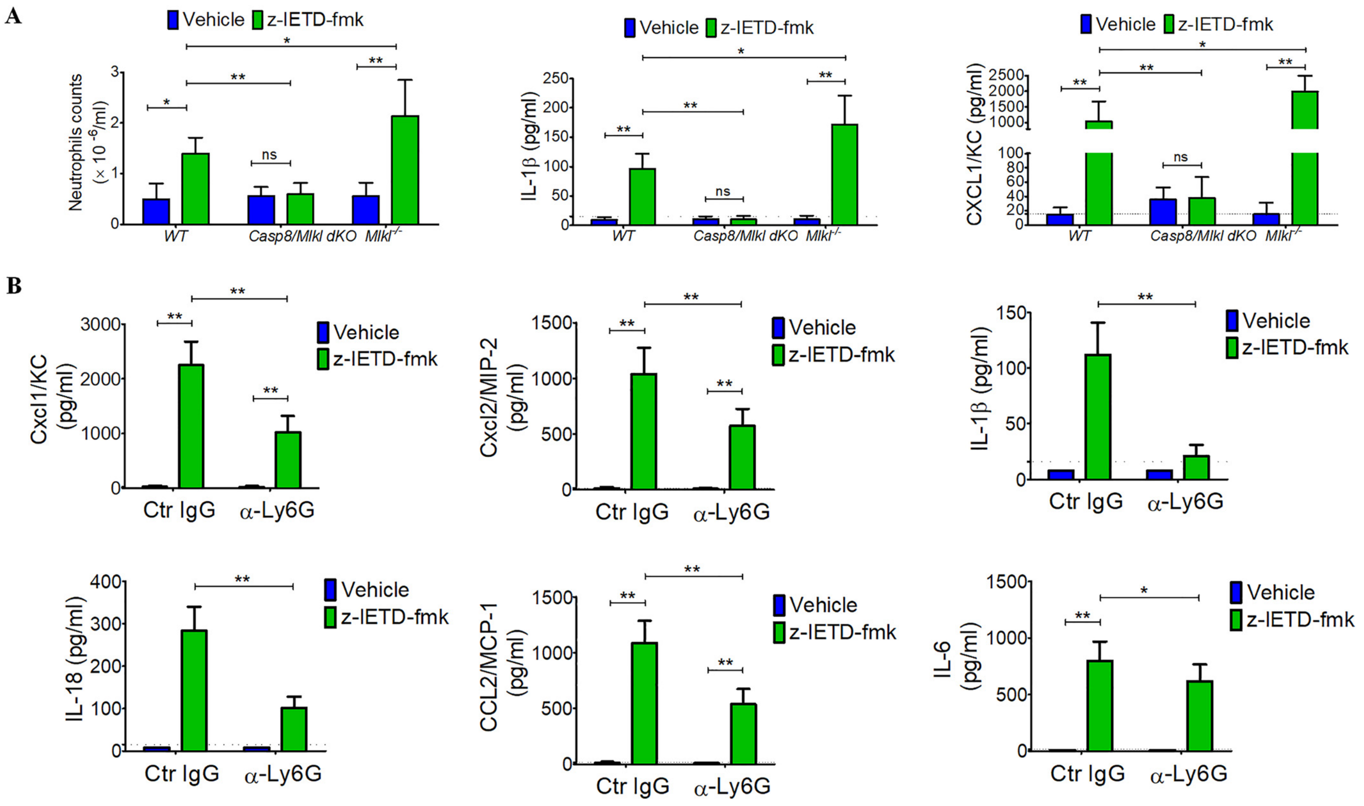


**Figure EV1. z-IETD-fmk-induced cytokine responses are due to specific caspase-8 inhibition and are significantly reduced after neutrophil depletion (related to Figs. 1 and 2).**

(**A**) Mice lacking MLKL (*Mlkl*$^{-/-}$), both MLKL and caspase-8 (*Casp8/MLKL dKO*), or control wild-type (WT) mice were treated with z-IETD (6 mg/kg i.p.) or vehicle. Blood was collected 4 h later to determine neutrophil counts, plasma IL-1β, and plasma CXCL1 concentrations. Columns and bars represent the mean ± SDs of five determinations, each performed using samples from different animals, **P* < 0.05; ***P* < 0.01 by the Mann–Whitney test. (**B**) Mice were pretreated with 100 μg of rat IgG$_{2A}$mAb anti-mouse Ly6G (α-Ly6G) or rat IgG$_{2A}$ isotype control (Ctr IgG) via i.p. injection 24 h prior to z-IETD-fmk or vehicle treatment. Blood was collected 4 h after inhibitor administration for measuring plasma cytokine levels. Columns and bars represent the mean ± SDs of five determinations, each conducted in a different animal. (**A**, **B**) **P* < 0.05; ***P* < 0.01 by the Mann–Whitney test. Source data are available online for this figure.

© The Author(s) 

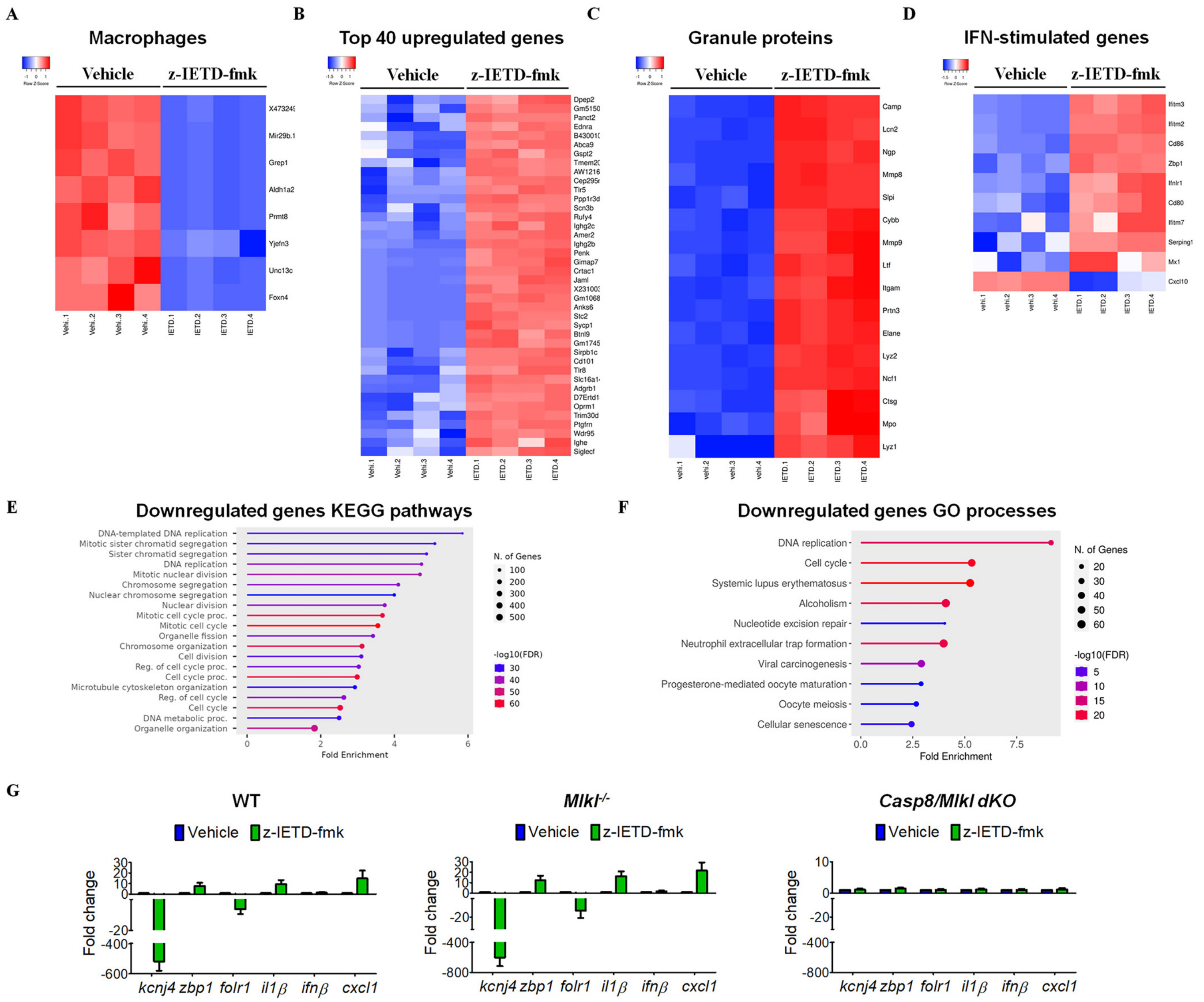


**Figure EV2. Caspase-8 inhibition alters the neutrophil transcriptome (related to Fig. 3).**

(**A**) Heatmap of significantly regulated genes in M-CSF-polarized macrophages exposed in vitro to z-IETD-fmk (50 µM) or vehicle. (**B–D**) Heatmaps of selected gene groups in bone marrow-derived neutrophils treated with z-IETD-fmk or vehicle. (**E**, **F**) Gene expression profiling by Gene Ontology (GO; (**E**) or KEGG pathway (**F**) enrichment analysis of genes downregulated by treatment with z-IETD-fmk in bone marrow-derived neutrophils. FDR, false discovery rate. (**G**) Real-time PCR analysis validation of RNA sequencing data using a panel of selected genes. Bone marrow-derived neutrophils obtained from mice lacking MLKL (*Mlkl*$^{-/-}$), both MLKL and caspase-8 (*Casp8/Mlkl* dKO), or control wild-type (WT) animals were treated with z-IETD-fmk (50 µM for 3 h) or vehicle before RNA extraction and quantitative real-time PCR analysis. Source data are available online for this figure.

 © The Author(s)

A

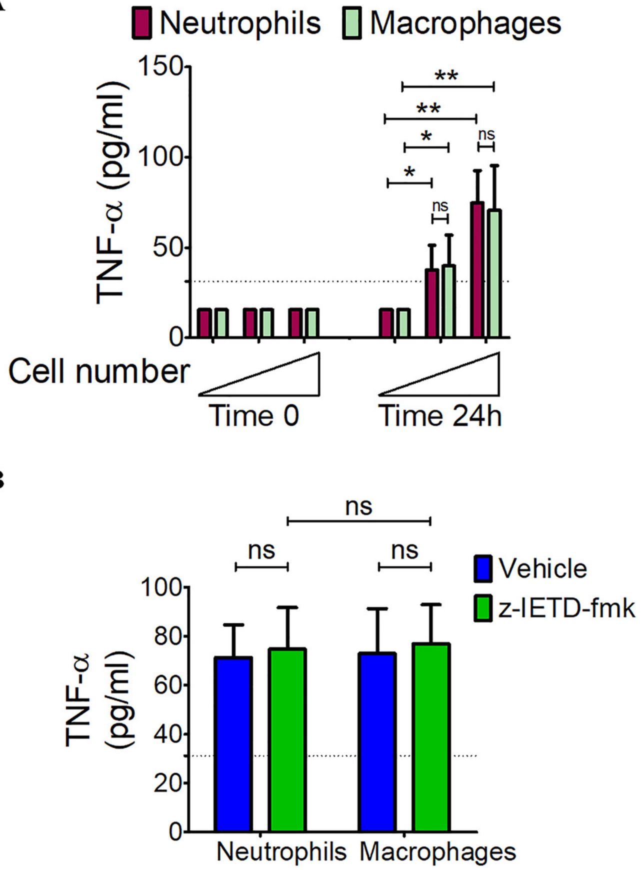


**Figure EV3. Neutrophils spontaneously produce TNF-α (related to Fig. 4).**

(**A**) Neutrophils or macrophages were cultured at increasing densities (2.5, 5, and 15 × $10^6$ cells/mL) for 24 h in the absence of stimuli, and TNF-α concentrations were measured in the culture supernatants. (**B**) Neutrophils or macrophages were cultured at a high cell density (15 × $10^6$ cells/mL) for 24 h in the presence of z-IETD-fmk or vehicle, and TNF-α concentrations were measured in the culture supernatants. Data are means + SDs ($n = 5$ biological replicates). (**A**, **B**) *$P < 0.05$; **$P < 0.01$ by the Mann–Whitney test. ns non-significant. Source data are available online for this figure.

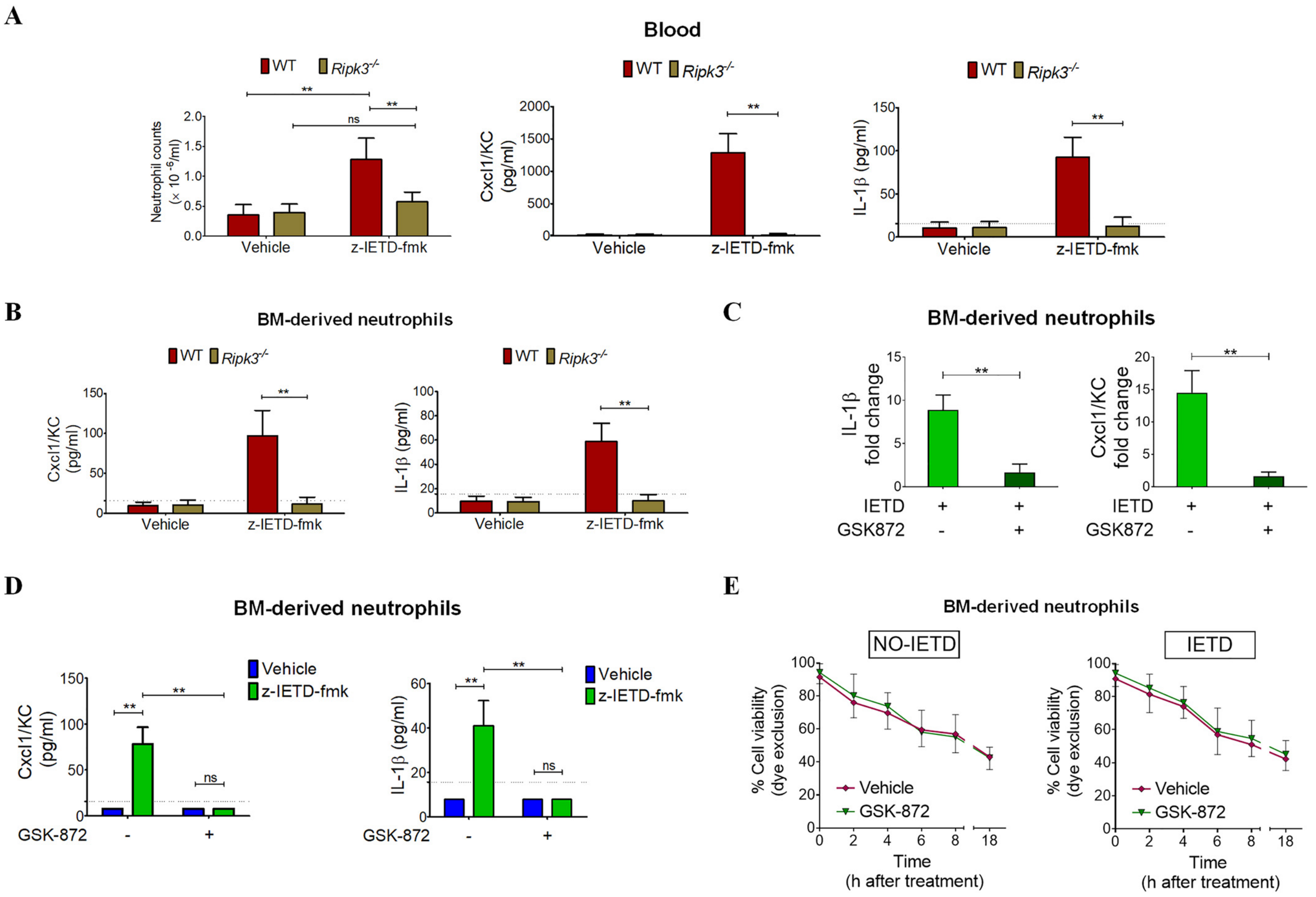


**Figure EV4. IETD-induced neutrophilia and cytokine release require the kinase activity of RIPK3 (related to Fig. 5).**

(**A**) Mice lacking RIPK3 (Ripk3$^{-/-}$) or control wild-type (WT) mice were treated with z-IETD (6 mg/kg i.p.). Blood was collected at 4 h to measure neutrophil counts and plasma cytokine levels by ELISA. (**B**) Bone marrow-derived neutrophils from Ripk3$^{-/-}$ or WT mice were treated overnight with z-IETD-fmk (50 μM) or vehicle. Cytokine concentrations were measured in the culture supernatants. (**C**) Bone marrow-derived neutrophils were pretreated for 1 h with the RIPK3 inhibitor GSK-872 (2 μM) or its vehicle before exposure to z-IETD-fmk (50 μM). Cells were collected for RNA extraction and quantitative real-time PCR analysis of pro-IL1β and Cxcl1 gene transcripts (left and right panels, respectively) at 3 h after exposure to z-IETD-fmk. (**D**) Bone marrow-derived neutrophils were pretreated for 1 h with the RIPK3 inhibitor GSK-872 (2 μM) or its vehicle before exposure to z-IETD-fmk (50 μM). After 24 h, cytokine concentrations were measured in the culture supernatants. (**E**) Bone marrow-derived neutrophils were pretreated for 1 h with the RIPK3 inhibitor GSK-872 (2 μM) or its vehicle before exposure to z-IETD-fmk (50 μM) and collected at the indicated times for determination of viability. (**A**) Columns and bars represent the mean ± SDs of five determinations, each performed using samples from different animals. (**B, C**) Columns bars represent the mean + SDs ($n = 5$ biological replicates). (**D**) Points and bars represent the mean + SDs ($n = 5$ biological replicates). (**A–D**) $^{**}P < 0.01$ by the Mann–Whitney test. ns non-significant. Source data are available online for this figure.

 © The Author(s)

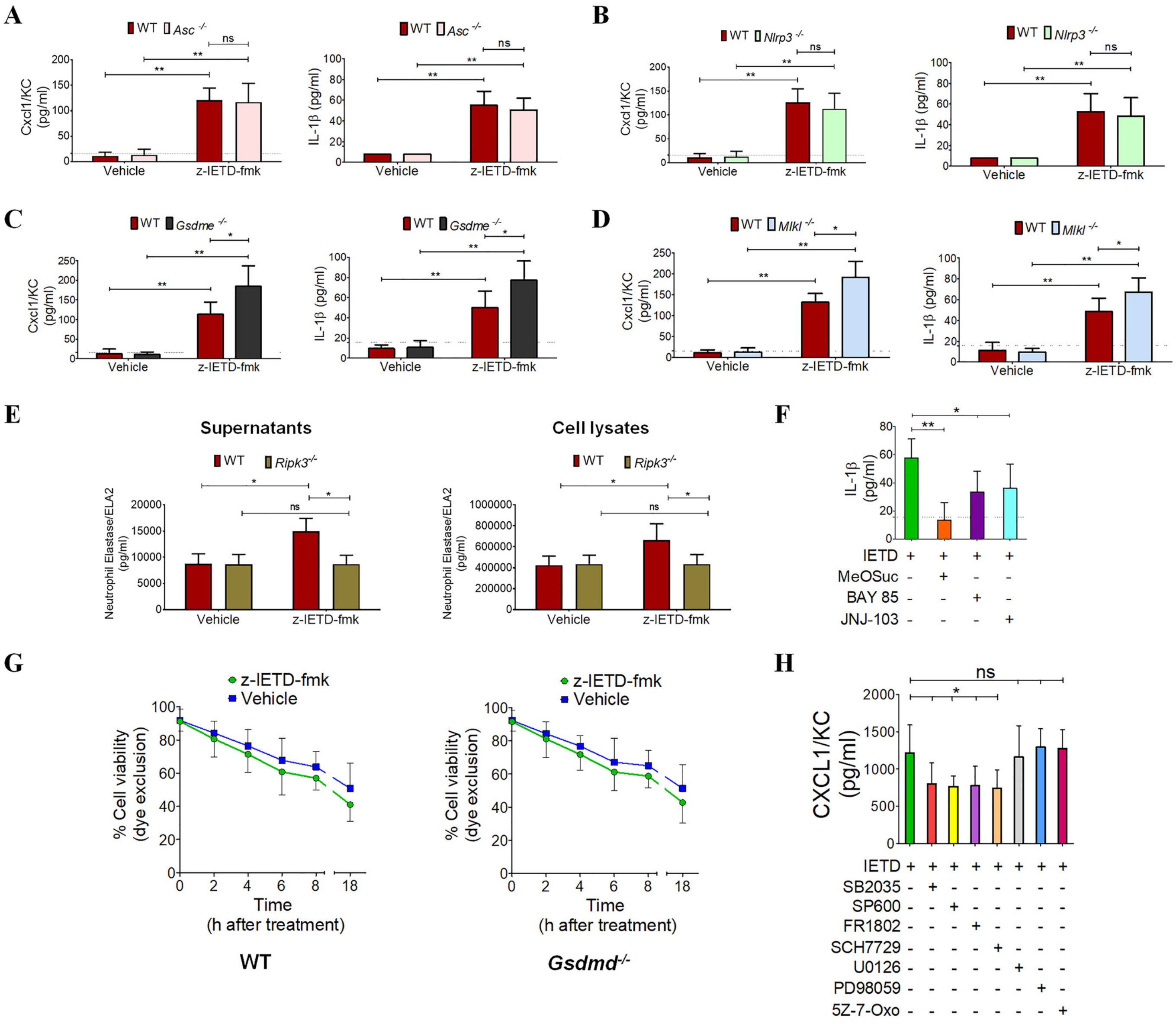


**Figure EV5. IETD-induced IL-1β release is independent of NLRP3, ASC, and cell death, but requires neutrophil serine protease activity (related to Figs. 6 and 7).**

Bone marrow-derived neutrophils were isolated from mice lacking ASC (*Asc*$^{-/-}$; (**A**)), NLRP3 (*Nlrp3*$^{-/-}$; (**B**)), GSDME (*Gsdme*$^{-/-}$; (**C**)), MLKL (*Mlkl*$^{-/-}$; (**D**)), or from control wild-type (WT) mice. Neutrophils were cultured overnight in the presence of z-IETD-fmk or vehicle. CXCL1 and IL-1β levels were measured in culture supernatants by ELISA. (**E**) Effect of z-IETD-fmk (50 µM) on the expression of neutrophil elastase in supernatants and cell lysates of RIPK3$^{-/-}$ and wild-type neutrophils. Cell cultures' supernatants and cell lysates were obtained at 4 h after stimulation. Neutrophil elastase levels were measured by ELISA. (**F**) Effects of neutrophil serine protease inhibitors on IETD-induced IL-1β release in bone marrow neutrophils. Neutrophils were pretreated with the broad neutrophil serine protease inhibitor MeOSuc-AAPV-CMK (MeOSuc), the specific elastase inhibitor BAY 85-8501 (BAY 85), or the cathepsin G inhibitor JNJ-10311795 (JNJ-103) for 2 h before the addition of z-IETD-fmk (50 µM) or vehicle. After overnight culture, IL-1β concentrations were measured in the culture supernatants. (**G**) Spontaneous cell death in bone marrow neutrophils cultured in vitro with z-IETD-fmk (50 µM) or DMSO vehicle. Cells were isolated from wild-type (WT) or GSDMD-deficient (*Gsdmd*$^{-/-}$) mice. (**H**) Bone marrow-derived neutrophils were pretreated for 2 h with the indicated inhibitors of signaling kinases before the addition of z-IETD-fmk (50 µM) or vehicle. After overnight incubation, CXCL1 levels were measured by ELISA in culture supernatants. In all panels, data are means + SDs ($n = 5$ biological replicates). Source data are available online for this figure.

© The Author(s)
