# Supplementary material for: Caspase-8 silences cell death-independent constitutive immune activation driven by tonic TNF-α
Source: EMBO Rep. 2026 Jun 8;27(14):4054–78. doi: 10.1038/s44319-026-00813-5 (PMC13400634; doi:10.1038/s44319-026-00813-5)
Supplement: Supplementary file 1 — Peer Review File [file 44319_2026_813_MOESM1_ESM.pdf]

EMBO reports

Caspase-8 silences cell death-independent constitutive immune activation driven by tonic TNF-α

Giuseppe Teti, Germana Lentini, Francesco Coppolino, Agata Famà, Giuseppe De Gaetano, Federica Grasso, Luigi Fiore, Alessia Berbiglia, Silvia Carnevale, Isabella Venza, Egil Lien, Mario Venza, Sebastien Jaillon, and Concetta Beninati

Corresponding author(s): Giuseppe Teti (gioteti@mac.com)

Review Timeline:

Transfer Date:	11th Oct 25
Editorial Decision:	23rd Oct 25
Revision Received:	31st Jan 26
Editorial Decision:	5th Mar 26
Revision Received:	10th Mar 26
Editorial Decision:	7th May 26
Revision Received:	14th May 26
Accepted:	21st May 26

Editor: Achim Breiling

Transaction Report: This manuscript was transferred to EMBO Reports following peer review at The EMBO Journal.

(Note: With the exception of the correction of typographical or spelling errors that could be a source of ambiguity, letters and reports are not edited. Depending on transfer agreements, referee reports obtained elsewhere may or may not be included in this compilation. Referee reports are anonymous unless the Referee chooses to sign their reports.)

Dear Prof. Teti,

Thank you for transferring your manuscript to EMBO reports. I now went through the manuscript, the referee reports from The EMBO Journal and your preliminary p-b-p-response (revision plan) you sent to us.

The referees, in particular referees #2 and #3, have several comments and concerns, and remain rather critical regarding the work. Some of these points seem to be adequately addressed in your revision plan. In contrast, several referee points (e.g. many of these listed by referee #3 - points 1-24) are not specifically mentioned in your letter. Nevertheless, as your letter suggests that you plan a thorough revision of the study, I have decided to invite a major revision of the manuscript for publication in EMBO reports.

Please take care that all concerns regarding the major conclusions of the study and all technical and experimental limitations mentioned by the referees and points regarding data presentation need to be addressed in a revised manuscript and a full p-b-p-response, also experimentally, when requested by the referees. Please find the referee reports are again below. Please also make sure to cite and discuss all relevant preceding studies, in particular those examining the role of TNFR signaling in mice lacking caspase-8/RIPK1.

Revised manuscripts should be submitted within three months of a request for revision. Please contact me to discuss the revision (also by video chat) if you have further questions or comments regarding the revision, or should you need additional time.

Acceptance of your manuscript will depend on a positive outcome of another round of review at EMBO reports, using the same referees.

When submitting your revised manuscript, please also carefully review the instructions that follow below.

PLEASE NOTE THAT upon resubmission revised manuscripts are subjected to an initial quality control prior to exposition to re-review. Upon failure in the initial quality control, the manuscripts are sent back to the authors, which may lead to delays. Frequent reasons for such a failure are the lack of the data availability section (please see below) and the presence of statistics based on n=2 (the authors are then asked to present scatter plots or provide more data points).

When submitting your revised manuscript, we will require:

1) a .docx formatted version of the final manuscript text (including legends for main figures, EV figures and tables), but without the figures included. Please make sure that changes are highlighted to be clearly visible. Figure legends should be compiled at the end of the manuscript text.

2) individual production quality figure files as .eps, .tif, .jpg (one file per figure), of main figures and EV figures. Please upload these as separate, individual files upon re-submission. Please make sure that all figure panels are called out separately and sequentially in the manuscript text

The Expanded View format, which will be displayed in the main HTML of the paper in a collapsible format, has replaced the Supplementary information. You can submit up to 5 images as Expanded View. Please follow the nomenclature Figure EV1, Figure EV2 etc. The figure legend for these should be included in the main manuscript document file in a section called Expanded View Figure Legends after the main Figure Legends section. Additional Supplementary material should be supplied as a single pdf file labeled Appendix. The Appendix should have page numbers and needs to include a table of content on the first page (with page numbers) and legends for all content. Please follow the nomenclature Appendix Figure Sx, Appendix Table Sx etc. throughout the text, and also label the figures and tables according to this nomenclature.

For more details, please refer to our guide to authors:
http://www.embopress.org/page/journal/14693178/authorguide#manuscriptpreparation

See also our guide for figure preparation:
http://wol-prod-cdn.literatumonline.com/pb-assets/embo-site/EMBOPress_Figure_Guidelines_061115-1561436025777.pdf

Moreover, please consult our guidelines for figure legend preparation:
https://www.embopress.org/page/journal/14693178/authorguide#figureformat

3) a .docx formatted letter INCLUDING the reviewers' reports and your detailed point-by-point responses to their comments. As part of the EMBO Press transparent editorial process, the point-by-point response is part of the Review Process File (RPF), which will be published alongside your paper.

4) a complete author checklist, which you can download from our author guidelines (https://www.embopress.org/page/journal/14693178/authorguide). Please insert page numbers in the checklist to indicate where the requested information can be found in the manuscript. The completed author checklist will also be part of the RPF.

Please also follow our guidelines for the use of living organisms, and the respective reporting guidelines: http://www.embopress.org/page/journal/14693178/authorguide#livingorganisms

5) that primary datasets produced in this study (e.g. RNA-seq, ChIP-seq and array data) are deposited in an appropriate public database. This is now mandatory (like the COI statement). If no primary datasets have been deposited in any database, please state this in this section (e.g. 'No primary datasets have been generated and deposited').

See also: http://embor.embopress.org/authorguide#datadeposition

Please remember to provide a reviewer password if the datasets are not yet public.

The accession numbers and database should be listed in a formal "Data Availability" section (placed after Materials & Methods) that follows the model below. Please note that the Data Availability Section is restricted to new primary data that are part of this study.

Data availability

The datasets produced in this study are available in the following databases:

- RNA-Seq data: Gene Expression Omnibus GSE46843 (https://www.ncbi.nlm.nih.gov/geo/query/acc.cgi?acc=GSE46843)
- [data type]: [name of the resource] [accession number/identifier/doi] ([URL or identifiers.org/DATABASE:ACCESSION])

*** Note - All links should resolve to a page where the data can be accessed. ***

Moreover, I have these editorial requests:

6) We now request the publication of original source data with the aim of making primary data more accessible and transparent to the reader. You will receive a separate email with instructions for providing source data with your revised manuscript, including information how to upload and organize the files.

7) Our journal encourages inclusion of *data citations in the reference list* to directly cite datasets that were re-used and obtained from public databases. Data citations in the article text are distinct from normal bibliographical citations and should directly link to the database records from which the data can be accessed. In the main text, data citations are formatted as follows: "Data ref: Smith et al, 2001" or "Data ref: NCBI Sequence Read Archive PRJNA342805, 2017". In the Reference list, data citations must be labeled with "[DATASET]". A data reference must provide the database name, accession number/identifiers and a resolvable link to the landing page from which the data can be accessed at the end of the reference. Further instructions are available at: http://www.embopress.org/page/journal/14693178/authorguide#referencesformat

8) Regarding data quantification and statistics, please make sure that the number "n" for how many independent experiments were performed, their nature (biological versus technical replicates), the bars and error bars (e.g. SEM, SD) and the test used to calculate p-values is indicated in the respective figure legends (also for EV and Appendix figures). Please also check that all the p-values are explained in the legend, and that these fit to those shown in the figure. Please provide statistical testing where applicable. Please avoid the phrase 'independent experiment' but clearly state if these were biological or technical replicates. Please also indicate (e.g. with n.s.) if testing was performed, but the differences are not significant. In case n=2, please show the data as separate datapoints without error bars and statistics. See also: http://www.embopress.org/page/journal/14693178/authorguide#statisticalanalysis

If n<5, please show single datapoints for diagrams.

9) Please add scale bars of similar style and thickness to microscopic images, using clearly visible black or white bars (depending on the background). Please place these in the lower right corner of the images themselves. Please do not write on or near the bars in the image but define the size in the respective figure legend.

10) Please also note our reference format: http://www.embopress.org/page/journal/14693178/authorguide#referencesformat

11) We updated our journal's competing interests policy in January 2022 and request authors to consider both actual and perceived competing interests. Please review the policy https://www.embopress.org/competing-interests and update your competing interests if necessary. Please name this section 'Disclosure and Competing Interests Statement' and put it after the Acknowledgements section.

12) We now use CRediT to specify the contributions of each author in the journal submission system. CRediT replaces the author contribution section. Please use the free text box to provide more detailed descriptions and do NOT provide your final manuscript text file with an author contributions section. See also our guide to authors: https://www.embopress.org/page/journal/14693178/authorguide#authorshipguidelines

13) All Materials and Methods need to be described in the main text using our 'Structured Methods' format, which is required for all research articles. According to this format, the Methods section should include a Reagents and Tools Table (listing key reagents, experimental models, software, and relevant equipment and including their sources and relevant identifiers), uploaded as separate file, and a Methods section in which we encourage the authors to describe their methods using a step-by-step protocol format with bullet points, to facilitate the adoption of the methodologies across labs. More information on how to adhere to this format as well as downloadable templates (.doc) for the Reagents and Tools Table can be found in our author guidelines (section 'Structured Methods'):

https://www.embopress.org/page/journal/14693178/authorguide#manuscriptpreparation

14) Please reduce the number of keywords to five and order the manuscript sections like this, using these names:
Title page - Abstract (not more than 175 words) - Keywords - Introduction - Results - Discussion - Methods - Data availability section - Acknowledgements (please include here also the funding information) - Disclosure and Competing Interests Statement - References - Figure legends - Expanded View Figure legends

15) Please make sure that all the funding information is also entered into the online submission system and that it is complete and similar to the one in the acknowledgement section of the manuscript text file.

We would also welcome the submission of cover suggestions, or motifs to be used by our Graphics Illustrator in designing a cover.

As part of the EMBO publication's Transparent Editorial Process, EMBO reports publishes online a Review Process File (RPF) to accompany accepted manuscripts. This File will be published in conjunction with your paper and will include the referee reports, your point-by-point response and all pertinent correspondence relating to the manuscript.

You are able to opt out of this by letting the editorial office know (emboreports@embo.org). If you do opt out, the Review Process File link will point to the following statement: "No Review Process File is available with this article, as the authors have chosen not to make the review process public in this case."

I look forward to seeing a revised version of your manuscript when it is ready. Please let me know if you have questions or comments regarding the revision.

Yours sincerely,

Achim Breiling
Senior Editor
EMBO Reports

Referee #1:

The work uncovers a previously unrecognized, neutrophil-specific inflammatory pathway regulated by caspase-8. It reveals that caspase-8 functions beyond apoptosis/necrosis control, acting as a central regulator of immune homeostasis. Furthermore, authors demonstrated that neutrophils can drive IL-1β secretion without classical inflammasome machinery, instead relying on gasdermin D and serine proteases. The study suggests potential applications in infection and immunity.

The manuscript presents well performed experiments, that in several cases, were confirmed using two different approaches (Ex. KO mice and inhibitors) and combining in vitro and in vivo work and state-of-the art techniques. Results are well described and Figures are clearly designed.

Minor revisions:

Line 134 : revise as 1 hour (remove "s").

Line 403: In vivo caspase-8 inhibition: could you provide justification of the doses used for inhibition or a reference to support their selection.

Referee #2:

This manuscript examines the consequences of pharmacological Caspase-8 inhibition on inflammatory responses in vivo and roles for TNF, GSDMD, RIPK1 kinase activity, and MLKL. The manuscript provides convincing evidence to support a role for Caspase-8 inhibition inducing an inflammatory response, via pathways very well described by numerous investigators studying Caspase-8 deficiency and regulation of inflammatory responses via systemic necroptotic cell death, and components of the ripoptosome such as RIPK1 and RIPK3.

Additional experiments assessing caspase-3/7 activation downstream of caspase-8 following z-IETD-fmk treatment in MLKL KO samples would further strengthen the study and address previously described mechanisms for substrate availability in the activation of Casp8 and MLKL after RIP1/RIP3 activation.

The major weakness is the authors' previous publication in Cell Reports Medicine (2023, PMID:37390829) which established many of the findings also reported in this manuscript with complementary model systems or identical methods. Several findings are also reported in the 2023 study including neutrophil counts in PC (Fig2F 2023; Fig2D manuscript), cytokine levels (Fig2F, 2023; Fig1D, EMBO), cytokine production (Fig3C, 2023; Fig4E, manuscript), and IL-1b western (Fig5A, 2023; Fig6C, EMBO). These overlapping reports significantly reduce the novelty of the current manuscript.

The simplistic introduction of TNF effects on neutrophils (ROS production, degranulation, and NET release) lacks a nuanced view of how TNF may be impacting TNF signal transduction or impacting Casp8/MLKL pathways in neutrophils.

The statement on line 116-118 is not supported by statistics - authors should display fold change/statistics on IgG control vs α-CXCR2 treated Csf3r-/- and WT mice. It appears that anti-cxcr2 Ab do abrogate neutrophilia in both instances.

For lines 131-133, the authors suggest that CLP is an excellent model of human sepsis. Given the research using CLP models and the multitude of effective interventions in the mouse, the authors should explicitly state how such research has translated into effective treatments for human sepsis. If this is not the case, perhaps it cannot be considered as a good model of human sepsis.

In line 149, 'almost completely unable' is perhaps an overstatement when referencing IL-1b and IL-18 responses.

Information on statistical tests performed is required in figure legends for S1, S6, and S7.

Referee #3:

This manuscript reports that IETD treatment results in cell death-independent TNF-RIPK1-RIPK3 induced cytokine transcription and their activation of neutrophil elastase/cathepsins to trigger GSDMD and IL-1beta. However, key data to support these conclusions is, for the most, lacking.

First, the authors never show that IETD activates neutrophil elastase/cathepsins (data is based on an inhibitor studies only).

Second, they never show that loss of elastase/cathepsin activity reduces GSDMD and IL-1beta activation (i.e. processing) and activated (p17) IL-1b release.

Third, the authors never examine if RIPK1 D138N and RIPK3 KO (and MLKL KO control) neutrophils have reduced IETD-driven cathepsin/elastase activity and consequently reduced GSDMD/IL-1beta activation (i.e. processing to their bioactive fragments).

Fourth, the authors do not acknowledge that Z-IETD-fmk exhibits significant cross reactivity towards other caspases, the fmk group has well documented off-target effects and fmk-based inhibitors can also cross-react with lysosomal proteases. For transparency, the authors should not hide these caveats. I also don't think the authors can claim the lack of effect of IETD in caspase-8/MLKL DKO mice as evidence that it is acting "on target" as caspase-8/MLKL loss profoundly impacts immune cell transcriptional programs - so these mice fail to respond normally to most inflammatory stimuli, including IL-1 transcription.

Fifth, as detailed below, I do not believe the authors can claim their observations occur independent of cell death as the neutrophils die with and without IETD treatment and exhibit markers of both necroptosis (P-RIPK3) and pyroptosis (GSDMD cleavage).

Finally, the novelty of the manuscript is diminished due to their previous study in Cell Reports Medicine (https://doi.org/10.1016/j.xcrm.2023.101098) where they showed that IETD activated RIPK1 and RIPK3 to drive gene transcription of cytokines such as IL-1 and also enabled increased anti-pathogen defence. This previous study also documented that caspase-1/11 DKO mice exhibited significantly reduced IL-1b and also abrogated neutrophilia upon IETD injection - which

contradicts key conclusions of the current paper.

Specific comments:
1. The number of times each experiment, including the in vivo experiments, were repeated and results reproduced needs to be clearly stated in the figure legends. As currently written, it is unclear how reproducible the data was across independent experiments.

2. The authors find that z-IETD-fmk increased responses in MLKL KO mice (Fig. S1), which is an important observation. For example, is MLKL loss prolonging the survival of caspase-8 inhibited cells, thereby enabling increased cytokine production and subsequent neutrophil counts?

3. Related to point 2 - caspase-8 inhibition would be expected to trigger necroptosis in susceptible cell types - do the authors observe any evidence of this other than P-RIPK3 detection and the indirect data suggesting this is the case (presented in Fig. S1)?

4. The cecal ligation punction data (Fig. 2) should be repeated in casp8/MLKL DKO and MLKL KO mice to define if the impact observed is a result of MLKL being triggered by caspase-8 inhibition, or otherwise.

5. GSK872 can induce apoptosis via causing a conformational change in RIPK3, so the authors statement, line 222, that "the kinase function of RIPK3, rather than its scaffold functions, is required for neutrophil responses to IETD" may not be the case.

6. The authors need to analyse the caspase-1/11 deficient mice as they have done in Fig. 6A, B, S6A,SB) as the western blots are not clear cut and lack appropriate positive controls to define if caspase-1 is being activated or not (particularly as both IL-1 and caspase-1 activated/processed fragments are rapidly released from cells and therefore levels in the cell lysates, which the authors analyze, are not very informative).

7. Figure 6F and Fig. S1A - the data do not agree with other. In one data set neutrophil counts are significantly increased in MLKL KO mice, but in the other data set there is no difference. This speaks to the importance of defining in the figure legends how many times the experiments were reproduced.

8. Fig. 7C. Contrary to the authors assertions, BAY85 and MeoCuc, do not reduce GSDMD p23 levels (i.e. this product looks similar to IETD control treated cells). Infact these inhibitors result in an increase in p30 levels - which represents the pore-forming fragment of GSDMD. One would therefore predict these cells to exhibit increased rates of pyroptotic death.

9. Fig. 7D. The differences reported are very small - ~ 80 pg/ml in control vs 50-60 pg/ml in inhibitor treatments. These data also do not distinguish between inhibitor impacts on IL-1beta gene transcription vs IL-1beta processing and activation, which is critical for its interpretation and the authors conclusions. As the neutrophils spontaneously die over time (Fig. S7B) they could simply be releasing precursor (inactive) IL-1beta. The examination of GSDMD/IL-1beta activation (i.e. processing in cell lysates and supernatants) is required in these treatment conditions.

10. Given the authors conclude that IETD treatment results in RIPK1-RIPK3 activation to allow triggering of GSDMD and IL-1beta release via serine proteases, they need to examine GSDMD processing/activation, IL-1beta activation (i.e. p17 levels in neutrophil cell lysates and supernatants) and elastase/cathepsin activity using TNFR1 KO, RIPK1 kinase dead, RIPK3 KO, and control - MLKL KO, caspase-1/11 DKO - neutrophils. This is important as the authors previous Cell Reports Medicine paper showed theat the caspase-1/11 DKO mice injected with IETD showed the same effects to the GSDMD KO mice used in this paper (i.e. reduced IL-1beta levels and also neutrophil levels that were almost back to untreated levels).

11. Fig. S7B. WT and GSDMD KO death kinetics - mean and error - looks very similar between the WT and GSDMD KO mice. For example, one would be hard pressed to find differences if these graphs were overlayed, particularly for the vehicle WT and vehicle GSDMD KO data.

12. The authors conclude that GSDMD is not causing death following IETD treatment. However, neutrophils are clearly dying (Fig. S7B) and adding IETD may have simply converted neutrophil killing from one type of cell death (eg. apoptosis) into another type of cell death (e.g. pyroptosis), so to conclude that GSDMD does not cause pyroptosis under these conditions is premature.

13. The authors conclude (line 306) that the effects they observe occur "without evidence of necroptosis, pyroptosis or other forms of cell death". 1st the authors observe P-RIPK3 upon IETD treatment, which is a marker o necroptosis. 2nd they observe GSDMD activation/processing, a marker of pyroptosis. 3rd the neutrophils are dieing over time with and without IETD. To claim all IETD-induced neutrophila and elevated cytokines is therefore cell death independent is not warranted.

14. A model schematic would be informative.

**Manuscript #**	EMBOR-2025-62947-T.
**Title**	Caspase-8 silences cell death-independent constitutive immune activation driven by tonic TNF-α
**Corresponding Author**	Giuseppe Teti

To the Editors of EMBO Reports

Referee's comments and point by point response

Referee's comments

Referee #1:

The work uncovers a previously unrecognized, neutrophil-specific inflammatory pathway regulated by caspase-8. It reveals that caspase-8 functions beyond apoptosis/necrosis control, acting as a central regulator of immune homeostasis. Furthermore, authors demonstrated that neutrophils can drive IL-1β secretion without classical inflammasome machinery, instead relying on gasdermin D and serine proteases. The study suggests potential applications in infection and immunity.

The manuscript presents well performed experiments, that in several cases, were confirmed using two different approaches (Ex. KO mice and inhibitors) and combining in vitro and in vivo work and state-of-the art techniques. Results are well described and Figures are clearly designed.

Minor revisions:

Line 134 : revise as 1 hour (remove "s").

Line 403: In vivo caspase-8 inhibition: could you provide justification of the doses used for inhibition or a reference to support their selection.

Referee #2:

This manuscript examines the consequences of pharmacological Caspase-8 inhibition on inflammatory responses in vivo and roles for TNF, GSDMD, RIPK1 kinase activity, and MLKL. The manuscript provides convincing evidence to support a role for Caspase-8 inhibition inducing an inflammatory response, via pathways very well described by numerous investigators studying Caspase-8 deficiency and regulation of inflammatory responses via systemic necroptotic cell death, and components of the ripoptosome such as RIPK1 and RIPK3.

Additional experiments assessing caspase-3/7 activation downstream of caspase-8 following z-IETD-fmk treatment in MLKL KO samples would further strengthen the study and address previously described mechanisms for substrate availability in the activation of Casp8 and MLKL after RIP1/RIP3 activation.

The major weakness is the authors' previous publication in Cell Reports Medicine (2023, PMID:37390829) which established many of the findings also reported in this manuscript with complementary model systems or identical methods. Several findings are also reported in the 2023 study including neutrophil counts in PC (Fig2F 2023; Fig2D manuscript), cytokine levels (Fig2F, 2023; Fig1D, EMBO), cytokine production (Fig3C, 2023; Fig4E, manuscript), and IL-1b western (Fig5A, 2023; Fig6C, EMBO). These overlapping reports significantly reduce the novelty of the current manuscript.

The simplistic introduction of TNF effects on neutrophils (ROS production, degranulation, and NET release) lacks a nuanced view of how TNF may be impacting TNF signal transduction or impacting Casp8/MLKL pathways in neutrophils.

The statement on line 116-118 is not supported by statistics - authors should display fold change/statistics on IgG control vs α-CXCR2 treated Csf3r-/- and WT mice. It appears that anti-cxcr2 Ab do abrogate neutrophilia in both instances.

For lines 131-133, the authors suggest that CLP is an excellent model of human sepsis. Given the research using CLP models and the multitude of effective interventions in the mouse, the authors should explicitly state how such research has translated into effective treatments for human sepsis. If this is not the case, perhaps it cannot be considered as a good model of human sepsis.

In line 149, 'almost completely unable' is perhaps an overstatement when referencing IL-1b and IL-18 responses.

Information on statistical tests performed is required in figure legends for S1, S6, and S7.

Referee #3:

This manuscript reports that IETD treatment results in cell death-independent TNF-RIPK1-RIPK3 induced cytokine transcription and their activation of neutrophil elastase/cathepsins to trigger GSDMD and IL-1beta. However, key data to support these conclusions is, for the most, lacking.

First, the authors never show that IETD activates neutrophil elastase/cathepsins (data is based on an inhibitor studies only).

Second, they never show that loss of elastase/cathepsin activity reduces GSDMD and IL-1beta activation (i.e. processing) and activated (p17) IL-1b release.

Third, the authors never examine if RIPK1 D138N and RIPK3 KO (and MLKL KO control) neutrophils have reduced IETD-driven cathepsin/elastase activity and consequently reduced GSDMD/IL-1beta activation (i.e. processing to their bioactive fragments).

Fourth, the authors do not acknowledge that Z-IETD-fmk exhibits significant cross reactivity towards other caspases, the fmk group has well documented off-target effects and fmk-based inhibitors can also cross-react with lysosomal proteases. For transparency, the authors should not hide these caveats. I also don't think the authors can claim the lack of effect of IETD in caspase-8/MLKL DKO mice as evidence that it is acting "on target" as caspase-8/MLKL loss profoundly impacts immune cell transcriptional programs - so these mice fail to respond normally to most inflammatory stimuli, including IL-1 transcription.

Fifth, as detailed below, I do not believe the authors can claim their observations occur independent of cell death as the neutrophils die with and without IETD treatment and exhibit markers of both necroptosis (P-RIPK3) and pyroptosis (GSDMD cleavage).

Finally, the novelty of the manuscript is diminished due to their previous study in Cell Reports Medicine (https://doi.org/10.1016/j.xcrm.2023.101098) where they showed that IETD activated RIPK1 and RIPK3 to drive gene transcription of cytokines such as IL-1 and also enabled increased anti-pathogen defence. This previous study also documented that caspase-1/11 DKO mice exhibited significantly reduced IL-1b and also abrogated neutrophilia upon IETD injection - which contradicts key conclusions of the current paper.

Specific comments:
1. The number of times each experiment, including the in vivo experiments, were repeated and results reproduced needs to be clearly stated in the figure legends. As currently written, it is unclear how reproducible the data was across independent experiments.

2. The authors find that z-IETD-fmk increased responses in MLKL KO mice (Fig. S1), which is an important observation. For example, is MLKL loss prolonging the survival of caspase-8 inhibited cells, thereby enabling increased cytokine production and subsequent neutrophil counts?

3. Related to point 2 - caspase-8 inhibition would be expected to trigger necroptosis in susceptible cell types - do the authors observe any evidence of this other than P-RIPK3 detection and the indirect data suggesting this is the case (presented in Fig. S1)?

4. The cecal ligation punction data (Fig. 2) should be repeated in casp8/MLKL DKO and MLKL KO mice to define if the impact observed is a result of MLKL being triggered by caspase-8 inhibition, or otherwise.

5. GSK872 can induce apoptosis via causing a conformational change in RIPK3, so the authors statement, line 222, that "the kinase function of RIPK3, rather than its scaffold functions, is required for neutrophil responses to IETD" may not be the case.

6. The authors need to analyse the caspase-1/11 deficient mice as they have done in Fig. 6A, B, S6A,SB) as the western blots are not clear cut and lack appropriate positive controls to define if caspase-1 is being activated or not (particularly as both IL-1 and caspase-1 activated/processed fragments are rapidly released from cells and therefore levels in the cell lysates, which the authors analyze, are not very informative).

7. Figure 6F and Fig. S1A - the data do not agree with other. In one data set neutrophil counts are significantly increased in MLKL KO mice, but in the other data set there is no difference. This speaks to the importance of defining in the figure legends how many times the experiments were reproduced.

8. Fig. 7C. Contrary to the authors assertions, BAY85 and MeoCuc, do not reduce GSDMD p23 levels (i.e. this product looks similar to IETD control treated cells). Infact these inhibitors result in an increase in p30 levels - which represents the pore-forming fragment of GSDMD. One would therefore predict these cells to exhibit increased rates of pyroptotic

death.

9. Fig. 7D. The differences reported are very small - ~ 80 pg/ml in control vs 50-60 pg/ml in inhibitor treatments. These data also do not distinguish between inhibitor impacts on IL-1beta gene transcription vs IL-1beta processing and activation, which is critical for its interpretation and the authors conclusions. As the neutrophils spontaneously die over time (Fig. S7B) they could simply be releasing precursor (inactive) IL-1beta. The examination of GSDMD/IL-1beta activation (i.e. processing in cell lysates and supernatants) is required in these treatment conditions.

10. Given the authors conclude that IETD treatment results in RIPK1-RIPK3 activation to allow triggering of GSDMD and IL-1beta release via serine proteases, they need to examine GSDMD processing/activation, IL-1beta activation (i.e. p17 levels in neutrophil cell lysates and supernatants) and elastase/cathepsin activity using TNFR1 KO, RIPK1 kinase dead, RIPK3 KO, and control - MLKL KO, caspase-1/11 DKO - neutrophils. This is important as the authors previous Cell Reports Medicine paper showed theat the caspase-1/11 DKO mice injected with IETD showed the same effects to the GSDMD KO mice used in this paper (i.e. reduced IL-1beta levels and also neutrophil levels that were almost back to untreated levels).

11. Fig. S7B. WT and GSDMD KO death kinetics - mean and error - looks very similar between the WT and GSDMD KO mice. For example, one would be hard pressed to find differences if these graphs were overlayed, particularly for the vehicle WT and vehicle GSDMD KO data.

12. The authors conclude that GSDMD is not causing death following IETD treatment. However, neutrophils are clearly dying (Fig. S7B) and adding IETD may have simply converted neutrophil killing from one type of cell death (eg. apoptosis) into another type of cell death (e.g. pyroptosis), so to conclude that GSDMD does not cause pyroptosis under these conditions is premature.

13. The authors conclude (line 306) that the effects they observe occur "without evidence of necroptosis, pyroptosis or other forms of cell death". 1st the authors observe P-RIPK3 upon IETD treatment, which is a marker o necroptosis. 2nd they observe GSDMD activation/processing, a marker of pyroptosis. 3rd the neutrophils are dieing over time with and without IETD. To claim all IETD-induced neutrophila and elevated cytokines is therefore cell death independent is not warranted.

14. A model schematic would be informative.

Point-by-Point Response to the reviewers' comments

For ease of consultation, we have copied here the Reviewers' comments (*italic*) before our response (regular font). We believe to have responded to all comments.

Reviewer #1

Comment. *The work uncovers a previously unrecognized, neutrophil-specific inflammatory pathway regulated by caspase-8. It reveals that caspase-8 functions beyond apoptosis/necrosis control, acting as a central regulator of immune homeostasis. Furthermore, authors demonstrated that neutrophils can drive IL-1β secretion without classical inflammasome machinery, instead relying on gasdermin D and serine proteases. The study suggests potential applications in infection and immunity.*

The manuscript presents well-performed experiments that, in several cases, were confirmed using two different approaches (e.g., KO mice and inhibitors) and combining in vitro and in vivo work and state-of-the-art techniques. Results are well described and Figures are clearly designed.

Minor revisions: Line 134 : revise as 1 hour (remove "s").
Line 403: In vivo caspase-8 inhibition: could you provide justification of the doses used for inhibition or a reference to support their selection.

Response. We thank the reviewer for his/her kind appreciation of our work and the encouraging feedback. The minor revisions suggested have been incorporated into the revised manuscript. In particular, we have added a sentence (lines 412-414 of the revised manuscript) to explain that the IETD doses were selected based on previous studies (ref. 11) showing that these doses were effective at inhibiting caspase-8 activity and at the same time had no off-target effects.

Reviewer #2

For clarity, our response to this reviewer is broken down into several sections

Role of necroptosis

Comment. *The manuscript provides convincing evidence to support a role for Caspase-8 inhibition inducing an inflammatory response via pathways very well described by numerous investigators studying Caspase-8 deficiency and regulation of inflammatory responses via systemic necroptotic cell death, and components of the ripoptosome such as RIPK1 and RIPK3. Additional experiments assessing caspase-3/7 activation downstream of caspase-8 following z-IETD-fmk treatment in MLKL KO samples would further strengthen the study and address previously described mechanisms for substrate availability in the activation of Casp8 and MLKL after RIP1/RIP3 activation.*

Response. We thank the reviewer for this comment; however we respectfully disagree with some of its statements. From the outset, the reviewer appears to attribute the inflammatory changes observed following caspase-8 inhibition are due to necroptosis, a well-established form of programmed cell death that can be triggered or enhanced in the absence or inactivation of caspase-8. The reviewer further suggests exploring the possibility that IETD induces apoptosis through Casp3/7 activation when necroptosis is

blocked by the absence of MLKL. Collectively, these comments imply that the reviewer interprets the changes we describe as being driven by programmed cell death, either necroptosis or apoptosis. However, such an interpretation would directly contradict our central conclusion that the changes we described are independent of necroptosis, apoptosis or other forms of cell death. We note that the reviewer does not provide specific evidence to support his/her claims and overlooks substantial data from our current and prior studies (Cell Reports Medicine; 2023, PMID:37390829, ref. 11). We respectfully point out that our conclusion—that the observed changes are independent of necroptosis or other forms of cell death—is supported by multiple independent lines of evidence:

- Temporal resolution: The transcriptomic changes in neutrophils appear within 1–3 hours of caspase-8 inhibition (Fig 3 of the present manuscript). At these early time points, the cells show no evidence of cell death, as measured by dye exclusion (Fig. S4A of ref. 11, our 2023 paper and Fig. S7B of the present study). Indeed, neutrophils in culture begin to die spontaneously only after several hours, and to the same extent in cell treated or untreated with the caspase inhibitor IETD.
- Genetic data: None of the effects of the IETD caspase inhibitor, as used in our experimental settings, is prevented or diminished in MLKL-deficient mice (Fig. S1 of this manuscript and Fig 4B of the 2023 paper). Because MLKL is indispensable for, and serves as the executioner of, necroptosis, these findings rule out necroptotic cell death as the underlying mechanism. Induction of apoptosis by IETD treatment in the absence of MLKL was also ruled out by our previous data (Fig. S4A of the Cell Reports Medicine paper; 2023, PMID:37390829)

Taken together, these data strongly support our conclusion that the proinflammatory transcriptional response to caspase-8 inhibition is not a consequence of necroptosis or any other form of cell death.

Novelty

Comment. *The major weakness is the authors' previous publication in Cell Reports Medicine (2023, PMID:37390829) which established many of the findings also reported in this manuscript with complementary model systems or identical methods. Several findings are also reported in the 2023 study including neutrophil counts in PC (Fig2F 2023; Fig2D manuscript), cytokine levels (Fig2F, 2023; Fig1D, EMBO), cytokine production (Fig3C, 2023; Fig4E, manuscript), and IL-1b western (Fig5A, 2023; Fig6C, EMBO). These overlapping reports significantly reduce the novelty of the current manuscript.*

Response. We respectfully disagree with the reviewer's assessment regarding the novelty of the manuscript.

In fact, the current manuscript provides entirely novel (and in our opinion important) findings that had not been reported in our previous study or in studies by others, including:

- Homeostatic (tonic) TNF-α production by neutrophils (a phenomenon not previously identified by us or others) and its indispensable role in the induction of marked increases in blood neutrophil counts (neutrophilia) triggered by caspase-8 inhibition in the absence of cell death. We would like to stress again that this neutrophilic response to caspase 8 inhibition is described for the first time in the present study and was not previously reported by us or by others.

- The requirement of autocrionous activation of TNF receptor 1 for such inflammatory response
- Global control of neutrophil function by caspase-8, including changes in NETosis, DNA repair, IFN response, and granule protein gene expression, as revealed by transcriptomic changes in over one-fourth of the neutrophil genome upon caspase-8 inhibition
- The involvement of gasdermin D and its processing by serine proteases in IL-1β release, an event that we show to be independent of canonical inflammasome pathways.

Second, although both studies concern responses to caspase-8 inhibition, the biological phenomena and scientific focus of two are fundamentally different:

- Our 2023 publication focused on local effects of caspase-8 inhibition i.e. neutrophil recruitment at the site of inhibitor injection.
- The current manuscript investigates systemic consequences of caspase-8 inhibition, namely, circulating cytokine release and mobilization of neutrophils from hematopoietic tissues, resulting in marked increases in blood neutrophil counts.

The reviewer's argument that similar findings are repeated is based on superficial similarities in figure appearance, but a closer examination reveals these represent distinct experimental contexts and biological conclusions:

- Fig. 2F (2023) and Fig. 2D (present study) both involve peritoneal neutrophil counts, but the former refers to naïve mice treated with caspase-8 inhibitors alone, while the latter analyzes mice subjected to cecal ligation and puncture (CLP), a model involving polymicrobial peritonitis. The CLP model was not included in the 2023 paper.
- Fig. 2F (2023) vs. Fig. 1D (present study): While both show cytokine levels, they do so in different compartments (peritoneal fluid vs. plasma) and concern different biological responses (local inflammation vs. systemic neutrophilia caused by mobilization from hemopoietic tissues).
- Fig. 5A (2023) and Fig. 6C (previous version of present study) both show IL-1β processing in peritoneal exudates but differ in focus and interpretation. At any rate, it should be noted that Fig. 6C has been eliminated from the current version of our manuscript for reasons detailed in our response to reviewer 3 (please see point no. 6 below)

We hope this clarifies the distinct contributions of the two studies.

Miscellaneous comments

The statement on line 116-118 is not supported by statistics - authors should display fold change/statistics on IgG control vs α-CXCR2 treated Csf3r-/- and WT mice. It appears that anti-cxcr2 Ab do abrogate neutrophilia in both instances.

Response. We thank the reviewer for this helpful remark. The text has been revised (lines 115 -117) to better clarify that anti-CXCR2 treatment completely prevented any increase in blood neutrophil counts in Csf3r$^{-/-}$ mice, whereas this effect was only partial in anti-CXCR2-treated WT mice. The statistical analysis of differences between IgG control vs α-

CXCR2 treated Csf3r$^{-/-}$ and WT mice is shown in Fig. 2A, as recommended by the reviewer.

For lines 131-133, the authors suggest that CLP is an excellent model of human sepsis. Given the research using CLP models and the multitude of effective interventions in the mouse, the authors should explicitly state how such research has translated into effective treatments for human sepsis. If this is not the case, perhaps it cannot be considered as a good model of human sepsis.

Response. We thank the reviewer for this insightful comment. In the revised manuscript, we now explicitly acknowledge the limitations of all mouse sepsis models (including CLP) in translating preclinical findings into effective treatments for human sepsis (please see the last sentence of the Discussion section). While CLP reproduces certain key features of sepsis, as we note with reference to an authoritative study (ref 16), we also clarify that therapeutic interventions successful in mice may not demonstrate clinical efficacy in humans within the field of sepsis research (lines 391-395).

In line 149, 'almost completely unable' is perhaps an overstatement when referencing IL-1b and IL-18 responses.

Response. We thank the reviewer for this helpful suggestion. In the revised manuscript, we have rephrased the text to more accurately describe the IL-1β and IL-18 responses (please see lines 149-151).

Information on statistical tests performed is required in figure legends for S1, S6, and S7.

We appreciate the reviewer highlighting this omission. The figure legends have now been updated to include the number of experiments and the statistical tests performed.

Reviewer #3

1. Overall assessment

This manuscript reports that IETD treatment results in cell death-independent TNF-RIPK1-RIPK3 induced cytokine transcription and their activation of neutrophil elastase/cathepsins to trigger GSDMD and IL-1β. However, key data to support these conclusions is, for the most part, lacking.

Response. We appreciate the reviewer's perspective and have added clarifications and new experiments in the revised manuscript addressing the role of elastase/cathepsins in GSDMD processing, in line with the reviewer's recommendation. The specific criticisms raised by the reviewer concern four main points, which will be discussed separately below:

- the role of elastases and cathepsins in GSDMD cleavage;
- potential off-target effects of the IETD caspase-8 inhibitor used;
- the involvement of cell death and GSDMD/RIPK3 in the observed phenomena;
- similarities with our previous publication.

2. Role of serine proteases (elastase, cathepsin G and proteinase 3) in GSDMD processing

General comment. *First, the authors never show that IETD activates neutrophil elastase/cathepsins (data is based on an inhibitor studies only).*

Response. Thanks for this comment. We respectfully disagree with the statement that our data were limited to inhibitor studies. Figure S3C (previous and present manuscript versions) shows that IETD markedly upregulated the expression of granule protein genes including neutrophil elastase and cathepsin G. However, we acknowledge that these data have not been sufficiently highlighted in the main text of the original manuscript, and we apologize for this lack of clarity. While it is logical to assume such transcriptional upregulation is associated with increased enzymatic activity (granule serine proteases are transcriptionally regulated and are constitutively active in mature neutrophils) we have, following the reviewer's suggestion, performed additional experiments to directly assess protein levels of elastase upon IETD treatment. Results support our transcriptional data and now show that neutrophil elastase is upregulated also at the protein level in a RIPK3-dependent manner (Figs. S6E and F), in compliance with the reviewer's suggestion. In addition, these results are now more clearly presented (lines 260-276)

General comment. *Second, they never show that loss of elastase/cathepsin activity reduces GSDMD and IL-1beta activation (i.e. processing) and activated (p17) IL-1b release.* **Specific comment #8.** *Fig. 7C. Contrary to the authors assertions, BAY85 and MeoCuc, do not reduce GSDMD p23 levels (i.e. this product looks similar to IETD control treated cells). Infact these inhibitors result in an increase in p30 levels - which represents the pore-forming fragment of GSDMD. One would therefore9 predict these cells to exhibit increased rates of pyroptotic death.*

Response. We thank the reviewer for this important comment. We respectfully disagree with the reviewer's interpretation of the original Western blot (WB) data (Fig. 7C) for reasons that will be clarified below. However, we acknowledge that these blots were not technically ideal and have repeated WB experiments to further clarify the effects of serine protease inhibitors on GSDMD processing (Fig. 7C, revised manuscript). We also revised the text to more clearly describe these findings (lines 260-276). We believe the new data (new Fig.7C and S6E) and the clarifications introduced in the text now convincingly show the role of neutrophil elastase, cathepsin G and perhaps other serine proteases in GSDMD processing and IL-1β release.

In the original blot, GSDMD processing—when quantified as the ratio of processed to unprocessed band intensity rather than the intensity of a single processed form)—appeared to be completely or partially prevented by the specific inhibitors used, in agreement with what stated in the text and –importantly– with the newly performed experiments. However, we acknowledge that total GSDMD levels (processed + unprocessed) was variable across lanes (Fig. 7C, original manuscript), which made this blot technically suboptimal and complicated this interpretation. This is the reason why we repeated the experiments which now more clearly demonstrate inhibition of GSDMD processing by MeOSuc-AAPV-CMK, and partial inhibition by the elastase inhibitor BAY 85-8501 and the cathepsin G inhibitor JNJ-10311795, as mentioned above.

Third, the authors never examine if RIPK1 D138N and RIPK3 KO (and MLKL KO control) neutrophils have reduced IETD-driven cathepsin/elastase activity and consequently reduced GSDMD/IL-1beta activation (i.e. processing to their bioactive fragments).

Response. Thanks for this suggestion. We have now assessed elastase levels in IETD-treated neutrophils from *RIPK3* KO mice. Our results show that IETD-induced increases in elastase activity are completely abolished in *RIPK3* KO neutrophils (Fig. S6E).

3. Off-target effects of Z-IETD-fmk (IETD)

Fourth, the authors do not acknowledge that Z-IETD-fmk exhibits significant cross reactivity towards other caspases, the fmk group has well documented off-target effects and fmk-based inhibitors can also cross-react with lysosomal proteases. For transparency, the authors should not hide these caveats. I also don't think the authors can claim the lack of effect of IETD in caspase-8/MLKL DKO mice as evidence that it is acting "on target" as caspase-8/MLKL loss profoundly impacts immune cell transcriptional programs - so these mice fail to respond normally to most inflammatory stimuli, including IL-1 transcription.

Response. We agree that Z-IETD-fmk can exhibit off-target effects, particularly at high concentrations or with prolonged exposure. We did not intend to obscure this caveat. In fact, it has been extensively addressed in our previous study (Cell Reports Medicine, https://doi.org/10.1016/j.xcrm.2023.101098), using both genetic and pharmacological approaches, and it is re-examined in the present manuscript. Our results demonstrate that, under the conditions used, the effects of Z-IETD-fmk are attributable solely to caspase-8 inhibition. This conclusion is directly supported in the present study by observations that IETD-induced neutrophilia (Fig. S1A), cytokine induction (Figs. S1B and C) and gene regulation (Fig. S3G) are absent in Casp8-MLKL double knockout mice but preserved in MLKL single knockouts.
We appreciate the reviewer's concern that *Casp8–Mlkl* double knockout mice might display broad defects in inflammatory signaling. However, previous work from our lab and from that of others has shown that immune cells from these mice produce normal or even elevated levels of proinflammatory cytokines in response to LPS, poly(I:C), and PMA plus ionomycin stimulation (*Immunity*, 2016, 45:513–526; Figs. 7C–D, S7–S8). We have now revised the manuscript to clarify that *Casp8–Mlkl* double knockout mice completely retain their ability to respond to inflammatory stimuli and cite the above- mentioned reference (lines 124-127).

4. GSDMD cleavage and RIPK3 phosphorylation as cell death markers

General comment of Referee 3. *Fifth, as detailed below, I do not believe the authors can claim their observations occur independent of cell death as the neutrophils ... exhibit markers of both necroptosis (P-RIPK3) and pyroptosis (GSDMD cleavage).*

Specific comment 13 of Referee 3. *The authors conclude (line 306) that the effects they observe occur "without evidence of necroptosis, pyroptosis or other forms of cell death". 1st the authors observe P-RIPK3 upon IETD treatment, which is a marker o necroptosis. 2nd they observe GSDMD activation/processing, a marker of pyroptosis.*

Response. While both GSDMD cleavage and RIPK3 phosphorylation have been traditionally associated with pyroptosis and necroptosis, respectively, it has been recently shown that these events do not necessarily result in cell death, particularly in immune cells. First, GSDMD activation is not an infallible marker of pyroptosis. In a highly cited study, Jonathan Kagan's group (PMID: 29195811) demonstrated that activated GSDMD is required for IL-1β secretion in living macrophages that do not undergo pyroptosis. It is now

widely accepted that GSDMD activation is essential for IL-1β release in various types of living, activated cells (please see ref, 22 and 23 of the revised manuscript). This point is now clarified with reference to this seminal publication (lines 274-276)

Second, RIPK3 phosphorylation should not be regarded as an infallible indicator of necroptosis. This point was in fact addressed in our original manuscript, though it appears to have been overlooked by the reviewer. For clarity, we reproduce a relevant excerpt from the Discussion section, which has been kept in the revised manuscript (lines 359-362) along with the supporting references: *"RIPK3 activation can drive cytokine production independently of MLKL and necroptosis. For example, RIPK3 was necessary for cytokine responses in macrophages activated with LPS in the presence of a pan-caspase inhibitor (24), in LPS-stimulated dendritic cells (38), and in virally infected brain cells (39)".*

5. Independence of IETD-induced changes from spontaneous neutrophil death

General comment of Referee 3. *Fifth, as detailed below, I do not believe the authors can claim their observations occur independent of cell death as the neutrophils … die with and without IETD treatment*
Specific comment 12 of Referee 3. *The authors conclude that GSDMD is not causing death following IETD treatment. However, neutrophils are clearly dying (Fig. S7B) and adding IETD may have simply converted neutrophil killing from one type of cell death (eg. apoptosis) into another type of cell death (e.g. pyroptosis), so to conclude that GSDMD does not cause pyroptosis under these conditions is premature.*

Response. We respectfully but strongly disagree. The changes induced by IETD that we describe cannot be attributed to cell death (whether apoptotic, pyroptotic, or otherwise) because they occur with clear temporal separation from any detectable cell death. As outlined in our response to Reviewer 2, IETD-induced transcriptional reprogramming occurs rapidly, within 1–3 hours of treatment (Fig. 3, present manuscript). At these early time points, there is no evidence of cell death, as confirmed by dye exclusion assays (Fig. S4A, 2023 paper; Fig. S7B,C, *EMBO*). These data show that neutrophils begin to die spontaneously in culture only after several hours of incubation, in agreement with published evidence (PMID: 20133633).

Moreover, IETD treatment does not alter either the kinetics or the extent of spontaneous death. In both IETD-treated and untreated neutrophils, cell death progresses gradually (Fig. S4A, 2023 *Cell Reports Medicine* paper; Fig. S7B, present manuscript). The reviewer raises the possibility that IETD might convert apoptotic death into GSDMD-induced pyroptosis, citing the observed GSDMD activation. However, our genetic evidence directly refutes this: spontaneous death occurs at the same rate and with identical kinetics in wild-type and *Gsdmd*$^{-/-}$ neutrophils, irrespective of IETD treatment (Fig. S7B). As indicated above this point is further clarified in the revised manuscript

Together, these findings provide compelling evidence that the proinflammatory responses elicited by caspase-8 inhibition are not secondary to pyroptosis or any other form of cell death, but rather reflect a distinct, transcriptionally regulated reprogramming event.

Similarity to the 2023 Cell Reports Medicine paper

Finally, the novelty of the manuscript is diminished due to their previous study in Cell Reports Medicine (https://doi.org/10.1016/j.xcrm.2023.101098) where they showed that IETD activated

RIPK1 and RIPK3 to drive gene transcription of cytokines such as IL-1 and also enabled increased anti-pathogen defense.

Response. We respectfully disagree with the reviewer's assessment. As detailed in our response to Reviewer #2, the present study provides several novel findings that go well beyond RIPK1/3-dependent cytokine induction and our previous *Cell Reports Medicine* publication. For example, in the present study for we identify for the first time the presence of autocrinous, tonic TNF-α/TNFR1 signal in neutrophils; we document its essential role in driving caspase-8- controlled reprogramming of more than one fourth of the neutrophil transcriptome. Careful analysis of regulated genes revealed that they involve almost any aspect of neutrophil biology while only a only a minority concerns cytokine production. Finally, whereas our previous work focused on local inflammatory responses, the current manuscript investigates neutrophilia secondary to neutrophil release from hematopoietic tissues. Unfortunately, these novel findings were apparently missed by the reviewer, who only focused on RIPK1 and RIPK3-induced transcription of cytokine gene and GSDMD processing, which constitute only a fraction of the data presented.

Role of caspase 1/11 in IL-1β release

General comment*. This previous study also documented that caspase-1/11 DKO mice exhibited significantly reduced IL-1b and also abrogated neutrophilia upon IETD injection - which contradicts key conclusions of the current paper.*
Specific comment 10*. .... This is important as the authors previous Cell Reports Medicine paper showed theat the caspase-1/11 DKO mice injected with IETD showed the same effects to the GSDMD KO mice used in this paper (i.e. reduced IL-1beta levels and also neutrophil levels that were almost back to untreated levels).*

Response: We respectfully disagree with the assertion that the current manuscript contradicts our previous findings regarding the role of caspase-1/11 in IL-1β release. The two studies investigate different biological processes and utilize distinct experimental readouts, making them not directly comparable. In our previous *Cell Reports Medicine* paper, analysis of peritoneal lavage fluid from IETD-injected mice showed moderate, statistically significant reductions in IL-1β levels (~37%) and neutrophil recruitment (~57%) in the peritoneal cavity of *Casp1/11* DKO mice. Blood neutrophil counts were not addressed in that study. Thus, the claim that in our published study caspase-1/11 deficiency "abrogated neutrophilia upon IETD injection" is inaccurate for two reasons: (1) "neutrophilia" refers to elevated circulating neutrophil counts, which were not assessed in that study, and (2) the reductions were partial, not complete, and should not be described as "abrogation."

The statement that "*Casp1/11* DKO mice showed the same effects as the *Gsdmd* KO mice used in the current study" is also inaccurate. In this study, *Gsdmd* deficiency led to nearly complete suppression (~80% reduction) of circulating IL-1β, whereas circulating cytokine levels were not measured in *Casp1/11* DKO mice in either the previous or current studies. The datasets, therefore, address different questions and are not contradictory. Our present data show that caspase-1/11 deficiency only marginally impairs IL-1β processing, and we find no evidence of caspase-1 activation following IETD treatment. As indicated in our previous published paper (right-hand column, page 4 and Fig. S6A), the reduced IL-1β levels that were observed in *Casp1/11* DKO mice may have reflected markedly decreased pro-IL-1β gene transcription, rather than impaired pro-IL-1β processing. Consistent with this, we recently observed that these mice also exhibit reduced production of other

proinflammatory cytokines, including TNF-α and IFN-β, in response to LPS stimulation, as also reported by others using single Casp1 ko mice (PMID: 32101731). Based on these findings, we have concluded that the *Casp1/11* DKO strain used in our previous study and indeed perhaps other mouse strain lacking the scaffold function of caspase-1 may not be optimal for investigations on procaspase 1 processing, such as the present one. Moreover, the *Casp1/11* DKO strain derived from the original *Casp1* knockout on a 129 background, carries a natural *Casp11* null allele, and its mixed 129/C57BL/6 genetic background may contribute to strain-dependent immune variation despite extensive backcrossing. We plan to address the role of caspase-1 in IETD-induced GSDMD and pro–IL-1β processing using newly developed mice expressing enzymatically inactive caspase-1 but retaining intact transcriptional activation of pro-IL1β and other inflammatory genes (PMID: 32101731). This mouse line is currently cryopreserved and not yet available for experimentation; therefore, these studies will require several months and will be presented in a separate manuscript.

For these reasons, we have removed the *Casp1/11* DKO data, as well as the caspase-1 western blots, from the current manuscript. The role of caspase-1 is not central to this study, and their removal does not affect our main conclusion: that GSDMD is required for IETD-induced increases in circulating IL-1β and CXCL1, whereas GSDME, MLKL, and canonical inflammasome components such as ASC and NLRP3 are not involved.

Point-by-Point Response to other specific comments of Reviewer #3

(Reviewers' comments in italic, our responses in regular font*)*

Specific comment #1

The number of times each experiment, including the in vivo experiments, were repeated and results reproduced needs to be clearly stated in the figure legends. As currently written, it is unclear how reproducible the data was across independent experiments.

Response. We thank the reviewer for this helpful comment. We have now clarified the number of experiments performed where this information was previously unclear (figure legends for Figs. 1F, 2C–E, S1, S5, and S7).

Specific comment #2

The authors find that z-IETD-fmk increased responses in MLKL KO mice (Fig. S1), which is an important observation. For example, is MLKL loss prolonging the survival of caspase-8 inhibited cells, thereby enabling increased cytokine production and subsequent neutrophil counts?

Response. We thank the reviewer for this insightful comment. However, to answer the reviewer question, the loss of MLKL does not prolong or otherwise affect neutrophil survival, either in the presence or absence of caspase-8 inhibition, as shown in Fig. S4A of our *Cell Reports Medicine* paper (10.1016/j.xcrm.2023.101098).

We agree that the increased cytokine release observed in this and our previous studies using animals with permanent loss of pore-forming proteins such as MLKL or GSDME is an interesting phenomenon. Our working hypothesis is that this may reflect

adaptive/compensatory changes associated with chronic deficiencies in single factors (such as MLKL or GSDME), which are known to promote cytokine release under specific circumstances. However, elucidating the mechanisms underlying the increased cytokine release in these settings is beyond the scope of the present study. Here, we used MLKL KO and GSDME KO mice to determine whether MLKL or GSDME are required for responses to caspase-8 inhibition. The results clearly show that they are not, thereby ruling out the involvement of necroptosis and GSDME-dependent pyroptosis, respectively.

Specific comment #3

Related to point 2 - caspase-8 inhibition would be expected to trigger necroptosis in susceptible cell types - do the authors observe any evidence of this other than P-RIPK3 detection and the indirect data suggesting this is the case (presented in Fig. S1)?

Response. Please see above our response to point 2. Briefly: 1) as discussed in the manuscript and in our replies to the reviewer's general comments and to specific comment 2, there is no evidence that the caspase-8 inhibitor IETD triggers necroptosis either *in vivo* or *in vitro* under the conditions used in this study; 2) as noted in the manuscript, RIPK3 phosphorylation is not necessarily indicative of necroptosis. That activation of RIPK3 can promote cytokine induction independently of cell death is well established in the literature (see refs. 20, 34, and 35). 3) The data presented in Fig. S1A formally exclude necroptosis as the cause of IETD-induced inflammatory changes, since responses to IETD also occur in the absence of MLKL, the essential executioner of necroptosis. Moreover, we do not consider the findings in Fig. S1A, or any other results from our study, to represent indirect evidence that IETD-induced caspase-8 inhibition triggers necroptosis.

Specific comment #4

The cecal ligation punction data (Fig. 2) should be repeated in casp8/MLKL DKO and MLKL KO mice to define if the impact observed is a result of MLKL being triggered by caspase-8 inhibition, or otherwise.

Response. We thank the reviewer for this valuable comment. We note that the experiments suggested by this reviewer would require substantial time and resources, including obtaining new ethical approvals from local and national authorities, as well as breeding and genotyping a conspicuous number of single and double KO animals, since these colonies must be maintained at low numbers. We would also like to respectfully point out that, in our view, such experiments would not offer a favourable benefit-to-cost ratio regarding the use of laboratory animals.

Across our previous and present studies, we have examined a wide range of IETD-induced effects in *Casp8/MLKL* double knockout and *MLKL* single knockout mice, including neutrophil recruitment to sites of inflammation, neutrophilia, elevations in circulating and tissue cytokine levels, and regulation of several cytokine and non-cytokine genes. In all cases, without exception, MLKL was dispensable for the responses to IETD both *in vivo* and *in vitro*. We therefore see no reason to expect that IETD-induced potentiation of host defenses in the cecal ligation and puncture model would represent an exception. For these reasons, we prefer not to include additional experiments with CLP in MLKL-deficient mice to the present study.

Specific comment #5

GSK872 can induce apoptosis via causing a conformational change in RIPK3, so the authors statement, line 222, that "the kinase function of RIPK3, rather than its scaffold functions, is required for neutrophil responses to IETD" may not be the case.

Response. We thank the reviewer for this insightful comment. We now address this point in the revised manuscript (lines 227–233) and cite a relevant study (Mandal, P. et al., *RIP3 induces apoptosis independent of pronecrotic kinase activity*, Mol. Cell 56, 481–495, 2014). We agree that, in principle, GSK-872–induced, caspase-8–dependent apoptosis could have influenced the responses to IETD. To exclude this possibility, we performed additional experiments using short incubation times with GSK-872, within a time frame during which apoptosis—which typically requires several hours to develop—is unlikely to occur. These new data are presented in Figs. S5C and D of the revised manuscript. Moreover, we found that GSK-872 did not increase cell death under either short or prolonged incubation conditions (new Fig. S5E). The absence of apoptosis under these conditions may be related to co-incubation with a caspase-8 inhibitor, as required by our experimental design.

Specific comment #6

See our response above to the general comments of this reviewer under the heading "Role of caspase 1/11 in IL-1β release"

Specific comment #7

Figure 6F and Fig. S1A - the data do not agree with other. In one data set neutrophil counts are significantly increased in MLKL KO mice, but in the other data set there is no difference. This speaks to the importance of defining in the figure legends how many times the experiments were reproduced.

Response. We thank the reviewer for this helpful comment. The number of animals used in the experiments shown in Fig. S1A has now been added to the figure legend. We apologize for this omission in the original manuscript.

We respectfully disagree with the statement that the data in Fig. 6F and Fig. S1A contradict each other. Both figures clearly show that treatment with IETD induces marked neutrophilia in MLKL KO mice. The purpose of these experiments was to determine whether MLKL (and also caspase-8 in Fig. S1A) is required for IETD-induced neutrophilia by comparing the responses of wild-type (WT) and MLKL KO animals. Results in either figure demonstrate that MLKL is not required for this response, since IETD caused robust neutrophilia in both WT and MLKL KO mice. Accordingly, as stated in the text, we conclude that MLKL is dispensable for the in vivo response to IETD administration. No other interpretation or conclusion is drawn in the text from the data shown in Fig. 6D or Fig. S1A.

The experiments were not designed to determine whether the *magnitude* of neutrophilia differs between MLKL KO and WT animals. Nevertheless, both datasets show a similar trend—neutrophil counts tend to be higher in MLKL KO mice compared to WT mice. The only difference is that this trend reaches statistical significance in one dataset (Fig. S1A) but not in the other (Fig. 6D). Such minor variations are common in biological experiments and are not relevant to the main conclusion of our study, which is that MLKL is not required for IETD-induced neutrophilia or for elevations in circulating cytokines.

Specific comment #8

Fig. 7C. Contrary to the authors assertions, BAY85 and MeoCuc, do not reduce GSDMD p23 levels (i.e. this product looks similar to IETD control treated cells). Infact these inhibitors result in an increase in p30 levels - which represents the pore-forming fragment of GSDMD. One would therefore predict these cells to exhibit increased rates of pyroptotic death.

See our response to this comment above under the heading "Role of serine proteases (elastase, cathepsin G and proteinase 3)"

Specific comment #9

Fig. 7D. The differences reported are very small - ~ 80 pg/ml in control vs 50-60 pg/ml in inhibitor treatments. These data also do not distinguish between inhibitor impacts on IL-1beta gene transcription vs IL-1beta processing and activation, which is critical for its interpretation and the authors conclusions. As the neutrophils spontaneously die over time (Fig. S7B) they could simply be releasing precursor (inactive) IL-1beta. The examination of GSDMD/IL-1beta activation (i.e. processing in cell lysates and supernatants) is required in these treatment conditions.

Response. We thank the reviewer for this insightful comment. However, we respectfully disagree with the conclusion that examining GSDMD and IL-1β processing is required to interpret the data shown in Fig. 7D. The inhibitors used in these experiments specifically target signalling molecules such as MAPKs, other kinases, and NF-κB, which are well established to regulate transcription of the *Il1b* gene and other cytokine genes. These pathways are not known to directly influence the processing of pro–IL-1β or GSDMD.

Therefore, under these experimental conditions, the observed effects of the inhibitors on IL-1β levels in the supernatants reflect modulation of *Il1b* gene activation rather than post-translational processing, regardless of neutrophil viability. Consistent with this interpretation, similar inhibitory effects were observed on Cxcl1 production—a chemokine whose expression is regulated exclusively at the transcriptional level.

Specific comment #10

Given the authors conclude that IETD treatment results in RIPK1-RIPK3 activation to allow triggering of GSDMD and IL-1beta release via serine proteases, they need to examine GSDMD processing/activation, IL-1beta activation (i.e. p17 levels in neutrophil cell lysates and supernatants) and elastase/cathepsin activity using TNFR1 KO, RIPK1 kinase dead, RIPK3 KO, and control - MLKL KO, caspase-1/11 DKO - neutrophils. This is important as the authors previous Cell Reports Medicine paper showed theat the caspase-1/11 DKO mice injected with IETD showed the same effects to the GSDMD KO mice used in this paper (i.e. reduced IL-1beta levels and also neutrophil levels that were almost back to untreated levels).

Response. Thanks for this comment. Following the reviewer suggestion, we have examined neutrophil elastase levels in wild type and RIPK3 KO animals in the revised manuscript . Regarding the supposed similarity between the caspase 1/11 ko data reported in our previous manuscript and the GSDMD data reported in the present one, please see our response above under the heading "Role of caspase 1/11 in IL-1β release"

Specific comment #11

Fig. S7B. WT and GSDMD KO death kinetics - mean and error - looks very similar between the WT and GSDMD KO mice. For example, one would be hard pressed to find differences if these graphs were overlayed, particularly for the vehicle WT and vehicle GSDMD KO data.

Response. We thank the reviewer for the careful and detailed examination of these data. We agree that the death kinetics—mean and error—appear similar between WT and GSDMD KO neutrophils. This observation supports the conclusion that GSDMD does not contribute to either spontaneous or IETD-induced cell death, which is an important finding of the study. Regarding this point see also our response below to specific comment #12. While the curves are indeed close, they are not identical, as can be verified from the raw data provided in the supplementary material.

Specific comment #12

The authors conclude that GSDMD is not causing death following IETD treatment. However, neutrophils are clearly dying (Fig. S7B) and adding IETD may have simply converted neutrophil killing from one type of cell death (eg. apoptosis) into another type of cell death (e.g. pyroptosis), so to conclude that GSDMD does not cause pyroptosis under these conditions is premature.

Response. Please see our response above to this and similar comments under:
5. Independence of IETD-induced changes from spontaneous neutrophil death.
Briefly, Fig. S7B provides compelling evidence that IETD treatment does not cause GSDMD dependent pyroptosis since the number of dead neutrophils is similar in wild type and GSDMD-deficient neutrophils after IETD treatment, as noted by the referee under specific comment 11.

Specific comment #13

The authors conclude (line 306) that the effects they observe occur "without evidence of necroptosis, pyroptosis or other forms of cell death". 1st the authors observe P-RIPK3 upon IETD treatment, which is a marker o necroptosis. 2nd they observe GSDMD activation/processing, a marker of pyroptosis. 3rd the neutrophils are dieing over time with and without IETD. To claim all IETD-induced neutrophila and elevated cytokines is therefore cell death independent is not warranted.

Response. Please see our response above to this and similar comments under 4. **GSDMD cleavage and RIPK3 phosphorylation as markers of, respectively, pyroptosis and necroptosis** and **5. Independence of IETD-induced changes from spontaneous neutrophil death.**

Specific comment #14

A model schematic would be informative.

Response. Thank you for the helpful suggestion. We have now included a model schematic in the revised manuscript (Fig. S8).

Dear Prof. Teti,

Thank you for the submission of your revised manuscript to our editorial offices. I have now received the reports from the two referees that I asked to re-evaluate the study, you will find below.

As you will see, the referees have further comments and suggestions to improve the manuscript. During cross-commenting, referees nevertheless indicated that a further revision should be invited. I thus ask you to address the referee comments in a further revised manuscript. Please also provide a detailed further p-b-p-response regarding the remaining referee points and the editorial requests below.

Editorial requests:

- The Expanded View format, which will be displayed in the main HTML of the paper in a collapsible format, has replaced the Supplementary information. Thus, please combine the figures presently shown as Supplementary Figures (S1-S8) in the Appendix to have not more than 6 EV figures and upload these as separate EV figures. Please follow the nomenclature Figure EV1, Figure EV2 etc. and put the figure legend for these in the main manuscript document file in a section called Expanded View Figure Legends after the main Figure Legends section (see below). Please update all callouts and finally remove the file with the Supplementary Figures (Appendix). Finally, please also remove the part 'Extended Data' from the manuscript text file.

- Please reduce the number of keywords to five and order the manuscript sections like this, using only these names:
Title page - Abstract - Keywords - Introduction - Results - Discussion - Methods - Data availability section - Acknowledgements - Disclosure and Competing Interests Statement - References - Figure legends - Expanded View Figure legends

- We now use CRediT to specify the contributions of each author in the journal submission system. CRediT replaces the author contribution section. Please use the free text box to provide more detailed descriptions and do NOT provide your final manuscript text file with an author contributions section. See also our guide to authors (section 'Author contributions'):
https://link.springer.com/journal/44319/submission-guidelines#cms-Revised-submissions

- Please check again that the number "n" for how many independent experiments were performed, their nature (biological versus technical replicates), the bars and error bars (e.g. SEM, SD) and the test used to calculate p-values is indicated in the respective figure legends (main and EV figures). Please also check that all the p-values are explained in the legend, and that these fit to those shown in the figure. Please provide statistical testing where applicable. Please avoid the phrase 'independent experiment' but clearly state if these were biological or technical replicates. Please also indicate (e.g. with n.s.) if testing was performed, but the differences are not significant. In case n=2, please show the data as separate datapoints without error bars and statistics. See also:

https://link.springer.com/journal/44319/submission-guidelines#cms-Figure-and-data-presentation

If n<5, please show single datapoints for diagrams. Moreover:
- Please note that the figure 1F is mislabeled as figure 1E in the manuscript. This needs to be rectified.
- Please note that the exact p values are not provided in the legends of figures 1A, C, F; 2B-E; 4A-E; 5D, E; 6A-E; 7A, B, C, F, G.
- Please note that information related to n is missing in the legends of figures 1E, 2C-E; 4A, 7B
- Please note that the error bars are not defined in the legends of figures 1C, E

- The data availability section (DAS) is restricted to information about large datasets generated in the study that have been deposited externally. Please provide here only access information for deposited dataset and direct links. Please remove now all referee tokens and make sure that the datasets are public latest upon online publication of the manuscript.

- Please use our reference format (et al needs to be used after 10 author names; DOIs should only be used for preprints and datasets that have not been published yet):
https://link.springer.com/journal/44319/submission-guidelines#cms-Reference-guidelines

- Per journal policy, we do not allow 'not shown', which is stated in the manuscript (page 7). All data referred to in the paper should be displayed in the main or Expanded View figures, or an Appendix. Thus, please add these data (or change the text accordingly if these data are not central to the study). See:
https://link.springer.com/partners/embo-press/editorial-policies#Data%20deposition

- Please remove the 'Materials Table' from the manuscript main text file. Please upload this as separate file. The downloadable templates (.doc) for the Reagents and Tools Table can be found in our author guidelines (section 'Structured Methods'):

https://link.springer.com/journal/44319/submission-guidelines#cms-Manuscript-organisation-and-formatting

Please also add callouts to the R&T table where appropriate.

- Please make sure that all the funding information is also entered into the online submission system and that it is complete and similar to the one in the acknowledgement section of the manuscript text file. Moreover, the info provided in the Comments box in the submission system will not be transferred to the publisher. So, this needs to be added in the system via the 'More Funders' option. Please do that.

- The resolution of the submitted figures is too low for the blot images. This reduction in resolution is commonly caused by converting original 16-bit TIFF files to RGB format for publication. While this is not inherently problematic, it can raise concerns about image integrity for critical readers.

To avoid any misunderstanding and to meet EMBO Press standards, we kindly ask that you:

- to resubmit the complete figure set at the captured original data resolution.

- to apply the same resolution standards to the blot source data files, which are also currently below the required quality threshold. Please upload the blot source data files as .tiff or pdf. Please do not use PowerPoint.

- In many source data files for numerical data (excel files), e.g. for Fig. 4, values for vehicle controls are always 7,8. This seem odd. Please check and verify.

In addition, I would need from you uploaded separately:
- a short, two-sentence summary of the manuscript (not more than 35 words).
- two to four short (!) bullet points highlighting the key findings of your study (two lines each).
- a schematic summary figure as separate file that provides a sketch of the major findings (not a data image) in jpeg or tiff format (with the exact width of 550 pixels and a height of not more than 400 pixels) that can be used as a visual synopsis on our website.

As part of the EMBO publication's Transparent Editorial Process, EMBO reports publishes online a Review Process File (RPF) to accompany accepted manuscripts. This File will be published in conjunction with your paper and will include the referee reports, your point-by-point response and all pertinent correspondence relating to the manuscript.

You are able to opt out of this by letting the editorial office know (emboreports@embo.org). If you do opt out, the Review Process File link will point to the following statement: "No Review Process File is available with this article, as the authors have chosen not to make the review process public in this case."

I look forward to seeing the further revised version of your manuscript when it is ready. Please let me know if you have questions regarding the revision.

Yours sincerely,

Achim Breiling
Senior Editor
EMBO Reports

Referee #2:

Kinetic cell death assays have not been performed for key genotypes in question for this study (eg. for GSDMD ko, GSDME ko, Casp8/RIPK3 DKO, Casp8/MLKL DKO when treated with z-IETD). The authors state that this reviewer "does not provide specific evidence to support a role" for Caspase-8 in preventing necroptotic cell death, however the published data in this space is immense, particularly in the past 15 years. While I do not have any concerns that z-IETD might be promoting some gene expression in the presence of basal TNF, there is also little experimental evidence that this is also not driving the death of neutrophils in the hours following cytokine production, introducing significant possible caveats to these data. Caspase-8 deficiency in neutrophils leads to cell death, so it is reasonable to rigorously assess this possibility. The cell death assay in Fig S5 shows ~50% loss of viability after 8h, indicating that a very strong mitochondrial apoptotic trigger is (expectedly) underway in these cultures - particularly when these cultures do not contain ANY neutrophil survival cytokines like G-CSF, GM-CSF, or IFNg to prevent the induction of mitochondrial apoptosis. It is unclear how a different cell death pathway can be studied effectively when mitochondrial apoptosis is active in the population of neutrophils.

Why in Fig S6 is neutrophil elastase not also measured in MLKL KO, rather than only RIPK3 ko? How many times has this

experiment been repeated? Individual data points should be shown.

The reference to the Alvarez-Diaz paper in Immunity, Fig 7D, suggesting that Casp8/MLKL DKO "produce normal or even elevated levels of proinflammatory cytokines in response to LPS, poly(I:C)", is an error in interpretation. While T cells can produce increased RANTES and IL-10 in response to CD3+CD28, these mice also develop severe autoimmune disease - very likely the T cells are abnormal in these mice. In the same figure, it is very clear that BMDM are hypo-responsive to polyIC and LPS.... Rev.3 notes this very important point.

Referee #3:

I'd like to thank the authors for the detailed and thoughtful answers to my queries. I believe this manuscript is suitable for publication in EMBO reports, should the authors address my remaining concerns/comments:

1 .Regarding neutrophil elastase and cathepsin activity - I was suggesting that it would be important assess their activity through fluorogenic or chromogenic peptide substrate assays (e.g., Abcam, ab126780), as this would directly determine if there was increased activity up IETD treatment, and if this is reduced in RIPK3/MLKL KOs, rather than current inferences that are made only from expression data. I don't think the statement that "Our results show that IETD-induced increases in elastase activity are completely abolished in RIPK3 KO neutrophils (Fig. S6E)." is accurate, as this data represents an ELISA that measures elastase levels, not its activity.

2. The original Figure 7C and revised manuscript Figure 7C show completely different results. The original Figure 7C showed that IETD resulted in a loss of full length GSDMD and increased GSDMD p23 (with GSDMD p30 being almost undetectable), while the addition of MeOSuc, BAY85 and JNJ-103 all resulted in GSDMD p30 being retained (MeOSuc) or increased (BAY85 and JNJ-103) compared to controls, with BAY85 and JNJ-103 also increasing levels of full-length GSDMD. In contrast, the new Fig. 7C shows robust GDMD p30 generation upon IETD treatment (not disappearance), with MeOSuc, BAY85 and JNJ-103 all reducing levels of cleaved p30 GSDMD (not increasing). These contradictory results between the original and revised manuscript make it impossible to know what is going on. How many times has the experiment been reproduced and what were the results from each independent experiment?

3. The authors write "However, previous work from our lab and from that of others has shown that immune cells from these mice produce normal or even elevated levels of proinflammatory cytokines in response to LPS, poly(I:C), and PMA plus ionomycin stimulation (Immunity, 2016, 45:513-526; Figs. 7C-D, S7-S8)." This statement is factually incorrect. First, there is no Fig. S8 in this paper. Second, Fig. S7 shows that caspase-8 and MLKL deletion reduces LPS and polyIC driven IL-1alpha, IL-1beta, IL-3, IL-6, IL-12, IL-13, IL-17, eotaxin, G-CSF, IFN-gamma, KC, to name a few. Infact, based on this paper one would argue that caspase-8 and MLKL loss reduces many, if not most, inflammatory factors, with CD3 plus CD8 antibody stimulated T-cell increased cytokines (eg RANTEs, IL-10) being the exception. To further write that "...Casp8-Mlkl double knockout mice completely retain their ability to respond to inflammatory stimuli and cite the above- mentioned reference (lines 124-127)." is misleading. While these mice develop autoimmune proliferative syndrome with age, this results from defective apoptosis and increased T-cell numbers, and a sub-set of increased T-cell cytokines. In fact, other studies have shown that caspase-8 and MLKL/RIPK3 loss in mice renders these animals incredibly resistant to damaging inflammation, because of the loss of caspase-8-driven cytokine responses (e.g., PMID: 41233351, PMID: 40632844).

4. While the authors cite studies suggesting activated GSDMD and RIPK3 do not always result in death, I am questioning whether the authors own data suffice to draw the conclusion that their observations occur "without evidence of necroptosis, pyroptosis or other forms of cell death". As I noted in my original comments, the authors show markers of pyroptosis and necroptosis in neutrophils upon IETD treatment and the neutrophils are also still dying over time with and without IETD treatment. Fig 3. shows transcriptional changes at 3 hours. The corresponding death data in Fig. S7B/C show cell viability has reduced from ~90% to under 80% in IETD treated cells at 3 hrs. While I agree that the transcriptional changes are unlikely to reflect the impact of dying cells (although GSDMD-driven IL-1beta release may), I don't think the authors can state that IETD-mediated activation of GSDMD or RIPK3 is not causing death in this situation and to do so would require more detailed experimentation. As an example as to why this level of detail is required, I note the meticulous single cell analysis recently performed on human monocytes to show that death is a driver of IL-1 secretion in human monocytes, disproving previous high profile population-level evidence concluding that IL-1 secretion was death-independent (PMID: 41219490). Consequently, my recommendation would be that the authors tone down their conclusions on activated RIPK3 and GSDMD driving cytokine responses without cell death occurring downstream of RIPK3/GSDMD, and make it clear to readers that death may or may not occur via their activity and that further experimentation is required to resolve these two possibilities.

**Manuscript #**	EMBOR-2025-62947V3
**Title**	Caspase-8 silences cell death-independent constitutive immune activation driven by tonic TNF-α
**Corresponding Author**	Giuseppe Teti

To the Editors of EMBO Reports

Point-by-Point Response to the remaining reviewers' comments

For ease of consultation, we have copied here the Reviewers' comments (*italic*) before our response (regular font). For clarity, comments and responses are broken down into sections

Reviewer #2

Comment. *Kinetic cell death assays have not been performed for key genotypes in question for this study (eg. for GSDMD ko, GSDME ko, Casp8/RIPK3 DKO, Casp8/MLKL DKO when treated with z-IETD). The authors state that this reviewer "does not provide specific evidence to support a role" for Caspase-8 in preventing necroptotic cell death, however the published data in this space is immense, particularly in the past 15 years. While I do not have any concerns that z-IETD might be promoting some gene expression in the presence of basal TNF, there is also little experimental evidence that this is also not driving the death of neutrophils in the hours following cytokine production, introducing significant possible caveats to these data. Caspase-8 deficiency in neutrophils leads to cell death, so it is reasonable to rigorously assess this possibility. The cell death assay in Fig S5 shows ~50% loss of viability after 8h, indicating that a very strong mitochondrial apoptotic trigger is (expectedly) underway in these cultures - particularly when these cultures do not contain ANY neutrophil survival cytokines like G-CSF, GM-CSF, or IFNg to prevent the induction of mitochondrial apoptosis. It is unclear how a different cell death pathway can be studied effectively when mitochondrial apoptosis is active in the population of neutrophils.*

Thank you for your comments and for the opportunity to further clarify certain aspects of our work, which once again concern the role of cell death in the phenomena we observe. As discussed extensively in our previous response, the induction of pro-inflammatory cytokines by IETD-mediated caspase-8 inhibition cannot be attributed to necroptosis, since the former process does not require MLKL, the essential executioner of necroptosis. Necroptosis, as well as other forms of cell death, can also be excluded as causes of the IETD effects because cytokine induction occurs much earlier than spontaneous cell death. Finally, IETD treatment does not increase or otherwise affect the rate of spontaneous cell death. Thus, the potential contribution of cell death to the observed phenomena has been rigorously assessed, as requested by the reviewer, and this point is discussed extensively in the revised manuscript (lines 88–92, 279–285, 319–328, 375–378).

Nevertheless, in response to this reviewer and Reviewer 3, we have now toned down our conclusions regarding the death-independent nature of the observed changes. Please see the end of the Discussion section (lines 409-412).

The reviewer states that "*kinetic cell death assays have not been performed for key genotypes relevant to this study (e.g., GSDMD KO, GSDME KO, Casp8/RIPK3 DKO,*

Casp8/MLKL DKO when treated with z-IETD)." The reviewer may have overlooked Fig. S7B and lines 279–285 of the revised manuscript. Figure S7B (now EV5G) shows detailed kinetic cell death assays performed on GSDMD KO neutrophils, while lines 279–285 describe and discuss the data presented in this figure. Briefly, IETD does not increase the rate of spontaneous neutrophil death, in agreement with previous data (ref. Lentini *et al.*, 2023), and the absence of GSDMD does not prolong viability either in the presence or absence of IETD, thereby excluding a role for GSDMD-induced pyroptosis in the observed phenomena. We did not perform kinetic cell death experiments on the other genotypes mentioned by the reviewer because, as noted above, cell death had already been excluded as a cause of the IETD-induced effects, and the primary aim of our study was not to analyze the role of signaling molecules in spontaneous cell death. Moreover, adherence to the 3R reduction principle, as stipulated in the permissions granted by local and national authorities, as well as practical considerations, limits the number of genetically modified animals that we can use.

The reviewer also notes that we stated that he/she did not provide sufficient evidence to support a role for caspase-8 in preventing necroptosis. We did not intend to make such a statement (indeed, there is no need to provide specific evidence for such a well-established role), and we sincerely apologize for our lack of clarity on this point. Our intention was simply to state that there is no evidence supporting the idea that the effects of caspase-8 inhibition observed in our study are due to necroptosis. We did not mean to criticize the reviewer's comments, and we are in fact very grateful for the suggestions provided, which helped us improve the manuscript.

Comment: *Why in Fig S6 is neutrophil elastase not also measured in MLKL KO, rather than only RIPK3 ko? How many times has this experiment been repeated? Individual data points should be shown.*

Response: The old Fig S6 (now EV5E) shows neutrophil elastase levels in RIPK3 KO mice but not in MLKL KO mice because RIPK3, but not MLKL, is required for the responses to IETD. Moreover, as mentioned above, we must minimize the number of genetically modified animals used in the experiments. The number of biological replicates is reported in the figure legends, and the individual values are provided in the raw data included in the source data files.

Comment *The reference to the Alvarez-Diaz paper in Immunity, Fig 7D, suggesting that Casp8/MLKL DKO "produce normal or even elevated levels of proinflammatory cytokines in response to LPS, poly(I:C)", is an error in interpretation. While T cells can produce increased RANTES and IL-10 in response to CD3+CD28, these mice also develop severe autoimmune disease - very likely the T cells are abnormal in these mice. In the same figure, it is very clear that BMDM are hypo-responsive to polyIC and LPS.... Rev.3 notes this very important point.*

Response. We thank this reviewer and Reviewer 3 for this insightful comment. In the first revised manuscript, we stated: "*the observed loss of cytokine responses to IETD in caspase-8/MLKL double knockout (DKO) mice cannot be attributed to a generalized defect in cytokine production, as these mice have been shown to exhibit normal or even enhanced responses to a wide range of pro-inflammatory stimuli*." In support of this statement, we cite the paper by Alvarez-Díaz mentioned by the reviewer. We respectfully believe that our statement does not represent a misinterpretation of the Alvarez-Díaz

paper. In fact, not only our statement but also one of the central features of our data—namely RIPK3-dependent cytokine production in the absence of necroptosis, as discussed above—appears to be fully consistent with the conclusions of Alvarez-Díaz *et al.*, as expressed in their own words at the end of the paper summary: "*These results … suggest that RIPK3 and/or MLKL may exert functions independently of necroptosis. It appears that non-necroptotic functions of RIPK3 contribute to the lymphadenopathy, autoimmunity, and* ***excess cytokine production*** *that occur when FADD- or caspase-8–mediated apoptosis is abrogated*."

The reviewer also notes that BMDMs from Casp8/MLKL DKO mice are hypo-responsive to poly(I:C) and LPS. However, this observation is not directly relevant to the present discussion. BMDMs from wild-type mice are completely non-responsive to IETD-induced caspase-8 inhibition, both in terms of cytokine gene induction and global gene regulation (Fig. 3). Importantly, Casp8/MLKL DKO mice exhibit elevated or normal cytokine levels under homeostatic conditions, as shown in the paper by Alvarez-Diaz *et al.* mentioned above. This indicates that they retain normal or even increased responses to endogenous autoinflammatory stimuli and/or the microbiota. Nevertheless, we have slightly toned down our statement that now reads: "*the observed loss of cytokine responses to IETD in caspase-8/MLKL double knockout (DKO) mice cannot be attributed to a generalized defect in cytokine production, as these mice have been shown to exhibit normal or even enhanced responses to pro-inflammatory stimuli*". In its present form, this statement appears justified, and we would prefer to retain it.

Reviewer #3

Comment. I'd like to thank the authors for the detailed and thoughtful answers to my queries. I believe this manuscript is suitable for publication in EMBO reports, should the authors address my remaining concerns/comments

Response. We are grateful to this reviewer for the insightful comments that greatly helped us improve the manuscript, and we will do our best to address the remaining concerns.

Comment 1 *.Regarding neutrophil elastase and cathepsin activity - I was suggesting that it would be important assess their activity through fluorogenic or chromogenic peptide substrate assays (e.g., Abcam, ab126780), as this would directly determine if there was increased activity up IETD treatment, and if this is reduced in RIPK3/MLKL KOs, rather than current inferences that are made only from expression data. I don't think the statement that "Our results show that IETD-induced increases in elastase activity are completely abolished in RIPK3 KO neutrophils (Fig. S6E)." is accurate, as this data represents an ELISA that measures elastase levels, not its activity.*

Response. We were unable to find the specific sentence mentioned by the reviewer in our manuscript. We made efforts to measure elastase activity using fluorogenic substrates, as suggested. While the elastase control worked well, we were not satisfied with the reproducibility of the assay using neutrophil lysates and supernatants. We suspect that this issue may be related to the abundance of other neutrophil proteases in these mixtures. On the other hand, serine proteases are believed to be stored in granules in an already active form in mature neutrophils, and therefore we did not expect dramatic effects of IETD in terms of activation, although this point may warrant further investigation. We were, however, pleased to observe clear increases in RIPK3-dependent protein expression after IETD treatment.

Comment 2. *The original Figure 7C and revised manuscript Figure 7C show completely different results. The original Figure 7C showed that IETD resulted in a loss of full length GSDMD and increased GSDMD p23 (with GSDMD p30 being almost undetectable), while the addition of MeOSuc, BAY85 and JNJ-103 all resulted in GSDMD p30 being retained (MeOSuc) or increased (BAY85 and JNJ-103) compared to controls, with BAY85 and JNJ-103 also increasing levels of full-length GSDMD. In contrast, the new Fig. 7C shows robust GDMD p30 generation upon IETD treatment (not disappearance), with MeOSuc, BAY85 and JNJ-103 all reducing levels of cleaved p30 GSDMD (not increasing). These contradictory results between the original and revised manuscript make it impossible to know what is going on. How many times has the experiment been reproduced and what were the results from each independent experiment?*

Response. Thank you once again for carefully assessing our western blot data. We are not convinced that the original Fig. 7 and the figure included in the revised manuscript show fundamentally different results, although our earlier blots were technically suboptimal, as previously acknowledged and further discussed below.

Both the original and the revised Fig. 7 demonstrate that IETD treatment induces GSDMD processing and that MeOSuc, BAY85, or JNJ-103 inhibit this processing. We believe this becomes clear when the gels are evaluated based on the ratio of processed to unprocessed bands in each lane, rather than by focusing on the molecular weight of individual processed bands. In our earlier experiments, we indeed observed variability between similarly treated samples in the apparent molecular weight of the processed bands (23–30 kDa). In addition, the total amount of immunoreactive GSDMD (i.e., the sum of processed and unprocessed bands) varied, even after normalization to the housekeeping protein.

After the initial submission, we worked to resolve these issues and substantially improved our experimental procedures. We concluded that the variability was likely caused by protease activity in cell lysates. GSDMD and its processed fragments appeared particularly sensitive to proteolysis in neutrophil lysates, which are rich in proteases. To address this, we introduced several technical improvements, including minimizing delays during sample processing and optimizing the storage conditions of the protease inhibitor cocktail.

Since implementing these changes, we have obtained much cleaner gels showing that IETD induces GSDMD processing, that this processing is consistently inhibited by serine protease inhibitors, and that the processed form displays a consistent molecular weight (~30-35 kDa), in agreement with the reported molecular weight of serin protease-processed forms of GSDMD. The experiment has been repeated twice with similar results.

Comment 3. *The authors write "However, previous work from our lab and from that of others has shown that immune cells from these mice produce normal or even elevated levels of proinflammatory cytokines in response to LPS, poly(I:C), and PMA plus ionomycin stimulation (Immunity, 2016, 45:513-526; Figs. 7C-D, S7-S8)." This statement is factually incorrect. First, there is no Fig. S8 in this paper. Second, Fig. S7 shows that caspase-8 and MLKL deletion reduces LPS and polyIC driven IL-1alpha, IL-1beta, IL-3, IL-6, IL-12, IL-13, IL-17, eotaxin, G-CSF, IFN-gamma, KC, to name a few. Infact, based on this paper one would argue that caspase-8 and MLKL loss reduces many, if not most, inflammatory factors, with CD3 plus CD8 antibody stimulated T-cell increased cytokines (eg RANTEs, IL-10) being the exception. To further write that "...Casp8-Mlkl*

double knockout mice completely retain their ability to respond to inflammatory stimuli and cite the above- mentioned reference (lines 124-127)." is misleading. While these mice develop autoimmune proliferative syndrome with age, this results from defective apoptosis and increased T-cell numbers, and a sub-set of increased T-cell cytokines. In fact, other studies have shown that caspase-8 and MLKL/RIPK3 loss in mice renders these animals incredibly resistant to damaging inflammation, because of the loss of caspase-8-driven cytokine responses (e.g., PMID: 41233351, PMID: 40632844).

Response. Thank you for this comment. We realize that the point we were trying to make in our previous response was not sufficiently clear, and we apologize for the lack of clarity. We believe that we addressed this issue more clearly in our response to Reviewer 2, which we reproduce below for ease of reference.

We thank this reviewer and Reviewer 3 for this insightful comment. In the revised manuscript, we stated: "*the observed loss of cytokine responses to IETD in caspase-8/MLKL double knockout (DKO) mice cannot be attributed to a generalized defect in cytokine production, as these mice have been shown to exhibit normal or even enhanced responses to a wide range of pro-inflammatory stimuli*." In support of this statement, we cite the paper by Alvarez-Díaz mentioned by the reviewer. We respectfully believe that this statement does not represent a misinterpretation of the Alvarez-Díaz paper. In fact, not only our statement but also one of the central features of our data—namely RIPK3-dependent cytokine production in the absence of necroptosis, as discussed above—appears to be fully consistent with the conclusions of Alvarez-Díaz *et al*., as expressed in their own words at the end of the paper summary: "*These results … suggest that RIPK3 and/or MLKL may exert functions independently of necroptosis. It appears that non-necroptotic functions of RIPK3 contribute to the lymphadenopathy, autoimmunity, and **excess cytokine production** that occur when FADD- or caspase-8–mediated apoptosis is abrogated*."

The reviewer also notes that BMDMs from Casp8/MLKL DKO mice are hypo-responsive to poly(I:C) and LPS. However, this observation is not directly relevant to the present discussion. BMDMs from wild-type mice are completely non-responsive to IETD-induced caspase-8 inhibition, both in terms of cytokine gene induction and global gene regulation (Fig. 3). Importantly, Casp8/MLKL DKO mice exhibit elevated or normal cytokine levels under homeostatic conditions, as shown in the paper by Alvarez-Diaz *et al* mentioned above. This indicates that they retain normal or even increased responses to endogenous autoinflammatory stimuli and/or the microbiota. Nevertheless, we have now slightly toned down our statement that now reads: "*the observed loss of cytokine responses to IETD in caspase-8/MLKL double knockout (DKO) mice cannot be attributed to a generalized defect in cytokine production, as these mice have been shown to exhibit normal or even enhanced responses to pro-inflammatory stimuli*". In its present form, this statement appears justified, and we would prefer to retain it.

Comment 4*. While the authors cite studies suggesting activated GSDMD and RIPK3 do not always result in death, I am questioning whether the authors own data suffice to draw the conclusion that their observations occur "without evidence of necroptosis, pyroptosis or other forms of cell death". As I noted in my original comments, the authors show markers of pyroptosis and necroptosis in neutrophils upon IETD treatment and the neutrophils are also still dying over time with and without IETD treatment. Fig 3. shows transcriptional changes at 3 hours. The corresponding death data in Fig. S7B/C show cell viability has reduced from ~90% to under 80% in IETD treated cells at 3 hrs. While I agree that the transcriptional changes are unlikely to reflect the impact of dying cells*

(although GSDMD-driven IL-1beta release may), I don't think the authors can state that IETD-mediated activation of GSDMD or RIPK3 is not causing death in this situation and to do so would require more detailed experimentation. As an example as to why this level of detail is required, I note the meticulous single cell analysis recently performed on human monocytes to show that death is a driver of IL-1 secretion in human monocytes, disproving previous high profile population-level evidence concluding that IL-1 secretion was death-independent (PMID: 41219490). Consequently, my recommendation would be that the authors tone down their conclusions on activated RIPK3 and GSDMD driving cytokine responses without cell death occurring downstream of RIPK3/GSDMD, and make it clear to readers that death may or may not occur via their activity and that further experimentation is required to resolve these two possibilities.

Response. Thank you for your insightful comment. As requested, we have toned down our conclusions regarding the death-independent nature of the observed changes. We now state at the end of the Discussion (lines 409-412): "Although IETD-induced caspase-8 inhibition did not increase cell death in neutrophil cultures at the population level, further observations involving single-cell analyses will be required to more precisely assess the relationship between caspase-8–regulated transcriptional activity and activation of cell death pathways."

POINT BY POINT DISPOSAL OF EDITORIAL REQUESTS

- **Expanded View format** (we have complied with all requests);
- **Key words** (they were reduced to five)
- **CRediT** (this was compiled in author contribution section of the online submission tool);
- **Number of independent experiments biological vs technical replicates, and other requirements** (all requirements were fulfilled);
- **Data availability section** (tokens were removed as requested);
- **References** (we used the requested reference format);
- **"Not shown"** (this was removed throughout the manuscript);
- **Structured Methods** (methods were structured as required);
- **Funding information** (included in the main text);
- **Resolution of the submitted figures** (we have complied with all recommendation) ;
- **Values for vehicle controls** (the reason why the same numbers appear in many of these data set is that values below the detection threshold were arbitrarily given a value corresponding to half of the limit of detection of the assay. This is a standard procedure to enable statistical analysis);
- **Brief summary of the manuscript** (included in the appendix file);
- **Bullet points** (included in the appendix file);
- **Synopsis** (we have complied with all suggestions).

Dear Prof. Teti

Thank you for the submission of your further revised manuscript to our editorial offices. I have now received the report from the referee that was asked to re-evaluate the study, you will find below. As you will see, the referee now supports the publication of the study in EMBO reports. However, the referee has 2 final comments (points 2 and 3). Please address these in a final revised manuscript and a final p-b-p-response.

Moreover, I have some further editorial requests:

- Please remove the 'running head' from the title page.

- There are mentions of a 'resource table' (line 573) and of a 'materials table' (line 591) in the main manuscript text. Please replace these by callouts to the 'Reagents and Tools Table'. Please also add more callouts to this table where appropriate.

- Please also confirm that for all Western blot panels shown the loading control was run on the same gel as the other proteins detected. Please note that we discourage comparisons between samples on different gels/blots, even if the samples derive from one experiment, as confounding factors reduce comparability. If unavoidable, the figure legend must state that the samples derive from the same experiment and that gels/blots were processed in parallel. If a 'representative' loading control is shown for multiple gels/blots, the intra-gel controls should be shown in the source data files, and the figure legends should describe the data displayed accurately. See our author guidelines:

https://link.springer.com/journal/44319/submission-guidelines#cms-Figure-and-data-presentation (section 'Electrophoretic gels and blots').

I look forward to seeing the final revised version of your manuscript when it is ready. Please let me know if you have questions regarding the revision.

Best,

Achim Breiling
Senior Editor
EMBO Reports

Referee #2:

1. This was addressed. I would just note that my quote was accurate and was written by the authors themselves - so I'm surprised they did not realise. It referred to the authors writing in their first response (not manuscript): "Thanks for this suggestion. We have now assessed elastase levels in IETD-treated neutrophils from RIPK3 KO mice. Our results show that IETD-induced increases in elastase activity are completely abolished in RIPK3 KO neutrophils (Fig. S6E)."

2. The authors write "BMDMs from wild-type mice are completely non-responsive to IETD-induced caspase-8 inhibition, both in terms of cytokine gene induction and global gene regulation (Fig. 3)." I can't see any BMDM data in Figure 3 so can't evaluate this data/statement.

3. I do not think the authors can state that "the observed loss of cytokine responses to IETD in caspase-8/MLKL double knockout (DKO) mice cannot be attributed to a generalized defect in cytokine production, as these mice have been shown to exhibit normal or even enhanced responses to pro-inflammatory stimuli". This is because it is simply not true - caspase-8 co-deletion with RIPK3/MLKL results in a huge defect in responses to inflammatory stimuli - as I noted previously and as shown in the Alvarez-Diaz paper and other papers I mentioned. To say they are normal is wrong.

4. This was addressed.

Disposal of the Editor comments

The running title was removed from the front page, introduced callouts to the "Reagents and Tools Table" and indicated in the Figure Legends, where appropriate, that the "β-actin blots, used as loading controls, derive from the same experiment and were processed in parallel"

Point by point disposal of reviewer no.2 comments

1.Comment . *This was addressed. I would just note that my quote was accurate and was written by the authors themselves - so I'm surprised they did not realise. It referred to the authors writing in their first response (not manuscript): "Thanks for this suggestion. We have now assessed elastase levels in IETD-treated neutrophils from RIPK3 KO mice. Our results show that IETD-induced increases in elastase activity are completely abolished in RIPK3 KO neutrophils (Fig. S6E)."*
Response. We are glad the reviewer thinks we have now addressed this point. Sorry for the confusion ("activity" instead of "levels") in our first response

2. Comment. *The authors write "BMDMs from wild-type mice are completely non-responsive to IETD-induced caspase-8 inhibition, both in terms of cytokine gene induction and global gene regulation (Fig. 3)." I can't see any BMDM data in Figure 3 so can't evaluate this data/statement.*
Response. Apologies. The data on macrophages are shown in Fig. EV2A and reported in lines 163 and 164

3. Comment. *responses to IETD in caspase-8/MLKL double knockout (DKO) mice cannot be attributed to a generalized defect in cytokine production, as these mice have been shown to exhibit normal or even enhanced responses to pro-inflammatory stimuli". This is because it is simply not true - caspase-8 co-deletion with RIPK3/MLKL results in a huge defect in responses to inflammatory stimuli - as I noted previously and as shown in the Alvarez-Diaz paper and other papers I mentioned. To say they are normal is wrong.*
Response. We have now removed this sentence from the paper

4. Comment. *This was addressed.*
Response. We are glad the reviewer thinks we have now addressed this point.

Prof. Giuseppe Teti
Scylla Biotech Srl
Research and Development
Messina
Italy

Dear Prof. Teti,

I am very pleased to accept your manuscript for publication in the next available issue of EMBO reports. Thank you for your contribution to our journal.

Your manuscript will be processed for publication by EMBO Press. It will be copy edited and you will receive page proofs prior to publication. Please note that you will be contacted by Springer Nature Author Services to complete licensing and payment information.

You may qualify for financial assistance for your publication charges - either via a Springer Nature fully open access agreement or an EMBO initiative. Check your eligibility: https://link.springer.com/journal/44319/how-to-publish-with-us

Should you be planning a Press Release on your article, please notify the production team via this form in order to coordinate publication and release dates: https://forms.cloud.microsoft/e/eH4A8JVP6g

If you have any questions, please do not hesitate to contact the Editorial Office. Thank you for your contribution to EMBO Reports.

Yours sincerely,

Achim Breiling
Senior Editor
EMBO Reports

>>> Please note that it is EMBO Reports policy for the transcript of the editorial process (containing referee reports and your response letter) to be published as an online supplement to each paper. If you do NOT want this, you will need to inform the Editorial Office via email immediately. More information is available here: https://link.springer.com/partners/embo-press/editorial-policies#Peer%20review

EMBO Press Author Checklist

Corresponding Author Name: Giuseppe Teti	
Journal Submitted to: EMBO Reports	
Manuscript Number: EMBOR-2025-62947-T.	

USEFUL LINKS FOR COMPLETING THIS FORM
The EMBO Journal - Author Guidelines
EMBO Reports - Author Guidelines
Molecular Systems Biology - Author Guidelines
EMBO Molecular Medicine - Author Guidelines

Reporting Checklist for Life Science Articles (updated January
This checklist is adapted from Materials Design Analysis Reporting (MDAR) Checklist for Authors. MDAR establishes a minimum set of requirements in transparent reporting in the life sciences (see Statement of Task: 10.31222/osf.io/9sm4x). Please follow the journal's guidelines in preparing your
Please note that a copy of this checklist will be published alongside your article.

Abridged guidelines for figures
1. Data
The data shown in figures should satisfy the following conditions:
- the data were obtained and processed according to the field's best practice and are presented to reflect the results of the experiments in an accurate and unbiased manner.
- ideally, figure panels should include only measurements that are directly comparable to each other and obtained with the same assay.
- plots include clearly labeled error bars for independent experiments and sample sizes. Unless justified, error bars should not be shown for technical
- if n<5, the individual data points from each experiment should be plotted. Any statistical test employed should be justified.
- Source Data should be included to report the data underlying figures according to the guidelines set out in the authorship guidelines on Data

2. Captions
Each figure caption should contain the following information, for each panel where they are relevant:
- a specification of the experimental system investigated (eg cell line, species name).
- the assay(s) and method(s) used to carry out the reported observations and measurements.
- an explicit mention of the biological and chemical entity(ies) that are being measured.
- an explicit mention of the biological and chemical entity(ies) that are altered/varied/perturbed in a controlled manner.
- the exact sample size (n) for each experimental group/condition, given as a number, not a range;
- a description of the sample collection allowing the reader to understand whether the samples represent technical or biological replicates (including how many animals, litters, cultures, etc.).
- a statement of how many times the experiment shown was independently replicated in the laboratory.
- definitions of statistical methods and measures:
  - common tests, such as t-test (please specify whether paired vs. unpaired), simple $\chi 2$ tests, Wilcoxon and Mann-Whitney tests, can be unambiguously identified by name only, but more complex techniques should be described in the methods section;
  - are tests one-sided or two-sided?
  - are there adjustments for multiple comparisons?
  - exact statistical test results, e.g., P values = x but not P values < x;
  - definition of 'center values' as median or average;
  - definition of error bars as s.d. or s.e.m.

Please complete ALL of the questions below.
Select "Not Applicable" only when the requested information is not relevant for your study.

Materials

Newly Created Materials	Information included in the manuscript?	In which section is the information available? (Reagents and Tools Table, Materials and Methods, Figures, Data Availability Section)
New materials and reagents need to be available; do any restrictions apply?	Not Applicable	

Antibodies	Information included in the manuscript?	In which section is the information available? (Reagents and Tools Table, Materials and Methods, Figures, Data Availability Section)
For **antibodies** provide the following information:<br>- Commercial antibodies: RRID (if possible) or supplier name, catalogue number and or/clone number<br>- Non-commercial: RRID or citation	Yes	Reagents and Tools Table

DNA and RNA sequences	Information included in the manuscript?	In which section is the information available? (Reagents and Tools Table, Materials and Methods, Figures, Data Availability Section)
**Short novel DNA or RNA including primers, probes**: provide the sequences.	Not Applicable	

Cell materials	Information included in the manuscript?	In which section is the information available? (Reagents and Tools Table, Materials and Methods, Figures, Data Availability Section)
**Cell lines:** Provide species information, strain. Provide accession number in repository **OR** supplier name, catalog number, clone number, and/**OR** RRID.	Not Applicable	
**Primary cultures:** Provide species, strain, sex of origin, genetic modification status.	Yes	Materials and Methods
Report if the cell lines were recently **authenticated** (e.g., by STR profiling) and tested for mycoplasma contamination.	Not Applicable	

Experimental animals	Information included in the manuscript?	In which section is the information available? (Reagents and Tools Table, Materials and Methods, Figures, Data Availability Section)
**Laboratory animals or Model organisms:** Provide species, strain, sex, age, genetic modification status. Provide accession number in repository **OR** supplier name, catalog number, clone number, **OR** RRID.	Yes	Reagents Tools Table and Materials and Methods
**Animal observed in or captured from the field:** Provide species, sex, and age where possible.	Not Applicable	
Please detail **housing and husbandry conditions**.	Yes	Materials and Methods

Plants and microbes	Information included in the manuscript?	In which section is the information available? (Reagents and Tools Table, Materials and Methods, Figures, Data Availability Section)
**Plants:** provide species and strain, ecotype and cultivar where relevant, unique accession number if available, and source (including location for collected wild specimens).	Not Applicable	
**Microbes:** provide species and strain, unique accession number if available, and source.	Not Applicable	

Human research participants	Information included in the manuscript?	In which section is the information available? (Reagents and Tools Table, Materials and Methods, Figures, Data Availability Section)
If collected and within the bounds of privacy constraints report on age, sex and gender or ethnicity for all study participants.	Not Applicable	

Core facilities	Information included in the manuscript?	In which section is the information available? (Reagents and Tools Table, Materials and Methods, Figures, Data Availability Section)
If your work benefited from core facilities, was their service mentioned in the acknowledgments section?	Yes	Acknowledgments

Design

Study protocol	Information included in the manuscript?	In which section is the information available? (Reagents and Tools Table, Materials and Methods, Figures, Data Availability Section)
If study protocol has been **pre-registered, provide DOI in the manuscript**. For clinical trials, provide the trial registration number **OR** cite DOI.	Not Applicable	
Report the **clinical trial registration number** (at ClinicalTrials.gov or equivalent), where applicable.	Not Applicable	

Laboratory protocol	Information included in the manuscript?	In which section is the information available? (Reagents and Tools Table, Materials and Methods, Figures, Data Availability Section)
Provide DOI OR other citation details if **external detailed step-by-step protocols** are available.	Not Applicable	

Experimental study design and statistics	Information included in the manuscript?	In which section is the information available? (Reagents and Tools Table, Materials and Methods, Figures, Data Availability Section)
Include a statement about **sample size** estimate even if no statistical methods were used.	Yes	Figure Legends
Were any steps taken to minimize the effects of subjective bias when allocating animals/samples to treatment (e.g. **randomization procedure**)? If yes, have they been described?	Yes	Materials and Methods
Include a statement about **blinding** even if no blinding was done.	Yes	Materials and Methods
Describe **inclusion/exclusion criteria** if samples or animals were excluded from the analysis. Were the criteria pre-established?<br><br>If sample or data points were omitted from analysis, report if this was due to attrition or intentional exclusion and provide justification.	Not Applicable	
For every figure, are **statistical tests** justified as appropriate? Do the data meet the assumptions of the tests (e.g., normal distribution)? Describe any methods used to assess it. Is there an estimate of variation within each group of data? Is the variance similar between the groups that are being statistically compared?	Yes	Figure Legends

Sample definition and in-laboratory replication	Information included in the manuscript?	In which section is the information available? (Reagents and Tools Table, Materials and Methods, Figures, Data Availability Section)
In the figure legends: state number of times the experiment was **replicated** in laboratory.	Yes	Figure Legends
In the figure legends: define whether data describe **technical or biological replicates**.	Yes	Figure Legends

Ethics

Ethics	Information included in the manuscript?	In which section is the information available? (Reagents and Tools Table, Materials and Methods, Figures, Data Availability Section)
Studies involving **human participants**: State details of **authority granting ethics approval** (IRB or equivalent committee(s), provide reference number for approval.	Not Applicable	
Studies involving **human participants**: Include a statement confirming that **informed consent** was obtained from all subjects and that the experiments conformed to the principles set out in the WMA Declaration of Helsinki and the Department of Health and Human Services Belmont Report.	Not Applicable	
Studies involving **human participants:** For publication of **patient photos**, include a statement confirming that consent to publish was obtained.	Not Applicable	
Studies involving experimental **animals**: State details of **authority granting ethics approval** (IRB or equivalent committee(s), provide reference number for approval. Include a statement of compliance with ethical regulations.	Yes	Materials and Methods
Studies involving **specimen and field samples:** State if relevant **permits** obtained, provide details of authority approving study; if none were required, explain why.	Not Applicable	

Dual Use Research of Concern (DURC)	Information included in the manuscript?	In which section is the information available? (Reagents and Tools Table, Materials and Methods, Figures, Data Availability Section)
Could your study fall under dual use research restrictions? Please check biosecurity documents and list of **select agents and toxins** (CDC): https://www.selectagents.gov/sat/list.htm	Not Applicable	
If you used a select agent, is the security level of the lab appropriate and reported in the manuscript?	Not Applicable	
If a study is subject to dual use research of concern regulations, is the name of the **authority granting approval and reference number** for the regulatory approval provided in the manuscript?	Not Applicable	

Reporting

The MDAR framework recommends adoption of discipline-specific guidelines, established and endorsed through community initiatives. Journals have their own policy about requiring specific guidelines and recommendations to complement MDAR.

Adherence to community standards	Information included in the manuscript?	In which section is the information available? (Reagents and Tools Table, Materials and Methods, Figures, Data Availability Section)
State if relevant guidelines or checklists (e.g., **ICMJE, MIBBI, ARRIVE, PRISMA**) have been followed or provided.	Yes	Materials and Methods
For **tumor marker prognostic studies,** we recommend that you follow the **REMARK** reporting guidelines (see link list at top right). See author guidelines, under 'Reporting Guidelines'. Please confirm you have followed these guidelines.	Not Applicable	
For **phase II and III randomized controlled trials**, please refer to the **CONSORT** flow diagram (see link list at top right) and submit the CONSORT checklist (see link list at top right) with your submission. See author guidelines, under 'Reporting Guidelines'. Please confirm you have submitted this list.	Not Applicable	

Data Availability

Data availability	Information included in the manuscript?	In which section is the information available? (Reagents and Tools Table, Materials and Methods, Figures, Data Availability Section)
Have **primary datasets** been deposited according to the journal's guidelines (see 'Data Deposition' section) and the respective accession numbers provided in the Data Availability Section?	Yes	Data Availability Section
Were **human clinical and genomic datasets** deposited in a public access-controlled repository in accordance to ethical obligations to the patients and to the applicable consent agreement?	Not Applicable	
Are **computational models** that are central and integral to a study available without restrictions in a machine-readable form? Were the relevant accession numbers or links provided?	Not Applicable	
If publicly available data were reused, provide the respective **data citations in the reference list**.	Not Applicable	
